# An endoplasmic reticulum stress-regulated lncRNA hosting a microRNA megacluster induces early features of diabetic nephropathy

**DOI:** 10.1038/ncomms12864

**Published:** 2016-09-30

**Authors:** Mitsuo Kato, Mei Wang, Zhuo Chen, Kirti Bhatt, Hyung Jung Oh, Linda Lanting, Supriya Deshpande, Ye Jia, Jennifer Y.C. Lai, Christopher L. O’Connor, YiFan Wu, Jeffrey B. Hodgin, Robert G. Nelson, Markus Bitzer, Rama Natarajan

**Affiliations:** 1 Department of Diabetes Complications and Metabolism, Diabetes Metabolism Research Institute, Beckman Research Institute of City of Hope, Duarte, California 91010, USA; 2 Department of Internal Medicine, University of Michigan, Ann Arbor, Michigan 48109, USA; 3 Department of Pathology, University of Michigan, Ann Arbor, Michigan 48109, USA; 4 Diabetes Epidemiology and Clinical Research Section, National Institute of Diabetes and Digestive and Kidney Diseases, National Institutes of Health, Phoenix, Arizona 85014, USA

## Abstract

It is important to find better treatments for diabetic nephropathy (DN), a debilitating renal complication. Targeting early features of DN, including renal extracellular matrix accumulation (ECM) and glomerular hypertrophy, can prevent disease progression. Here we show that a megacluster of nearly 40 microRNAs and their host long non-coding RNA transcript (lnc-MGC) are coordinately increased in the glomeruli of mouse models of DN, and mesangial cells treated with transforming growth factor-β1 (TGF- β1) or high glucose. Lnc-MGC is regulated by an endoplasmic reticulum (ER) stress-related transcription factor, CHOP. Cluster microRNAs and lnc-MGC are decreased in diabetic *Chop*^−/−^ mice that showed protection from DN. Target genes of megacluster microRNAs have functions related to protein synthesis and ER stress. A chemically modified oligonucleotide targeting lnc-MGC inhibits cluster microRNAs, glomerular ECM and hypertrophy in diabetic mice. Relevance to human DN is also demonstrated. These results demonstrate the translational implications of targeting lnc-MGC for controlling DN progression.

## Abstract (web summary)

Nephropathy is a common and hard-to-treat consequence of diabetes. Here Kato *et al*. show that a megacluster of microRNAs regulates early development of diabetic nephropathy in mice, and that inhibition of the cluster's host long non-coding RNA transcript attenuates disease symptoms, suggesting a new therapy for diabetic nephropathy.

## Graphical abstract

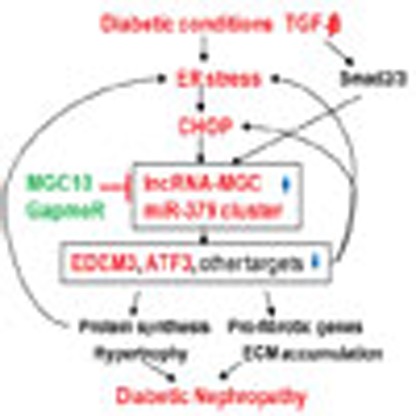

Diabetic nephropathy (DN) is one of the most common and debilitating complications of diabetes for which better therapies are sorely needed. The ‘early’ features of DN include glomerular mesangial expansion and hypertrophy, increased renal accumulation of extracellular matrix (ECM) proteins such as collagens and fibronectin, as well as podocyte effacement^1^,^2^. The expression of profibrotic transforming growth factor-β1 (TGF-β1) is increased in mesangial cells (MCs) and other renal cells in diabetes and mediates many of these adverse effects in DN. Factors associated with the pathogenesis of DN such as angiotensin II and high glucose (HG) increase TGF-β1 expression in MCs *in vitro* and *in vivo*^3^,^4^,^5^,^6^,^7^. Signals from the activated TGF-β1 receptor complex are transduced to the nucleus by Smad proteins, which regulate TGF-β1-induced gene expression^8^. However, it is not fully clear how diabetic conditions and TGF-β1 regulate the genes that increase the hypertrophy, protein synthesis and fibrosis associated with DN.

MicroRNAs (miRNAs) are endogenously produced, short non-coding RNAs (∼20–22 nucleotides) that play key roles in post-transcriptional regulation of gene expression. miRNAs silence genes by repressing their translation or inducing the degradation of target mRNAs^9^. Since nearly 60% of mRNAs in the genome are targeted by miRNAs, they regulate many cellular functions and pathophysiological states including kidney diseases^6^,^9^,^10^. Several miRNAs have been implicated in the pathogenesis of DN and also as possible biomarkers or therapeutic targets^2^,^6^,^10^. Antisense inhibitors have been widely used to inhibit specific miRNAs^11^,^12^,^13^ and some clinical trials are ongoing^14^,^15^. Inhibition of key miRNAs by chemically modified antisense oligonucleotides or by genetic knockout (KO) attenuated early manifestations of DN in mice (ECM accumulation and glomerular hypertrophy) and albuminuria (a later feature of DN)^2^,^6^,^10^,^11^,^12^,^16^,^17^,^18^,^19^,^20^,^21^. Therefore, targeting specific miRNAs that mediate the early features of DN could be an effective approach to prevent progression of DN. In this study, we observed that an unique megacluster of nearly 40 miRNAs were coordinately increased along with their host transcript (a long non-coding RNA (lncRNA)) in the diabetic kidney and mediated several features of DN. Furthermore, inhibition of the host transcript decreased the expression of the cluster miRNAs, and attenuated early features of DN *in vitro* in MCs, and *in vivo* in mice.

## Results

### Cluster miRNAs and host RNA are increased in diabetic kidney

**Figure 1:**
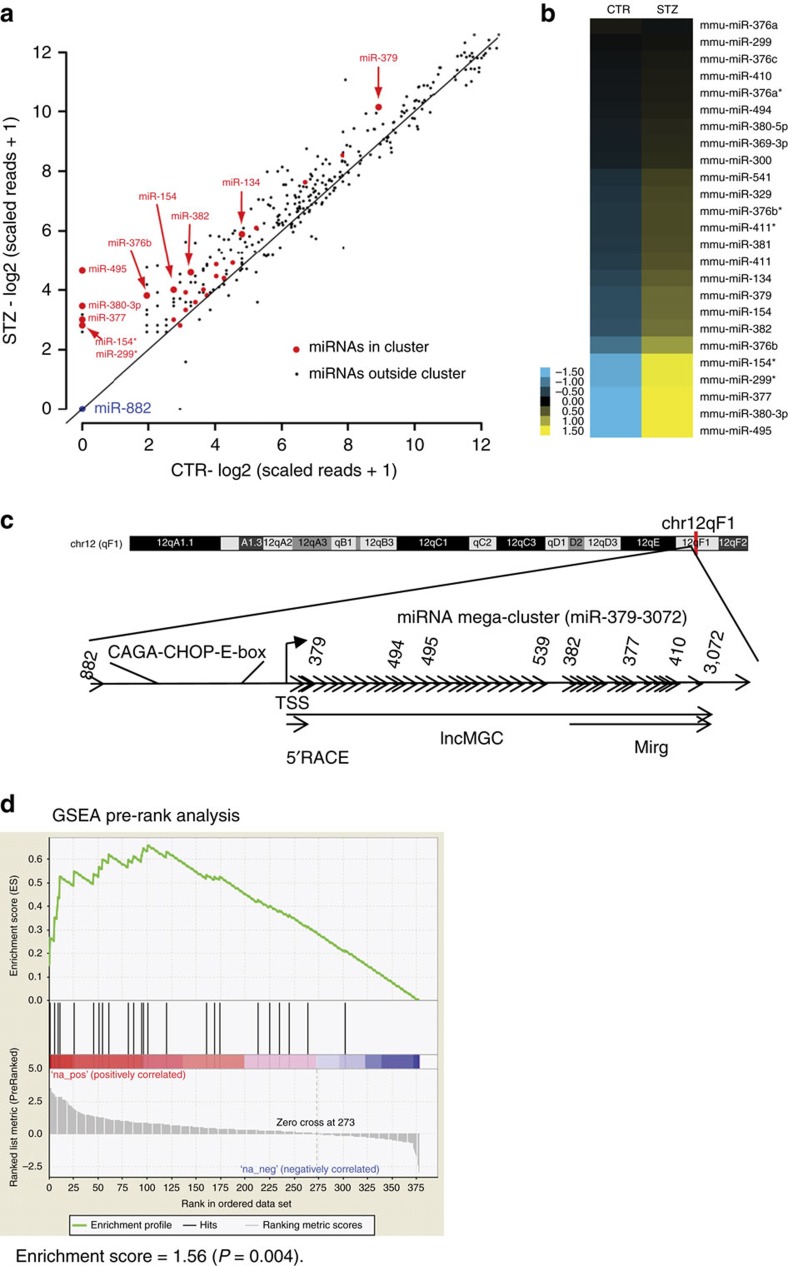
The miR-379 miRNA megacluster is increased in the glomeruli of diabetic mice. Small RNA (smRNA) sequencing was performed as previously described^22^. (**a**) Scatter plot of miRNAs in kidney glomeruli from control (CTR, vehicle injected) and diabetic mice (STZ injected; 4 weeks post diabetes). The expression of each detectable miRNA in the form of log scaled reads was plotted with *x* axis for CTR and *y* axis for STZ. Each dot represents one miRNA. miRNAs in the miR-379 cluster were presented in red. Among these, the miRNAs upregulated by fold change ≥2 are highlighted with bigger size dots and labelled with the corresponding miRNA names. miR-882 was plotted in blue as a negative control (outside the miR-379 cluster). (**b**) Heatmap of the miR-379 cluster miRNAs in CTR and STZ samples. The expression of each detectable miRNA within the miR-379 cluster in the two samples (CTR and STZ) were ordered by log2 fold change from low to high, mean-centered and shown in the heatmap. Blue represents lower than average expression in the two samples and yellow presents higher than average expression level. The expression of the detected miRNAs in this cluster was higher in STZ than CTR. (**c**) Genome structure of the mouse miR-379 megacluster region. This cluster is located within the largest miRNA cluster currently identified in the genome. It maps within the *DLK-DIO3* genomic region (mouse chr 12, human chr14), which is home to several miRNAs and lncRNAs. TSS, transcription start site. miR-882 is located far-upstream of the miR-379 cluster and not covered by lnc-MGC. (**d**) Gene Set Enrichment Analysis (GSEA). All the miRNAs detected by smRNA-seq with at least 5 scaled reads in at least one sample are ranked by log2 fold change between STZ and CTR samples to generate ranked list and all the detectable miRNAs in the cluster are considered as a gene set. Pre-ranked gene set analysis (GSEA) applied on the gene set using the ranked list of all the miRNAs revealed that miRNAs in the miR-379 cluster were significantly enriched within the miRNAs upregulated in the STZ diabetic mice, with normalized enrichment score of 1.56 (*P*=0.004).

**Figure 2:**
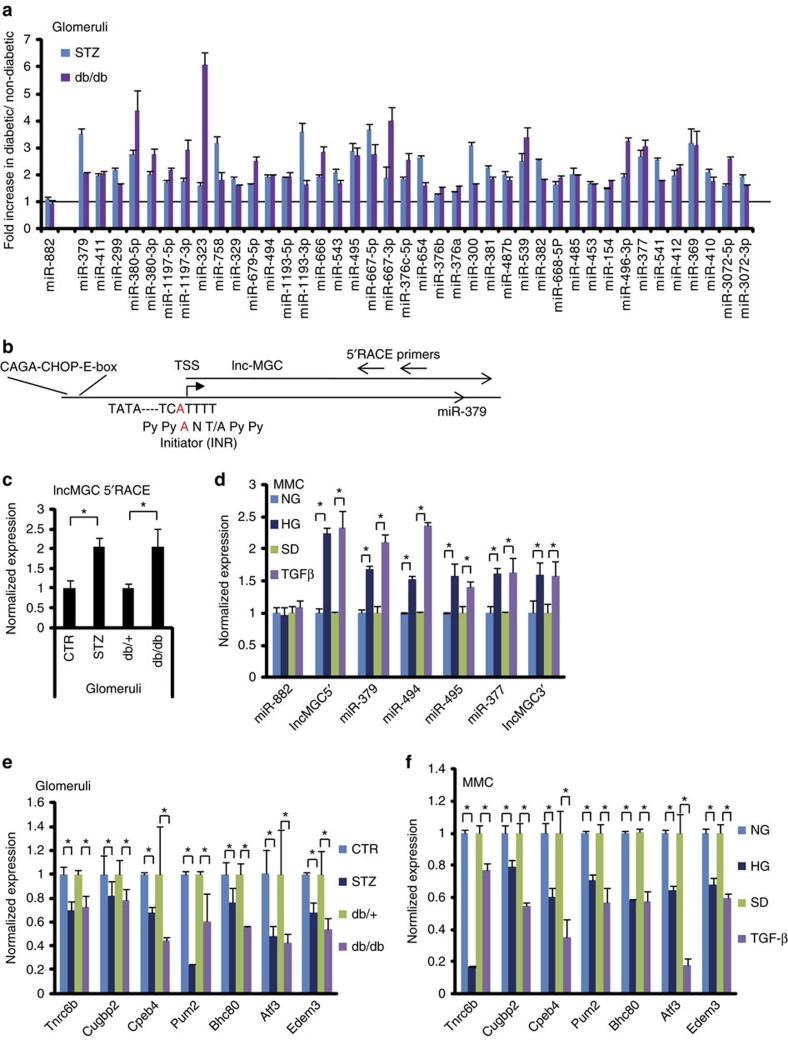
Lnc-MGC and cluster miRNAs are upregulated under diabetic conditions. (**a**) Primers for almost all (∼40) miRNAs in miR-379 cluster were designed and the expression of 40 miRNAs were confirmed by RT-PCRs and found to be increased significantly, Benjamini–Hochberg adjusted *P*<0.05 (ref. 69) in glomeruli from diabetic mice (STZ, STZ-injected type 1 model, blue bars; db/db, type 2 diabetic model, red bars) compared with respective non-diabetic mice (vehicle injected or db/+). Five mice in each group. Results are mean+s.e. from triplicate PCRs. No significant change was detected in the expression of miR-882 (outside of the miR-379 cluster). (**b**) 5′RACE of the region upstream of miR-379 identified the typical consensus sequence of initiator (INR; PyPyANT/APyPy) at the transcription start site. Smad-binding elements (CAGA repeats) and CHOP binding consensus and E-box sequences were found upstream of miR-379. (**c**) The expression of lnc-MGC in glomeruli from diabetic mice (STZ and db/db) compared with corresponding controls (vehicle injected CTR and db/+). Five mice in each group. *P<0.05, (**d**) The expression of lnc-MGC and cluster miRNAs in MMC treated with 25 mM HG (72 h) or 10 ng ml^−1^ TGF-β1 (24 h) compared with respective controls normal glucose (5.5 mM) or SD. respectively. (**e**) Significant lower expression of potential targets of miR-379 cluster in kidney glomeruli from diabetic mice (type 2 db/db and type 1 STZ) than the respective non-diabetic control mice. Five mice in each group. *P<0.05. (**f**) Decrease of potential targets of miR-379 cluster in MMC treated with TGF-β1 or HG. For experiments with MMC, results are mean+s.e. in triplicate PCRs from three independent culture experiments. *P<0.05.

RNA samples from the glomeruli of diabetic (streptozotocin (STZ) injected) and control mice were profiled by small RNA (miRNA)-sequencing and analysed by our methods^22^. In the diabetic mice glomeruli, we found a significant increase in the expression of numerous miRNAs that are among the megacluster of miRNAs within the *DLK-DIO3* genomic region (mouse chr12, human chr14)^23^, while miR-822 located outside of this cluster was unaltered (Fig. 1a–c and Supplementary Fig. 1). Gene Set Enrichment analyses (GSEA)^24^ indicated that these cluster miRNAs were significantly enriched among the miRNAs increased in the diabetic mice (Fig. 1d). PCR validation revealed significant increase in the levels of 40 of these cluster miRNAs in the glomeruli of both STZ injected and db/db mice (models of types 1 and 2 diabetes, respectively) compared with their corresponding controls (Fig. 2a). Genome organization shows that the megacluster (MGC) is hosted by a lncRNA, termed ‘lnc-MGC’, 3’ region of which overlaps with Mirg and middle region with Gm2922, other ncRNAs (Fig. 1c and Supplementary Fig. 1). miR-379 is located at the 5′ end, miR-494 and miR-495 in the middle, and miR-377 downstream (Fig. 1c). The 5′ region of lnc-MGC was cloned by 5′ rapid amplification of complementary DNA (cDNA) ends (RACE; Fig. 2b). This lnc-MGC host transcript was also increased in the glomeruli of diabetic mice (Fig. 2c). TGF-β1 and HG significantly increased the expression of miR-379, miR-494, miR-495 and miR-377 as well as lnc-MGC, but not miR-882, compared with serum depleted (SD) or normal glucose (NG) controls respectively in cultured mouse MC (MMC; Fig. 2d).

Since copy numbers of lncRNAs are in general relatively low (for example, *HOTTIP* copy number is 0.3 per cell)^25^, we calculated the copy numbers of the 5′ region of lnc-MGC cloned by us. Lnc-MGC was expressed at 3–4 copies per cell in control MMC, and 4–6 copies per cell in HG or TGF-β treated MMC (Supplementary Fig. 2), suggesting adequate expression of lnc-MGC for further study. Although miR-377and miR-382 have been implicated in renal fibrosis and HG response^26^,^27^, our data show that multiple miRNAs of the megacluster and host lncRNA are concomitantly increased in the glomeruli and MCs under diabetic conditions. Levels of these cluster miRNAs as well as lnc-MGC were also higher in the renal cortex and glomeruli of diabetic mice with more advanced diabetic kidney disease including significant proteinuria (22 weeks after onset of diabetes)^17^ (Supplementary Fig. 3a). These results suggest that the cluster miRNAs are co-regulated with the host lnc-MGC and may play key roles in the pathogenesis of DN. To date only few lncRNAs have been reported as hosts for some miRNAs under diabetic conditions, for e.g., RP23-298H6.1-001 hosting miR-216a and miR-217 (refs 6, 11, 28, 29, 30).

### Targets of the miRNA cluster

miRNA target prediction algorithms indicated that miRNAs in the megacluster have several common target genes. Therefore, the 3′ UTRs of at least 25 genes are similarly targeted by 8–13 cluster miRNAs, with some genes having more than two miRNA target sites (Supplementary Fig. 4a). These targets include RNA binding proteins and translational regulators including *CUGBP2* (ref. 31), *PUM2* (ref. 32), *TNRC6B* (ref. 33), *CPEB4* (ref. 34), *HuR* (ref. 35), transcription factors and co-factors (*Arid2*, *BHC80* (ref. 36), *NF1a/b*), *ATF3*, Endoplasmic Reticulum (ER) Degradation Enhancer, Mannosidase Alpha-Like 3 (*EDEM3*)^37^ and tumour suppressor phosphatase and tensin homologue (*PTEN*)^38^. *In silico* Ingenuity Pathway Analyses (IPA) suggested that these target gene sets have properties related to various molecular and cellular functions, human diseases, TGF-β signalling and other pathways, renal development/function and renal toxicity (Supplementary Figs 4b–f and 5a–e). Furthermore, IPA network analyses also suggested strong activation of Akt kinase (as well as Akt regulators phosphatidyl inositol-3kinase and *PTEN*) and Erk kinase (Supplementary Fig. 6a,b), known regulators of cellular hypertrophy and ECM accumulation. Some of the identified target genes also regulate protein translation, protein synthesis, mRNA stability and miRNA processing^39^. Interestingly, BHC80 interacts with histone deactylases and Ets to promote chromatin condensation^28^,^36^. ATF3 is a repressor of C/EBP homologous protein (CHOP), which is involved in ER stress^40^,^41^,^42^ and a direct target of miR-494 (ref. 43), a miRNA in the miR-379 cluster that we confirmed to be increased in diabetic mice glomeruli. The binding sites of multiple cluster miRNAs in the 3′UTRs of seven target genes (human and mouse) relevant to DN, *namely EDEM3, ATF3, TNRC6B, CUGBP2, CPEB4* and *PHF21A* are shown in Supplementary Figs 7–20 (obtained from TargetScan and microRNA.org). Therefore, the genes targeted by megacluster miRNAs have several functions related to DN, protein synthesis, ER stress, RNA binding and protein translation (Supplementary Figs 4–6).

The mRNA levels of *Tnrc6b, Cugbp2, Cpeb4, Pum2, Bhc80, Atf3* and *Edem3* were significantly reduced in the glomeruli from diabetic mice *in vivo* (db/db and STZ) compared with respective control mice (Fig. 2e), and in MMC treated with TGF-β1 or HG *in vitro* (Fig. 2f) compared with SD or normal glucose controls respectively. These targets have the potential to mediate the downstream effects of diabetes in a synergistic manner to augment renal hypertrophy and fibrosis in DN.

### Upstream region of the miRNA megacluster and host lnc-MGC

**Figure 3:**
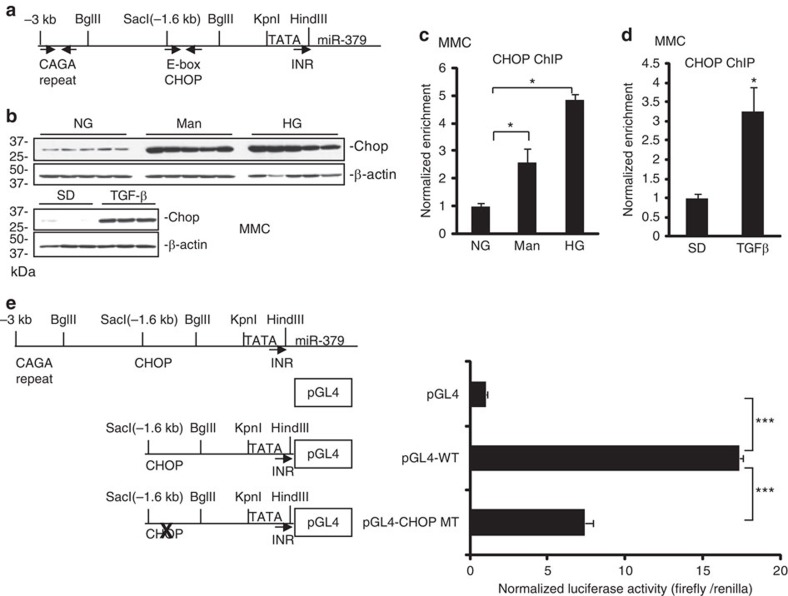
Regulatory role of CHOP. (**a**) Genomic structure of the region upstream of miR-379. CAGA repeats (potential Smad-binding elements) were identified at ∼3 kb upstream of miR-379 and CHOP binding element and E-boxes were found close to a SacI restriction enzyme site at ∼1.6 kb upstream of miR-379. (**b**) Chop protein levels were increased in MMC treated with TGF-β1, HG or mannitol compared with respective controls. Wider (uncropped) scans are shown in Supplementary Fig. 38. (**c**,**d**) ChIP assays and ChIP-real-time PCRs. CHOP occupancy was enriched at the CHOP binding site in MMC treated with HG or mannitol (**c**) or TGF-β1 (**d**). Results are mean+s.e. in triplicate PCRs from three independent ChIP experiments. *P<0.05. (**e**) The upstream (−1.6 kb) region, or the same region with mutated CHOP binding site, was cloned into the luciferase reporter pGL4 and plasmids were transfected into MMC. The WT plasmid showed significantly higher luciferase activity than empty pGL4 and mutation of CHOP site (MT) significantly reduced promoter activity. Results are mean+s.e. from triplicate reads of four independent cultures. ***P<0.001.

Apart from the region upstream of lnc-MGC, no other clear putative promoter was evident throughout the miRNA cluster, suggesting lnc-MGC is a long transcript which serves as host for the cluster miRNAs. To identify a putative promoter that drives the expression of this genomic region, we carried out 5′ RACE experiments using primers spanning the miR-379 upstream region (Fig. 2b). This region has promoter-like features with TATA box and initiator (INR) consensus sequences^44^. CAGA repeats (Smad-binding elements) were found ∼3 kb upstream of miR-379 (Fig. 3a), suggesting regulation by TGF-β1. Chromatin immunoprecipitation (ChIP) assays showed a significant increase in Smad2/3 occupancy at the miR-379 promoter Smad-binding sites (arrows, Fig. 3a) in MMCs treated with TGF-β1 compared with SD control (Supplementary Fig. 21a). Smad2/3 occupancy peaked at 1 h, and returned to normal by 24 h, suggesting the miR-379 cluster is regulated by TGF-β1 through rapid enrichment of Smad2/3 at the upstream region. We also identified binding sites for CHOP, a transcription factor associated with the ER stress response, and an overlapping E-box ∼1.6 kb upstream of miR-379 (Fig. 3a), suggesting that the identified promoter of the lncRNA-MGC-miRNA cluster may be regulated by CHOP.

### Regulatory role of CHOP

CHOP protein was induced in MMCs treated with TGF-β1, HG or osmotic control mannitol (Fig. 3b), suggesting that diabetic conditions and cellular stress upregulate CHOP, which increases transcription of lnc-MGC and the miRNA cluster. ChIP assays showed significant increase in CHOP occupancy at the CHOP binding sites in the miR-379 promoter in MMCs treated with HG or TGF-β1 (Fig. 3c,d). The upstream (−1.6 kb) region with wild-type (WT) CHOP, or the same region with mutated CHOP binding site (MT) was cloned into the pGL4 luciferase reporter and these plasmids transfected into MMC (Fig. 3e). The WT plasmid showed significantly higher luciferase activity than empty pGL4 whereas CHOP-MT showed significantly reduced promoter activity (Fig. 3e). Together with ChIP data, the CHOP binding site (−1.6 kb) is essential for the expression of lnc-MGC and miR-379 cluster. ChIP of E-box regulators (USF1, Tfe3 and Zeb1) showed increased enrichment of E-box activators (USF1 and Tfe3), but decreased enrichment of the E-box repressor, Zeb1, in MMC treated with TGF-β1 (Supplementary Fig. 21b–d). The E-box binding region might contribute to the regulation of the miR-379 cluster by mechanisms (miRNA-mediated increase of E-box activators and decrease of E-box repressor) similar to those identified in the regulation of collagens in MC under diabetic conditions^16^,^45^.

**Figure 4:**
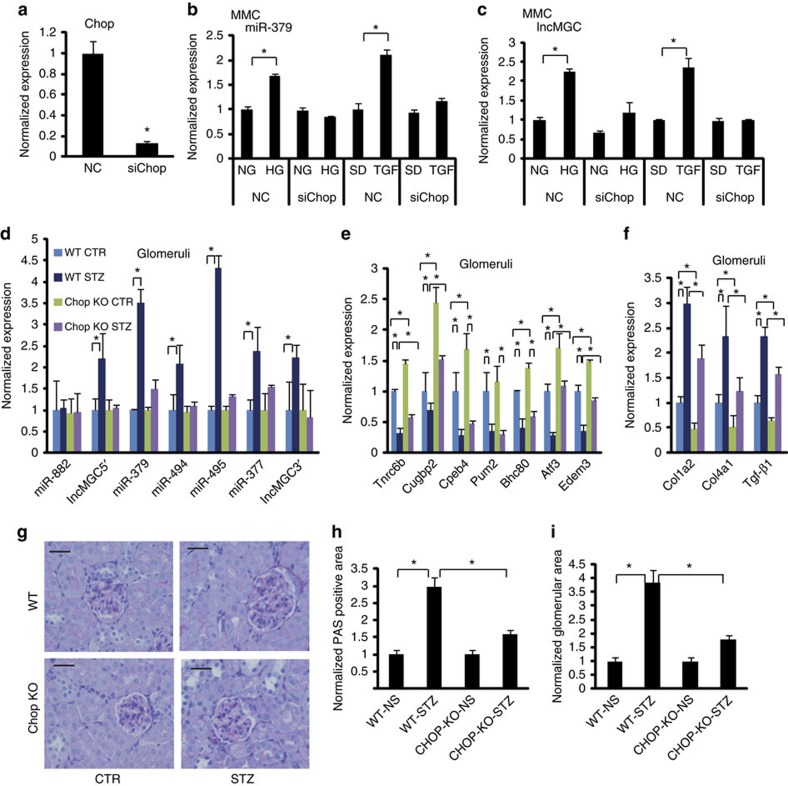
Regulatory role of CHOP (using siRNA and *Chop*-KO mice). (**a**) Effects of *Chop* siRNA. *Chop* expression was significantly decreased by *Chop* siRNA in MMC. (**b**) Effects of *Chop* siRNA on the expression of miR-379. *Chop* siRNA inhibited TGF-β1 or HG mediated increase of miR-379 in MMC but had no effect on basal levels of miR-379. (**c**) Effects of *Chop* siRNA on the expression of lnc-MGC. *Chop* siRNA inhibited the induction of lnc-MGC in MMC treated with TGF-β1 or HG but had no effect on basal levels. Results are mean+s.e. in triplicate PCRs from three independent culture experiments, *P<0.05. (**d**) The miR-379 cluster miRNAs are not induced in the kidneys of diabetic *Chop*-KO mice. The increases of lnc-MGC, miR-379, miR-494, miR-495 and miR-377 noted in glomeruli from diabetic WT mice were not observed in glomeruli from diabetic *Chop*-KO mice (five mice in each group). (**e**) Decrease of potential targets of miR-379 cluster in glomeruli from diabetic WT mice and restoration of their expression in *Chop*-KO mice. (**f**) Increase of profibrotic gene expression in glomeruli from diabetic WT mice was attenuated in *Chop*-KO mice. (**g**–**i**) Glomerular hypertrophy and ECM accumulation were increased in WT diabetic mice and these were attenuated in diabetic *Chop*-KO mice. Scale bar, 20 μm. (**h**) PAS staining in glomeruli from WT and *Chop*-KO mice. PAS positive area (ECM accumulation). (**i**) glomerular area (hypertrophy). *P<0.05.

Figure 4a shows the efficacy of *Chop* short interfering RNA (siRNA). *Chop* siRNA significantly attenuated HG- and TGF-β1-induced expression of miR-379 and lnc-MGC in MMC (Fig. 4b,c). Mir-495 and -377 as well as Mirg levels were also inhibited (Supplementary Fig. 21f–i). These results demonstrate that CHOP is a key transcriptional regulator of the miRNA cluster as well as its host lncRNA-MGC. *Chop* siRNA treatment significantly reversed the downregulation of a miRNA target (*Tnrc6b*) by HG and TGF-β1 in MMC (Supplementary Fig. 22a). *Chop* siRNA also ameliorated TGF-β1-induced expression of profibrotic genes *Col1α2, TGF-β1, Col1α4* and *Pai-1* (Supplementary Fig. 22b–g).

### Cluster miRNAs and lnc-MGC in *Chop-*KO mice

Since our data implicated CHOP as one of the major regulators of lnc-MGC, we examined its *in vivo* functional role using *Chop*-KO mice. Diabetes was induced in *Chop*-KO and control WT mice with STZ injections. *Chop*-KO mice developed diabetes at the same rates as WT. Mice were euthanized four weeks after diabetes induction. Interestingly, diabetes-induced increases in candidate cluster miRNAs as well as lnc-MGC, which were evident in the glomeruli of diabetic WT mice, were abrogated in the glomeruli of diabetic *Chop*-KO mice (Fig. 4d). The levels of key targets of the miR-379 cluster were significantly lower in diabetic mice. However, their levels were significantly higher in *Chop*-KO mice than WT (Fig. 4e). The increased expression of profibrotic genes *Col1α2, Col4α1* and *Tgf-β1* were attenuated in glomeruli from diabetic *Chop*-KO mice compared to diabetic WT mice (Fig. 4f). Histological analysis showed that Periodic acid-Schiff (PAS) staining and glomerular hypertrophy were clearly reduced in diabetic *Chop*-KO mice compared to diabetic WT (Fig. 4g–i).

We cultured MMCs from non-diabetic WT and *Chop*-KO mice and treated them with or without TGF-β1. Both basal and TGF-β1-induced increases in three cluster miRNAs and lnc-MGC (but not miR-882) were ameliorated in MMC derived from *Chop*-KO mice compared to WT mice (Supplementary Fig. 23a). In addition, the expression of candidate target genes of the miR-379 cluster was significantly higher in MMC from *Chop*-KO mice as compared to WT (basal and after TGF-β1 treatment) (Supplementary Fig. 23b). Furthermore, both basal and TGF-β1-induced expression of fibrotic genes, Collagen type I alpha2 (*Col1α2), Col4α1* and *Tgf-β1* (relevant to DN), were significantly attenuated in MMC from the *Chop*-KO mice compared to WT (Supplementary Fig. 23c). TGF-β1-induced increase in cellular hypertrophy was ameliorated in MMCs from *Chop*-KO compared to WT mice (Supplementary Fig. 23d). Similar to the effects seen with TGF-β1, HG-induced increases in lnc-MGC, cluster miRNAs and profibrotic genes, as well as decrease of target genes, were attenuated in MMC from *Chop*-KO mice (Supplementary Fig. 24a–c). When *Chop*-KO MMC were transfected with miR-379 mimic, significant increase of miR-379, profibrotic genes but decrease of targets were observed (Supplementary Fig. 24d–i), confirming that ectopic expression of decreased miRNAs in *Chop*-KO MMC reverses the expression of targets and profibrotic genes.

### Role of ER stress in cluster miRNAs and lnc-MGC expression

To verify the role of ER stress, MMC were treated with tunicamycin (TM), a known ER stress inducer, and the expression of miR-379 cluster miRNAs examined. To optimize conditions for TM treatment, the expression of HSPA5 (heat shock 70 kDa protein 5), also known as GRP78 (glucose-regulated protein, 78 kDa), was examined (Supplementary Fig. 25a). 50 ng ml^−1^ was the minimum dose of TM required to induce significant expression of HSPA5 as well as CHOP (Supplementary Fig. 25a–c). TM (∼50 ng ml^−1^) also upregulated lnc-MGC and key cluster miRNAs in MMC (Supplementary Fig. 25d–i). TM reciprocally decreased several targets of the miR-379 cluster including Edem3, a target of miR-379 (Supplementary Fig. 26a–g). Interestingly, a faster-migrating isoform of Edem3 protein was detected in TM-treated cells but not in MMC treated with TGF-β1 (Supplementary Fig. 26g,h). This is reasonable because TM is an inhibitor of N-glycosylation and the faster-migrating form is an un-glycosylated form of Edem3 that leads to loss of its activity to protect the cells from ER stress^37^. On the other hand, TGF-β1 treatment decreased *Edem3* expression likely through induction of miR-379 since miR-379 mimic oligonucleotides (oligos) reduced *Edem3* levels (Supplementary Fig. 26h,i). This illustrates two independent modes of Edem3 regulation, loss of N-glycosylation and decrease of expression through miR-379 induction (Supplementary Fig. 26g–i). In parallel, profibrotic genes, such as *Col1α2*, *Col4α1*, fibronectin (FN) and *Tgf-β1* were also upregulated by TM (Supplementary Fig. 27a–d). These results suggest that TM (ER stress) increases lnc-MGC and the miR-379 cluster miRNAs, and enhances ER stress and DN phenotypes (hypertrophy and fibrosis) by inhibiting the miR-379 cluster targets.

Increased ER stress has been reported in animal models of DN^46^,^47^,^48^,^49^,^50^,^51^. *XBP1* is spliced and activated by ER stress^52^. We observed increased *Xbp1* splicing in MMC treated with TM, HG or TGF-β1 (Supplementary Fig. 27e–g), suggesting that these factors induce ER stress. In diabetic glomeruli and MMC treated with TGF-β1 or HG, although ATF4 or ATF6 were unaltered, we observed a significant decrease in *Atf3* (Fig. 2e,f), a repressor of CHOP^40^,^41^,^42^ and also a direct target of miR-494 (ref. 43), a miR-379 cluster miRNA confirmed to be increased in diabetic glomeruli. *Xbp1* splicing and *Atf3* downregulation by miR-494 co-operate to upregulate Chop in MMC in response to HG or TGF-β.

### Edem3 is as a target of miR-379

Edem3 protein levels were decreased in MMC treated with TGF-β1, or in MMC transfected with miR-379 mimics (Supplementary Fig. 26h,i). A reporter plasmid encompassing the full 3′UTR of mouse *Edem3* gene (Full) was cotransfected into MMC along with miR-379 mimics (Supplementary Fig. 28a,b). TGF-β1 treatment reduced the luciferase activity of this reporter compared to SD control. miR-379 mimics also inhibited the luciferase activity of this reporter compared to negative control mimics (CTR), suggesting *Edem3* is a direct target of miR-379. Interestingly, a target site of miR-200b/c was also found in the 3′UTR of *Edem3* and miR-200b mimic oligos inhibited the reporter activity (Supplementary Fig. 28a,b). Because miR-200b/c are upregulated in glomeruli from diabetic mice and in MMC treated with TGF-β1^2^,^6^,^16^, miR-200 family and miR-379 cluster may collaborate to inhibit *Edem3* expression. miR-379 and miR-200b mimics had no effect on reporters containing partial deletion of the region harbouring miR-379 and miR-200b/c target sites in the 3′UTR of *Edem3*, verifying these sites to be *bonafide* targets of miR-379 and miR-200b/c (Supplementary Fig. 28c). These results also suggest that one of the mechanisms by which the miR-379 cluster upregulated in diabetes contributes to DN is through ER stress promoted by loss of Edem3.

### Inhibition of lnc-MGC by GapmeRs *in vitro* and *in vivo*

siRNAs designed to target lnc-MGC regions located upstream of miR-379 were effective to silence lnc-MGC and cluster miRNAs (Supplementary Fig. 29a,b). However, since this approach needed transfection with three siRNAs and not practical for *in vivo* targeting, we evaluated ‘GapmeRs’ to inhibit lnc-MGC expression.

Locked nucleic acid (LNA)-modified anti-miRNAs are useful to target specific miRNAs to protect diabetic mice from various features of DN^12^. LNA modification has several advantages, including reduced toxicity, lower dosing and efficient targeting. LNA GapmeRs are antisense oligos optimized for specific inhibition of RNA *in vitro* and *in vivo*^53^,^54^. GapmeRs induce RNase H-dependent RNA degradation in the nucleus, and hence nuclear retained RNAs are effectively targeted. To knockdown lnc-MGC, four LNA-modified GapmeR oligos (MGC1, MGC5, MGC8 and MGC10) were designed (Supplementary Fig. 30a) having three LNAs at both 5’ and 3’ ends and ten DNAs in the centre, and a fully phosphorothioated backbone (Supplementary Fig. 30b). MMC were transfected with these GapmeRs or a control LNA GapmeR and the expression of lnc-MGC examined. The control LNA GapmeR oligo had the same modification as our lncRNA targeting GapmeR but with no homology to any known mRNA, miRNA, or lncRNA in mouse, rat and human. MGC10 consistently inhibited lnc-MGC expression significantly at 48 h after transfection in two independent experiments. Of the other three GapmeRs, MGC1 and MGC5 showed some inhibition, but were less effective than MGC10 and not consistent in their actions (Supplementary Fig. 30c,d). MGC10 also reduced the expression of lnc-MGC under TGF-β1 treated conditions in MMC (Supplementary Fig. 30e). In addition, key miRNAs in the miR-379 megacluster were downregulated by MGC10 (Supplementary Fig. 31a–c). Furthermore, targets (*Edem3, Tnrc6b* and *Bhc80*) of the miR-379 cluster were upregulated by MGC10 (Supplementary Fig. 31d–g).

Because MGC10 was effective in reducing the expression of lnc-MGC and key miR-379 cluster miRNAs in MMC *in vitro*, we next tested its actions in mouse kidneys *in vivo* (Supplementary Fig. 32). Subcutaneous injection of 5 mg kg^−1^ MGC10 into normal mice significantly reduced the expression of lnc-MGC in their kidneys (by 24 ∼72 h) (Supplementary Fig. 32a,b). miR-379, -495 and -377 (but not miR-882), were also reduced in the same samples (Supplementary Fig. 32c–f).

**Figure 5:**
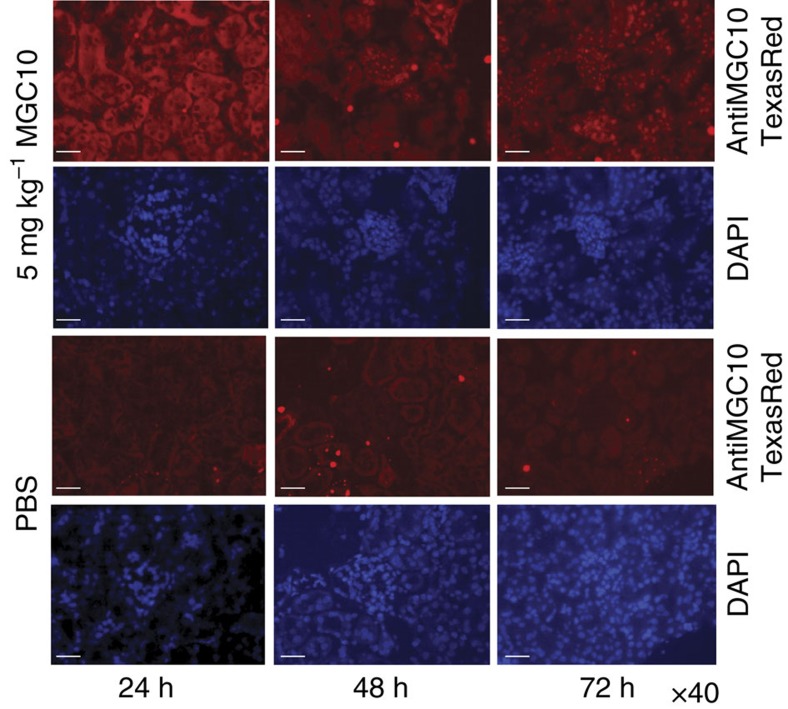
Detection of Gapmer MGC10 in mouse kidney by *in situ* hybridization. 24 h after subcutaneous injection (5 mg kg^−1^) of MGC10, red fluorescence signals (by *in situ* hybridization of renal sections with a Texas Red conjugated complementary probe) were detected in tubular and glomerular areas (mostly in cytoplasm). However, after 48 h, nuclear accumulation of MGC10 was observed and the signals were more enriched in nuclei at 72 h after injection. Blue 4,6-diamidino-2-phenylindole signals represent nuclear regions. Weak background was detected in the kidneys of mice injected with vehicle (PBS) as control. Scale bar, 20 μm.

To confirm the renal delivery of MGC10, a fluorescent antisense LNA-modified probe was designed for in situ hybridization. Interestingly, by 24 h there was widespread signal in cytosolic regions of both glomerular and tubular regions in kidneys of mice injected with MGC10, while clear nuclear accumulation of MGC10 was observed in these kidney compartments at 48–72 h after the injection (Fig. 5). Only weak background signal was detected in the kidneys of vehicle (PBS) injected mice. A recent report suggested that phosphorothioated oligonucleotides are transported into the nucleus by a protein (TCP1) complex^55^. Because MGC10 is fully phosphorothioated, it is efficiently transported into the nucleus where it cleaves lnc-MGC RNA and thereby suppresses the expression of the miR-379 cluster (Supplementary Fig. 33).

Next, MGC10 was tested in diabetic mice. Five non-diabetic mice (NS), five un-injected diabetic mice (STZ), six diabetic mice injected with negative control GapmeR oligonucleotides (STZ+C) and six diabetic mice injected with 5 mg kg^−1^ MGC10 targeting lnc-MGC (STZ+MGC10) were examined at 5 weeks after the onset of diabetes (Supplementary Fig. 34a). Serum profiling showed significant increase in glucose levels in all diabetic mice (STZ, STZ+C and STZ+MGC10) compared to non-diabetic mice (NS). There was no significant difference in parameters of liver and kidney toxicity or inflammation (inflammatory cytokines) due to MGC10 injection, suggesting that MGC10 does not alter glucose levels, and does not induce significant toxicity or inflammation (Supplementary Figs 34b and 35a,b).

**Figure 6:**
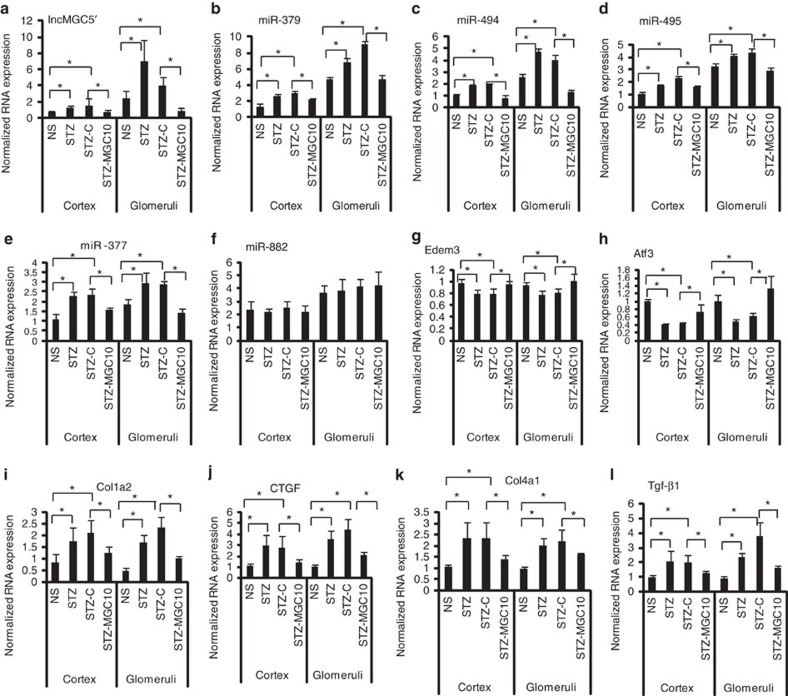
*In vivo* effects of GapmeR targeting lnc-MGC (MGC10) in mice. (**a**) Increase of lnc-MGC in kidney cortex and glomeruli from STZ diabetic mice and its significant inhibition by MGC10 injection. Five mice for NS and STZ groups and six mice for STZ+C and STZ-MGC10 groups. Results are mean+s.e. in triplicate PCRs from each mouse. *P<0.05. (**b**–**f**) Increase of miR-379 (**b**), miR-494 (**c**), miR-495 (**d**), miR-377 (**e**) but not miR-822 (**f**) in kidney cortex and glomeruli from STZ diabetic mice and their significant inhibition by injection of MGC10. (**g**,**h**) Decrease of miR-379 target *Edem3* and miR-494 target *Atf3* in kidney cortex and glomeruli from STZ diabetic mice and significant restoration of their levels by injection with MGC10. (**i**–**l**) Increase of profibrotic genes *Col1a2* (**i**), *Ctgf* (**j**), *Col4a1* (**k**) and *Tgf-β1* (**l**) in kidney cortex and glomeruli from STZ diabetic mice and significant inhibition by injection of MGC10. *P<0.05.

Lnc-MGC expression was higher in the kidneys (cortex and glomeruli) from diabetic versus non-diabetic mice. Its expression was significantly reduced in kidneys of diabetic mice injected with MGC10 (STZ-MGC10) compared to control GapmeR (STZ-C), demonstrating that MGC10 is effective even in diabetic mice (Fig. 6a and Supplementary Fig. 36a–c). Expression of the cluster miRNAs, (miR-379, -494, -495, -377) but not miR-882, and Mirg depicted a similar pattern as lnc-MGC, demonstrating specificity to this cluster (Fig. 6a–f). Key targets (*Edem3, Atf3, Tnrc6B, Cpeb4, Pum2*) of the miR-379 cluster were reduced in kidneys from diabetic mice but their expression was restored in the kidney cortex and glomeruli of diabetic mice injected with MGC10 (Fig. 6g,h and Supplementary Fig. 36d–f). Profibrotic genes, *Tgf-β1, Col1a2, Col4a1, Ctgf*, which were upregulated in the kidneys of diabetic mice, were reduced in diabetic mice injected with MGC10 (Fig. 6i–l).

**Figure 7:**
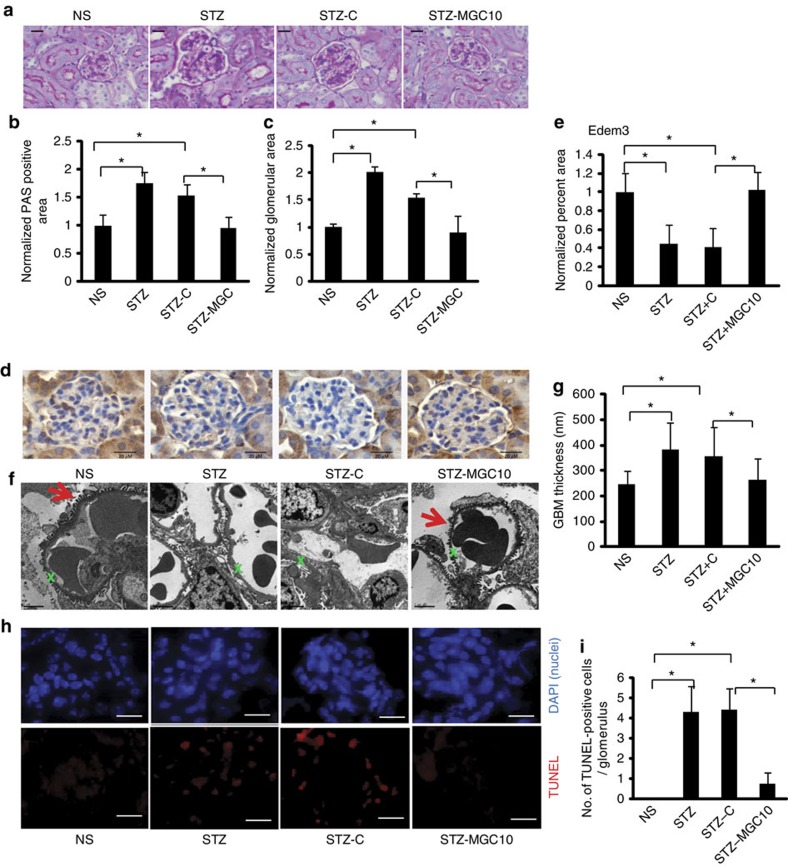
*In vivo* effects of MGC10 on kidney pathology in diabetic mice. (**a**) PAS staining of kidney sections. Scale bar, 20 μm. (**b**,**c**) PAS positive area (**b**) and glomerular area (**c**) were significantly increased in diabetic mice (STZ and STZ-C) compared with non-diabetic mice (NS), and these were attenuated in diabetic mice injected with Gapmer targeting lnc-MGC, MGC10 (STZ-MGC10). (**d**) Edem3 staining in kidney sections. Scale bar, 20 μm. (**e**) Edem3 staining in kidney glomeruli was significantly decreased in diabetic mice (STZ and STZ+C) compared with non-diabetic mice (NS), and this was reversed in diabetic mice injected with MGC10 (STZ+MGC10). *P<0.05. (**f**) EM structures of glomeruli. Distorted podocyte structure and increased GBM thickness were observed in glomeruli from diabetic mice as well as in diabetic mice injected with negative control GapmeR (STZ and STZ+C), but these features were attenuated in diabetic mice injected by GapmeR targeting lnc-MGC (STZ+MGC10). Red arrows denote intact podocytes. Green Xs denote a section of GBM. Scale bar, 2 μm. (**g**) GBM thickness was significantly increased in diabetic mice (STZ and STZ-C) compared with non-diabetic mice (NS), and this was attenuated in diabetic mice injected with MGC10 (STZ-MGC10). *P<0.05. (**h**) TUNEL assay. Top panels, 4,6-diamidino-2-phenylindole staining (blue) showing nuclei; second, TUNEL (red). × 400 magnification. (**i**) Significant increase of TUNEL-positive cells was observed in diabetic mice (STZ and STZ-C) compared with non-diabetic mice (NS), and this was attenuated in diabetic mice injected with MGC10 (STZ-MGC10). *P<0.05. Scale bar, 20 μm.

PAS staining of renal sections showed mesangial expansion and increased glomerular size in diabetic mice compared to that in non-diabetic mice and these features were reduced in diabetic mice injected with MGC10 (Fig. 7a–c). With respect to ER stress, immunostaining of Edem3, a target of miR-379, showed significant decrease in glomeruli of diabetic mice and this was reversed in glomeruli of diabetic mice injected with MGC10 (Fig. 7d,e).

TGF-β or diabetic conditions (as well as ER stress) induce glomerular podocyte dysfunction and death^56^. In order to determine whether MGC10 confers any protection on podocytes in diabetes, we assessed podocyte effacement and glomerular basement membrane (GBM) thickness using electron microscopy (EM; Fig. 7f,g). Clear protection from diabetes-induced podocyte effacement and GBM thickening was observed in diabetic mice treated with the MGC10 compared to control oligo. Cell death measured by Tunel assay was increased in glomeruli of diabetic mice compared to non-diabetic mice, which was attenuated by MGC10 (Fig. 7h,i). These results suggest that MGC10 is effective in reducing the expression of not only lnc-MGC and miR-379 cluster miRNAs *in vivo* in diabetic mice, but also restores the expression of the cluster miRNA targets, inhibits profibrotic genes and prevents glomerular fibrosis, podocyte death, and hypertrophy in diabetic mice.

### Human homologue of lnc-MGC, and inhibition by GapmeR

**Figure 8:**
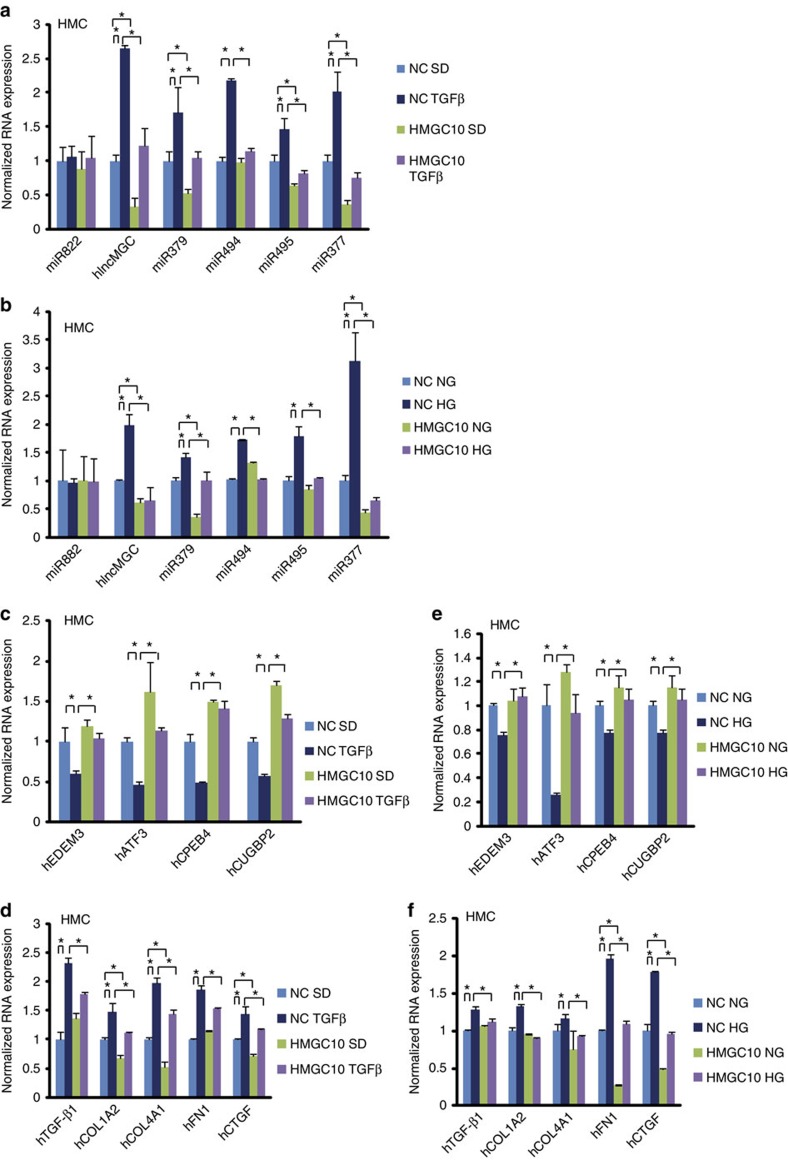
Human homologue of lnc-MGC and its inhibition by HMGC10 in HMC. Significant increase of hlnc-MGC (human homologue of lnc-MGC), miR-379, miR-494, miR-495 and miR-377 in human MC (HMC) treated with TGF-β1 (**a**) or HG (**b**) compared with respective controls (SD or normal glucose), but not miR-882 (outside of miR-379 cluster). These increases were significantly reduced in HMC transfected with HMGC10 compared with control oligo. (**c**) HMGC10 mediated restoration of miR-379 cluster targets, *EDEM3, ATF3, CUGBP2* and *CPEB4* which were inhibited by TGF-β1 in HMC. (**d**) Significant increase of profibrotic genes, *TGF-β1, COL1A2, COL4A1, FN1* and *CTGF* in HMC treated with TGF-β1 and their significant inhibition in HMC transfected with HMGC10. (**e**) HMGC10 mediated restoration of miR-379 cluster targets, *EDEM3, ATF3, CUGBP2* and *CPEB4* which were inhibited by HG in HMC. (**f**) Significant increase of profibrotic genes, *TGF-β1, COL1A2, COL4A1, FN1* and *CTGF* in HMC treated with HG which was significantly inhibited by HMGC10. Results are mean+s.e. in triplicate PCRs from three independent culture experiments. *P<0.05.

To test if the miR-379 cluster is similarly regulated in human cells, human MC (HMC) were treated with TGF-β1 or HG. The human homologue of lnc-MGC (hlnc-MGC) was examined by PCR using human specific primers (Supplementary Fig. 37a). hlnc-MGC and miR-379 cluster miRNAs were increased by TGF-β1 or HG in HMC, with miR-882 showing no significant difference (Fig. 8a,b). Since the target sequence in human hlnc-MGC has two-base mismatches, we designed a human version of GapmeR MGC10 (HMGC10) based on the human sequence (Supplementary Fig. 37a,b). Basic GapmeR chemistry was similar to the mouse MGC10. Transfection conditions in HMC were optimized for efficacy and cell viability (Supplementary Fig. 37c). hlnc-MGC levels were significantly reduced by HMGC10 in HMC compared to negative control (Supplementary Fig. 37d). Key targets, EDEM3 and CPEB4, were also decreased by TGF-β1 in HMC (Supplementary Fig. 37e–g).

Human lnc-MGC expression was significantly inhibited by HMGC10 under both basal and TGF-β1 treated conditions (Fig. 8a). Similar trends were observed for miR-379, miR-494, miR-495 and miR-377, but not miR-822, suggesting that inhibition of hlnc-MGC (host RNA) by HMGC10 reduces the cluster miRNAs in HMC treated with TGF-β1 (Fig. 8a). Similarly, HG-induced expression of hlnc-MGC, and cluster miRNAs in HMC were significantly inhibited by HMGC10 (Fig. 8b). The reduction of candidate targets (*EDEM3, ATF3, CPEB4* and *CUGBP2*) of the cluster miRNAs by TGF-β1 was reversed by HMGC10 in HMC (Fig. 8c). Induction of profibrotic genes (*TGF-β1, COL1A2, COL4A1, FN1* and *CTGF*) by TGF-β1 was attenuated by HMGC10 (Fig. 8d). HG-induced reduction of target genes (*EDEM3, ATF3, CPEB4* and *CUGBP2*) was reversed by HMGC10 (Fig. 8e). HG-induced increased expression of profibrotic genes (*TGF- β1, COL1A2, COL4A1, FN1* and *CTGF*) was also ameliorated by HMGC10 in HMC (Fig. 8f). These results suggest that reduction of hlnc-MGC by HMGC10 can suppress the miR-379 cluster miRNAs, restore the expression of their targets and inhibit the profibrotic genes in HMC treated with TGF-β1 or HG, highlighting the potential translational significance.

### Expression of miR-379 cluster miRNAs in human kidney tissue

We further examined if these cluster miRNAs are expressed in glomeruli of patients with diabetic kidney disease^17^. Several cluster miRNAs were examined by quantitative PCR with reverse transcription (qRT-PCR) and small RNA sequencing in RNA isolated from microdissected glomeruli of kidney biopsies from 46 Southwestern American (Pima) Indians with documented type-2 diabetes^57^, as described^17^,^58^. The cluster miRNAs were expressed robustly in these diabetic patient samples with read frequency comparable to miR-192, which is highly enriched in the kidney and implicated in DN^17^,^58^ (Supplementary Table 1).

DN in humans is associated with glomerular hypertrophy, mesangial expansion and loss of podocytes leading to glomerulosclerosis^2^,^6^,^59^. We further found that increased expression of the precursors of some of the cluster miRNAs is significantly associated with morphometric parameters of increased glomerular damage (decreased podocyte density and increased podocyte and glomerular volume as well as mesangial index) in microdissected glomeruli of human nephrectomy tissues that showed various stages of glomerular pathology similar to early stages diabetic glomerulopathy^60^ (Supplementary Table 2). These associations suggest involvement of the megacluster miRNAs in human glomerular diseases including DN, although additional studies are necessary.

## Discussion

**Figure 9:**
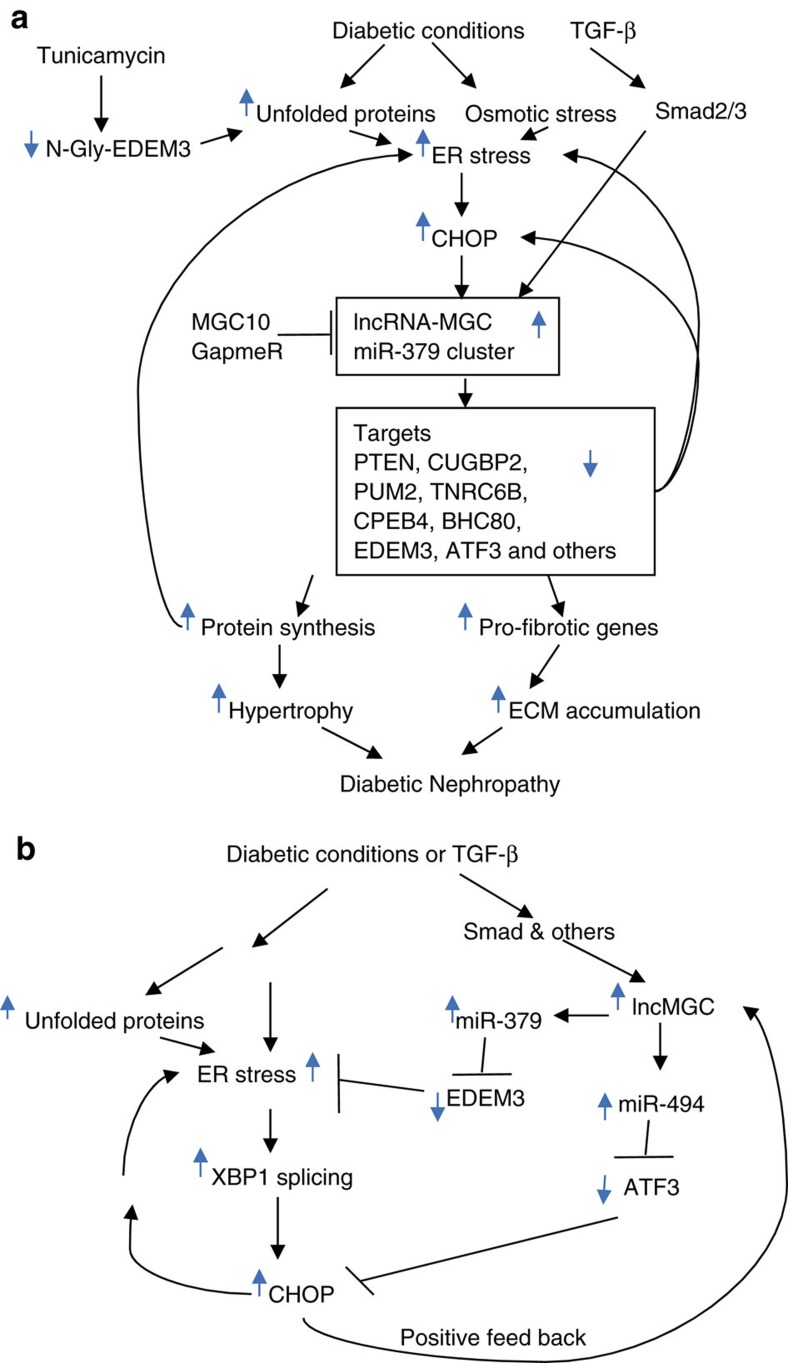
ER-stress-regulated lnc-MGC hosting a miRNA cluster mediates features of DN. (**a**) Proposed model for the initiation of the early stages of DN through lnc-MGC, the miR-379 megacluster and related ER stress. Diabetic conditions and ensuing increases in TGF-β1 can promote ER stress and induce lnc-MGC via Smad2/3 and CHOP. This results in increased expression of nearly 40 megacluster component miRNAs and subsequent downregulation of numerous targets whose functions are related to hypertrophy, ECM production and fibrosis associated with early features of DN. Targeting lnc-MGC *in vivo* with specific LNA GapmeRs can attenuate these pathological events, and thereby prevent further disease progression. Please see main text for additional details. (**b**) A schematic model of CHOP upregulation through two pathways: first, XBP-1 splicing activated by ER stress; and second, reduction in levels of ATF3 (a repressor of CHOP) due to targeting by the cluster miR-494 in MC treated with TM, HG or TGF-β1. CHOP in turn can induce lnc-MGC and ER stress in positive feedback loops.

Our data suggest that diabetic conditions (HG), which also induce TGF-β1, upregulates ∼40 miRNAs within the miR-379 megacluster that target regulators of ER stress and protein synthesis resulting in hypertrophy and fibrosis related to DN (Fig. 9a). The host transcript (lnc-MGC) of this megacluster is also induced in parallel by Smad and CHOP, an ER stress-responsive transcription factor. miR-379 cluster expression depends on the expression of the host lnc-MGC from its promoter. siRNAs targeting *Chop* inhibited the induction of lnc-MGC and miR-379 cluster miRNAs as well as parameters (ECM gene expression and cellular hypertrophy) of DN *in vitro*. miRNAs in this cluster target several groups of genes, transcription factors, RNA binding proteins regulating fibrotic genes, as well as protein synthesis and ER stress, which result in hypertrophy via increased protein synthesis and fibrosis (ECM accumulation). Moreover, several cluster miRNAs had binding sites in the 3′UTRs of multiple similar target genes. Although each cluster miRNA may have relatively modest effects by itself, the cumulative effects of upregulation of nearly 40 cluster miRNAs under diabetic conditions can have a considerable and synergistic impact to augment renal hypertrophy, fibrosis and dysfunction.

Induction of these miRNAs as well as features of early DN were ameliorated in the kidneys of diabetic *Chop*-KO mice compared to diabetic WT mice. Similarly, induction of these miRNAs and profibrotic genes by TGF-β1 and HG were attenuated in MMC from *Chop*-KO mice. An ER stress inducer TM also induced lnc-MGC and cluster miRNAs in MMC likely through reduction of N-glycosylation of EDEM3. EDEM3 is reduced in pancreatic beta cells in type-2 diabetes^61^. The Nephroseq database (http://www.nephroseq.org/) revealed correlations between GFR and EDEM3 expression in the normal human population, as well as in patients with DN, IgA nephropathy, and hypertensive nephropathy. Correlations of EDEM3 expression with obesity in human and mouse were also found in Nephroseq.

ER stress has been observed in patients with progressive DN^62^. Increased ER stress, renal Chop expression and albuminuria were reported in aged diabetic mice, and albuminuria was attenuated in diabetic *Chop*-KO mice compared to WT^48^. Reports showed increased levels of ER stress in animal models of DN^46^,^47^,^48^,^49^,^50^,^51^,^63^,^64^ and also amelioration of DN by ER stress inhibitors like 4-phenyl butyric acid^46^ and the chemical chaperone TUDCA^47^. In the current study, we observed increase in *Xbp1* splicing which upregulates Chop (Supplementary Fig. 27e–g), but decreases in *Atf3*, a repressor of *Chop* as well as a direct target of miR-494 (refs 40, 41, 42, 43) (an upregulated cluster miRNA) in MMC treated with HG or TGF-β1 or in glomeruli from diabetic mice. The Nephroseq database also indicated lower levels of ATF3 in patients with DN compared to healthy controls. These data suggest two important ER stress-related signalling events, *Xbp1* splicing and *Atf3* downregulation by miR-494, which co-operate to upregulate Chop in response to HG or TGF-β1 in MMC (Fig. 9b). Therefore, ER stress is a major inducer of lnc-MGC in diabetes. Of course, CHOP can regulate other genes related to DN besides lnc-MGC, and also have cell-specific effects in the kidney^64^.

We further demonstrated that inhibition of the cluster miRNAs by a GapmeR (MGC10) which effectively targeted lnc-MGC, ameliorated key features of early DN (ECM accumulation and glomerular hypertrophy) in a mouse model. Since this lncRNA-miRNA cluster is induced early in the disease process and controls several critical mechanisms associated with the initial stages of DN, reducing its expression can prevent further progression of DN. In early DN, MC are a major source of increased TGF-β1 production^65^. HG induces TGF- β1 in MC through promoter E-boxes^16^,^66^. However, HG induces only TGF-β RII (receptor) but not TGF-β1 in podocytes^67^. TGF-β1 secreted by MC may have autocrine effects which activate MC, and paracrine effects which activate other cells including podocytes. This suggests that events occurring in MC (induction of TGF-β1) are earlier than those in podocytes (podocyte loss induced by diabetes and TGF-β1). Significant podocyte loss was detected at 20 weeks after the onset of diabetes in mice, while podocyte injury, effacement and death were detected at 4 weeks which can be related to subsequent proteinuria^56^. However, induction of TGF-β1 and ECM accumulation in MC were detected by 2 weeks after the onset of diabetes^12^,^17^. Therefore, MC injury and ECM accumulation are most likely initial events in glomeruli, with increased TGF-β1 secreted by MC triggering subsequent podocyte injury/death soon after^65^. Furthermore, other factors related to DN (HG, advanced glycation end-products, lipids, Angiotensin II) can also promote ER stress and subsequently induce lnc-MGC via TGF-β1-dependent and independent pathways. Therefore, strategies, such as those described here, targeting early events in the glomeruli (MC and podocyte injury) can prevent DN progression. This is supported by our data showing GapmeR MGC10 attenuates not only the increases in glomerular mesangial hypertrophy/expansion, fibrosis and GBM thickening in diabetic mice at 4 weeks, but also the increases in podocyte effacement and cell death.

Notably, key cluster miRNAs as well as hlnc-MGC were also upregulated in HMC treated with HG or TGF- β1 and the corresponding targets were downregulated. A GapmeR targeting hlnc-MGC ameliorated these events, as well as the induction of fibrotic genes. Most of the cluster miRNAs were expressed in microdissected glomeruli obtained from diabetic Pima Indians, and the expression of some of their precursors were increased with glomerular damage, suggesting relevance for human glomerular diseases.

Together, these results demonstrate new functions for a lncRNA and its component miRNAs in DN and possibly other glomerular diseases. Several reports have demonstrated that inhibition of specific miRNAs by modified oligonucleotides ameliorated DN in animal models^6^,^10^,^11^,^12^,^16^,^17^,^18^,^19^,^20^,^21^. However, to our knowledge, this is the first report that a single oligonucleotide (GapmeR) targeting a lncRNA which hosts a cluster of miRNAs can control the expression of the host lncRNA, component miRNAs, their targets, profibrotic genes and symptoms of DN in mice, and in human MC.

## Methods

### Mouse models of diabetes

All animal studies were conducted according to a protocol approved by the Institutional Animal Care and Use Committee at the Beckman Research Institute of City of Hope. Male type-2 diabetic *db/db* mice (T2D leptin receptor deficient; Strain BKS.Cg-m+/+lepr db/J) and genetic control non-diabetic *db/*+ mice (10–12 weeks old), were obtained from The Jackson Laboratory (Bar Harbor, ME)^11^,^17^. Male C57BL/6 mice (10 week old, The Jackson Laboratory) were injected with 50 mg kg^−1^ of STZ intraperitoneally on 5 consecutive days. Mice injected with diluent served as controls. Diabetes was confirmed by tail vein blood glucose levels (fasting glucose >300 mg dl^−1^). Each group was composed of five to six mice. Mice were sacrificed at 4–5 or 22 (ref. 17) weeks post-induction of diabetes. Glomeruli were isolated from freshly harvested kidneys by a sieving technique^11^,^17^ in which renal capsules were removed, and the cortical tissue of each kidney separated by dissection. The cortical tissue was then carefully strained through a stainless sieve with a pore size of 150 μm by applying gentle pressure. Enriched glomerular tissue below the sieve was collected and transferred to another sieve with a pore size of 75 μm. After several washes with cold PBS, the glomerular tissue remaining on top of the sieve was collected. Pooled glomeruli were centrifuged, and the pellet was collected for RNA, protein extraction or for preparing MMCs^11^,^17^. Male *Chop*-KO mice were also obtained from the Jackson Laboratory (B6.129S(Cg)-*Ddit3*^*tm2.1Dron*^/J). Based on our previous experience, sample size was determined to have enough power to detect an estimated difference between two groups. With minimum sample size of 5 in each group, the study can provide at least 80% power to detect an effect size of 2 between diabetic and non-diabetic groups or treated and untreated groups at the 0.05 significant level using two-sided *t*-test. Since we expected larger variation between groups especially for the mice with oligo-injection, we used more than 5 mice in each group (with 6 mice in each group, we have 80% power to detect an effect size of 1.8 at the 0.05 confidence level). Our actual results with current sample size did show statistical significance for majority of the miRNAs in the cluster. Histopathological and biochemical analysis of tissues or cells derived from animal models were performed by investigators masked to the genotypes or treatments of the animals.

### Primary mesangial cells

MMC were obtained from glomeruli and cultured *in vitro*^11^,^28^. Glomeruli isolated from freshly harvested kidneys were incubated with 0.1 mg ml^−1^ collagenase at 37 °C in shaking water bath for 15 min. After incubation with shaking, cells were cultured in Roswell Park Memorial Institute medium supplemented with 20% foetal bovine serum and 20 μg ml^−1^ Insulin. After 2–3 passages, MMC were maintained in Roswell Park Memorial Institute medium 1640 medium supplemented with 10% foetal bovine serum. Passages 5–7 were used for experiments.

Human MCs (CC-2559, 47 Y Male) were purchased from Lonza Walkersville, Inc. and cultured as per the instructions of the provider.

### Small RNA sequencing

Total RNA was used for the construction of small RNA libraries, cluster generation and then deep sequencing (at the Beckman Research Institute Integrative Genomics Core) using the Alternative v1.5 Protocol (Illumina Inc., San Diego, CA) with minor optimization as described previously^22^. Briefly, 0.5 μg of total RNA was ligated to sRNA 3′adaptor (5′-AUCUCGUAUGCCGUCUUCUGCUUG-3′) using T4 RNA Ligase 2, truncated (New England BioLabs, Ipswich, MA) at 22 °C for 1 h, and subsequently ligated to the SRA 5′adaptor (5′-GUUCAGAGUUCUACAGUCCGACGAUC-3′) with T4 RNA ligase (New England BioLabs, Ipswich, MA) at 20 °C for 1 h. The adaptor-linked RNA was converted to single-stranded cDNA using Superscript II reverse transcriptase (Invitrogen) and RT-Primer (5′-CAAGCAGAAGACGGCATACGA-3′) and then amplified with Phusion DNA Polymerase (Finnzymes Thermo Scientific, Pittsburgh, PA) for 12 cycles using primers (5′-CAAGCAGAAGACGGCATACGA-3′; 5′-AATGATACGGCGACCACCGACAGGTTCAGAGTTCTACAGTCCGA-3′). PAGE purification was carried out to select small RNAs of 17–52 nucleotides in length. The purified library was quantified using qPCR, used for cluster generation on cBot, and sequenced using Genome Analyzer IIx (Illumina).

### Analysis of small RNA sequencing data

For analyses of small RNA sequencing data^22^, raw data in FASTQ format generated from the Illumina pipeline was aligned against UCSC mouse assembly mm9 using Novoalign software. The 3′-adaptor sequence of the raw reads was first trimmed by Novoalign, and reads with 16 or more bases were aligned to the genome. For reads aligned to multiple locations on the genome, one aligned region was randomly selected for counting the number of reads as described^22^. Genomic locus of each mouse mature miRNA was generated by aligning mouse mature miRNA sequences (miRBase, www.mirbase.org) without allowing mismatches. For each sample, the reads corresponding to the mature miRNA genomic loci (including 10-base flanking regions) were counted to obtain expression levels of total miRNAs. The resulting miRNA expression data set was further normalized by scaling the total mature miRNA counts in each sample to 6 million. miRNAs with scaled reads of no <5 in either sample were considered as detectable ones. Among them, differentially expressed miRNAs were identified with at least twofold change in glomeruli from STZ type1 diabetic mice versus non-diabetic mice. Pre-ranked GSEA (ref. 24) were conducted on miR-379 cluster miRNAs using all the detectable miRNAs ranked by log2 fold changes between the two samples. For each miR-379 cluster miRNA, the potential human or mouse target genes containing at least 3 conserved binding sites were identified using the online bioinformatics tool TargetScan (http://www.targetscan.org/). The human/mouse target genes were then pooled together to generate human/mouse gene set. IPA was applied to each gene set for gene ontology, pathway and network analyses.

### miRNAs and siRNAs

Oligonucleotide mimics of miRNAs, siRNAs and corresponding control oligos were obtained from integrated DNA technologies or Thermo Fisher Scientific Inc. (Waltham, MA), as described^11^,^28^. Wild-type (WT) MMCs (from WT C57BL/6 mice), MMC transfected with *Chop* siRNA, and MMCs from *Chop*-KO mice were treated with HG, mannitol and TGF-β1 as described^11^,^28^. Briefly, cells (∼10^6^/transfection) were transfected with siRNA or miRNA oligonucleotides using an Amaxa Nucleofector (Lonza, Basel, Switzerland) according to the manufacturer’s protocols^11^,^28^. siRNAs (double-stranded oligos of three pairs of sense (S) and antisense (AS) synthesized oligos, S1 rCrArUrCrUrGrCrUrUrCrCrCrArCrUrGrCrCrArArArUrCAG and AS1 rCrUrGrArUrUrUrGrGrCrArGrUrGrGrGrArArGrCrArGrArUrGrUrG; S2 rUrCrArGrCrArCrCrGrUrGrCrArArCrCrArUrUrCrArArGGA and AS2 rUrCrCrUrUrGrArArUrGrGrUrUrGrCrArCrGrGrUrGrCrUrGrArArA; S3 rCrUrUrCrArUrCrUrGrGrUrArArUrGrUrArCrUrArCrCrUGA and AS3 rUrCrArGrGrUrArGrUrArCrArUrUrArCrCrArGrArUrGrArArGrGrC; (r, ribose) against mouse upstream region of miR-379 were obtained from integrated DNA technologies. Non-targeting siRNA controls were obtained from Thermo Fisher Scientific Inc. MMC were trypsinized and resuspended in basic nucleofection solution at 1 × 10^7^ ml^−1^. Subsequently, 100 μl of cell suspension (1 × 10^6^ cells) was mixed with miRNA mimic, hairpin inhibitor oligonucleotides, or ON-TARGET plus siRNA or negative controls (Thermo Fischer Scientific Inc., Waltham, MA). Transfected cells were harvested for RNA and protein extraction. RNA was extracted from the cells and the expression of lncRNA-MGC, and miRNAs within the cluster were examined using primers designed for each of the mature miRNAs (miRBase). MMCs were transfected with oligonucleotide mimics, siRNAs of candidate miRNAs or negative control (NC) oligos to determine if manipulating their levels can influence TGF-β1 and HG responses. At 48–72 h post-transfection, the expression of miRNA target genes, fibrosis and hypertrophy related genes, and proteins induced by TGF-β1/HG were determined by RT-qPCR and western blotting using our published methods^11^,^45^.

### Real-time qPCR

Real-time qRT-PCR analysis was performed as previously described^11^,^45^. Briefly, RNA was extracted using miRNeasy columns (Qiagen, Valencia, CA). miRNA expression analysis was performed using the qScript miRNA cDNA synthesis kit (Quanta Biosciences, Gaithersburg, MD) and PerfeCTa SYBR Green Supermix (Quanta Biosciences). miRNAs were amplified using specific mature miRNA sequences as forward primers and the universal primer provided in the kit as the reverse primer. U6 was used as internal control. A GeneAmp RNA PCR kit (Applied Biosystems, Carlsbad, CA) and POWER SYBR Green mix (Applied Biosystems) were used for mRNA quantification. PCR primer sequences are in Supplementary Table 3.

### Western blotting

Immunoblotting was performed as described previously^11^,^28^. Cells were lysed in Laemmli’s sample buffer. Lysates were fractionated on 10% SDS-polyacrylamide gels (Bio-Rad) and transferred to nitrocellulose membrane. Membranes were immunoblotted with appropriate antibodies. Blots were scanned using GS-800 densitometer and quantified with Quantity One software (Bio-Rad). Wider (uncropped) scans of blots are shown in Supplementary Figs 38 and 39. Antibodies used were β-actin (Sigma, A-5441), CHOP (Santa Cruz Biotechnology, sc-793), ZEB1(Santa Cruz Biotechnology, sc-25388), USF1(Santa Cruz Biotechnology, sc-229), EDEM3 (Abcam, ab67108), BHC60 (Santa Cruz Biotechnology, sc-109846), CPEB4 (abcam, ab83009), Tfe3 (BD Bioscience, cat# 554263) and CUGBP2 (GenWay Biotech, Inc., cat# 18-003-43569). Antibodies were used at 1:1,000 dilution.

### Rapid amplification of 5′ complementary DNA ends (5′ RACE)

We used 5′ RACE to identify the putative upstream promoter region of the miR-379 megacluster and host lncRNA-MGC. 5′ RACE was performed using GeneRacer kit (Invitrogen) using RACE primers, miR379upR1, 5′-GACCCCTAGAAACTTGGGCCTTGTCCCTG-3′ and miR379upR2, 5′-TCAGGAACCATGGAACGGTGTTGACCCCTAG-3′. Amplified PCR fragments were cloned and sequenced and the transcription start site determined using RNAs from glomeruli from diabetic mice (STZ). To amplify the upstream region, primer miR379upINR including potential initiator (INR), 5′-ATTTTTCTGAGTTAGTGTGGCCTTCATCTG-3′ was used.

### Reporter assays

Mouse *Edem3* 3′UTR reporter plasmid was purchased from OriGene (Rockville, MD). Deletion mutants of *Edem3* 3′UTR were generated by digesting full-length 3′UTR with EcoRI and re-ligation. Full-length and deletion mutants of reporter plasmids were transfected into MMC and treated with or without TGF-β1 (10 ng ml^−1^, 6–24 h)^11^,^28^. The fragment of upstream of lnc-MGC (miR-379 cluster) digested with SacI and HindIII was cloned into pGL4 luciferase vector (Promega). CHOP binding site mutant was created by PCR using CHOP site mutant primer 5′-GAGCTCTgcGCTCTgcGCACCTGCGCTT-3′ and miR379upR1, 5′-GACCCCTAGAAACTTGGGCCTTGTCCCTG-3′ and SacI/HindIII digested fragment was cloned into pGL4.

### Chromatin immunoprecipitation assays

ChIP assays were performed as previously described^11^,^28^. Briefly, MMCs were treated with TGF-β1 (10 ng ml^−1^) or HG (25 mM) and then cross-linked with formaldehyde. The cross-linked chromatin was sheared and immunoprecipitated with antibodies (∼10 μg ml^−1^) against Smad2/3, CHOP, USF1, ZEB1 or TFE3. ChIP-enriched DNA was purified and used as a template for real-time qPCR; the CHOP binding element in the promoter region of lnc-MGC was amplified with following primers: forward 5′- GAGCTCTTGCTCTTTGCACCTGCG-3′ and reverse 5′- AAGCAGGTGGAACCAGAAGTAAGCC-3′. ChIP-qPCR results (normalized enrichment) were calculated by the 2^−ΔΔC^_t_ method (where *C*_t_ is threshold cycle) and normalized to input DNA (purified from 5% of the same cross-linked chromatin).

### GapmeRs

GapmeRs are antisense oligonucleotides having a central stretch of DNA that is complementary to the target which is flanked by LNAs, and they induce RNAse H mediated target degradation. Twenty GapmeRs were designed *in silico* to inhibit the expression of lnc-MGC. These 20 GapmeRs fell into four clusters and four representative GapmeRs (one from each cluster) were synthesized and obtained from Exiqon (Vedbaek, Denmark) (MGC1, TCAaaaacataacGCC; MGC5, CACggtgctgaaaGAG; MGC8, TGAaggccacactAAC; MGC10, ATTtggcagtgggAAG, uppercase: LNA; lowercase: DNA, full phosphorothioate). We used a well-established and characterized control oligo provided by Exiqon which has the same modification as our lncRNA targeting GapmeRs and has no homology to any known mRNA, miRNA or lncRNA in mouse, rat and human (negative control A, AACacgtctataCGC). One of the most consistently effective oligos identified by *in vitro* screening was MGC10, while other GapmeRs had lesser effects on the expression of lnc-MGC. 2 or 5 mg kg^−1^ MGC10 was injected subcutaneously into control and STZ-injected diabetic C57BL6 mice. Human version of MGC10 (HMGC10) was designed based on the human sequence. HMGC10, (GATttggcattggAAG; uppercase: LNA; lowercase: DNA, full phosphorothioate).

### Serum toxicity analyses

Serum samples from the mice were sent to the IDEXX laboratory (Irvine, CA) for Preclinical Research Services (clinical biochemistry panels to evaluate liver and kidney toxicity).

### Serum cytokine profiles

Cytokine profiles in the mice sera were examined using a pre-mixed mouse 10-plex panel from Invitrogen that includes: GM-CSF, INF-g, IL-1b, IL-2, IL-4, IL-5, IL-6, IL-10, IL-12(P40/P70) and TNF-α at the Analytical Pharmacology Core Facility at City of Hope.

### *In situ* hybridization

To detect LNA-GapmeR MGC10 accumulation *in vivo, in situ* hybridization was performed as described previously^11^. Briefly, frozen kidney cortex sections from LNA-MGC10 injected and saline control mice (*n*=3 per group) were fixed in paraformaldehyde (3% for 10 min at room temperature) followed by rinsing in PBS. Slides were pre-hybridized (at 48 °C for 30 min) in hybridization solution (50% formamide, 5 × saline sodium citrate buffer (SSC), 500 μg ml^−1^ yeast transfer RNA and 1 × Denhardt’s solution), and then hybridized (at 48 °C for 5 min) with a TEX615 Red (Exiqon) fluorescent-labelled LNA-modified oligonucleotide probe complementary to LNA-MGC10 (16mer, 5′-TEX615-cTtcCcaCtgCcaAat-3′ (uppercase: LNA; lowercase: DNA; Exiqon) followed by post-hybridization wash three times at 55 °C in 0.1 × SSC. Slides were then stained with 4,6-diamidino-2-phenylindole, mounted and analysed using a fluorescence microscope.

### Histology and immunohistochemistry

Formalin-fixed, paraffin-embedded sections of mouse kidneys were mounted onto positively charged slides, deparaffinized, washed with water, blocked with Dako protein block (Dako, Carpinteria, CA), and incubated with EDEM3 antibody for 30 min. Slides were washed with Dako wash, treated with hydrogen peroxide for 5 min, washed with PBS, incubated with anti-rabbit secondary antibody conjugated with a peroxidase polymer (Dako, Carpinteria, CA), and washed and incubated with 3,3′-diaminobenzidine for 8 min. Slides were counterstained with haematoxylin and mounted. PAS staining was performed to analyse ECM deposition. Images were taken using Olympus BX51 microscope with In Studio (Pixera Corp., Santa Clara, CA) software to collect images. ImagePro software (Media Cybernetics Inc., Rockville, MD) was used to quantify staining.

### TUNEL assay

Combined TUNEL *in situ* staining and WT1 (Wilms Tumor) immunohistochemical staining was performed on frozen kidney sections from control and diabetic mice as well as mice groups injected with control oligo and MGC10. The *in situ* Cell Death Detection (TMR red) kit was purchased from Roche and WT1 antibody was purchased from Santa Cruz. Briefly, 5 μm sections were fixed and processed for TUNEL staining according to manufacturer’s instructions. The slides were then washed with PBS and processed for immunohistochemical staining of WT1 and mounted using Prolong Gold antifade reagent with 4,6-diamidino-2-phenylindole (Applied Biosystems) for visualization.

### Electron microscopy

EM structures of mouse kidney glomeruli were evaluated in the EM core of City of Hope. Paraffin-embedded tissue was deparaffinized with xylene, rehydrated in a graded ethanol series to distilled water and fixed with 2% glutaraldehyte in 0.1 M Cacodylate buffer (Na(CH_3_)_2_AsO_2_ 3H_2_O), pH7.2, at 4 °C, overnight. The samples were washed three times with 0.1 M Cacodylate buffer, pH 7.2, post-fixed with 1% OsO_4_ in 0.1 M Cacodylate buffer for 30 min and washed three times with 0.1 M Cacodylate buffer. The samples were then dehydrated through 60%, 70%, 80%, 95% ethanol, 100% absolute ethanol (twice), propylene oxide (twice) and were left in propylene oxide/Eponate (1:1) overnight at room temperature. The vials were sealed. The next day, the vials were left open for 2–3 h to evaporate the propylene oxide. The samples were infiltrated with 100% Eponate and polymerized at ∼64 °C for 48 h. For EM, ultra-thin sections (∼70 nm thick) were cut using a Leica Ultra cut UCT ultramicrotome with a diamond knife, picked up on 200 mesh copper EM grids. Grids were stained with 2% uranyl acetate for 10 min followed with Reynold’s lead citrate staining for 1 min. EM was done on an FEI Tecnai 12 transmission electron microscope equipped with a Gatan Ultrascan 2 K CCD camera. ImagePro software (Media Cybernetics Inc., Rockville, MD) was used to quantify GBM thickness, the distance between the endothelial and podocyte plasma membranes. More than 20 measurements were made in each group to determine an average GBM thickness.

### Measurement of cellular hypertrophy

Hypertrophy was assessed by measurement of cellular protein/cell counts^11^. MMC were trypsinized and counted using a Coulter Counter with 100-μm aperture (Beckman Coulter, Brea, CA). Cells were lysed, and total protein content was measured using protein assay kit from Bio-Rad.

### Human sample studies

Cluster miRNA expression was determined by small RNA sequencing in microdissected glomeruli from adult Pima Indians with type 2 diabetes who participated in a 6-year clinical trial to evaluate the renoprotective efficacy of losartan (ClinicalTrials.gov number NCT00340678)^57^. Of the 169 clinical trial participants, 121 underwent kidney biopsy and 46 of the participants who underwent kidney biopsies were included in the present study. This study was approved by the Institutional Review Board of the National Institute of Diabetes and Digestive and Kidney Diseases. The expression of cluster miRNA precursors was associated with morphometric parameters in 39 human nephrectomy samples. Age ranged from 31 to 85 years. Morphometric analysis was performed in formalin-fixed paraffin-embedded tissue sections combining PAS staining and WT1 as podocyte marker with quantitative image analysis as described^58^,^60^. This study was approved by the Institutional Review Board of the University of Michigan. Each subject gave written informed consent.

### Quantification of miRNAs from human renal biopsies

Glomeruli were microdissected from kidney biopsies, and nephrectomy tissue samples submerged in RNA*later* manually using a dissection microscope. Total RNA was isolated using spin-columns; miRNA expression was quantified using qRT-PCR performed using TaqMan Array Human MicroRNA Card (Applied Biosystems) and small RNA-sequencing^58^. Samples were normalized to geometric mean of reference RNAs (refs 17, 58). Expression of miRNA precursors was determined in microdissected glomeruli of nephrectomy samples using Affymetrix Human Gene 2.1 ST 24-Array^58^,^68^.

### Statistical analysis

Normal distribution of each sample group was confirmed by *χ*^2^ test before comparison between groups. Statistical analyses were performed by Student’s *t*-tests (two-sided) to compare two groups (planned comparison) and Benjamini–Hochberg adjusted for multiple comparisons^69^. If variances between two groups were significantly different (F-test), *t*-test assuming unequal variances was used in the comparison. *P*<0.05 was considered as statistically significant. Power analyses for the animal experiments have been described earlier.

### Data availability

The authors declare that all data supporting the findings of this study are available within the article and its Supplementary Information Files or are available from the corresponding authors on request. The sequencing data sets (including both raw sequences and processed data) have been deposited in the NCBI Gene Expression Omnibus (GEO) database (GEO accession number GSE69718, https://www.ncbi.nlm.nih.gov/geo/query/acc.cgi?acc=GSE69718).

## Additional information

**How to cite this article:** Kato, M. *et al*. An endoplasmic reticulum stress-regulated lncRNA hosting a microRNA megacluster induces early features of diabetic nephropathy. *Nat. Commun.* 7:12864 doi: 10.1038/ncomms12864 (2016).

## Supplementary Material

Supplementary Information Supplementary figures 1-39, Supplementary tables 1-3, Supplementary references
