# Supplementary material for: An endoplasmic reticulum stress-regulated lncRNA hosting a microRNA megacluster induces early features of diabetic nephropathy
Source: Nat Commun. 2016 Sep 30;7:12864. doi: 10.1038/ncomms12864 (PMC5553130; doi:10.1038/ncomms12864)
Supplement: Supplementary Information — Supplementary figures 1-39, Supplementary tables 1-3, Supplementary references [file ncomms12864-s1.pdf]

Supplementary Figure 1

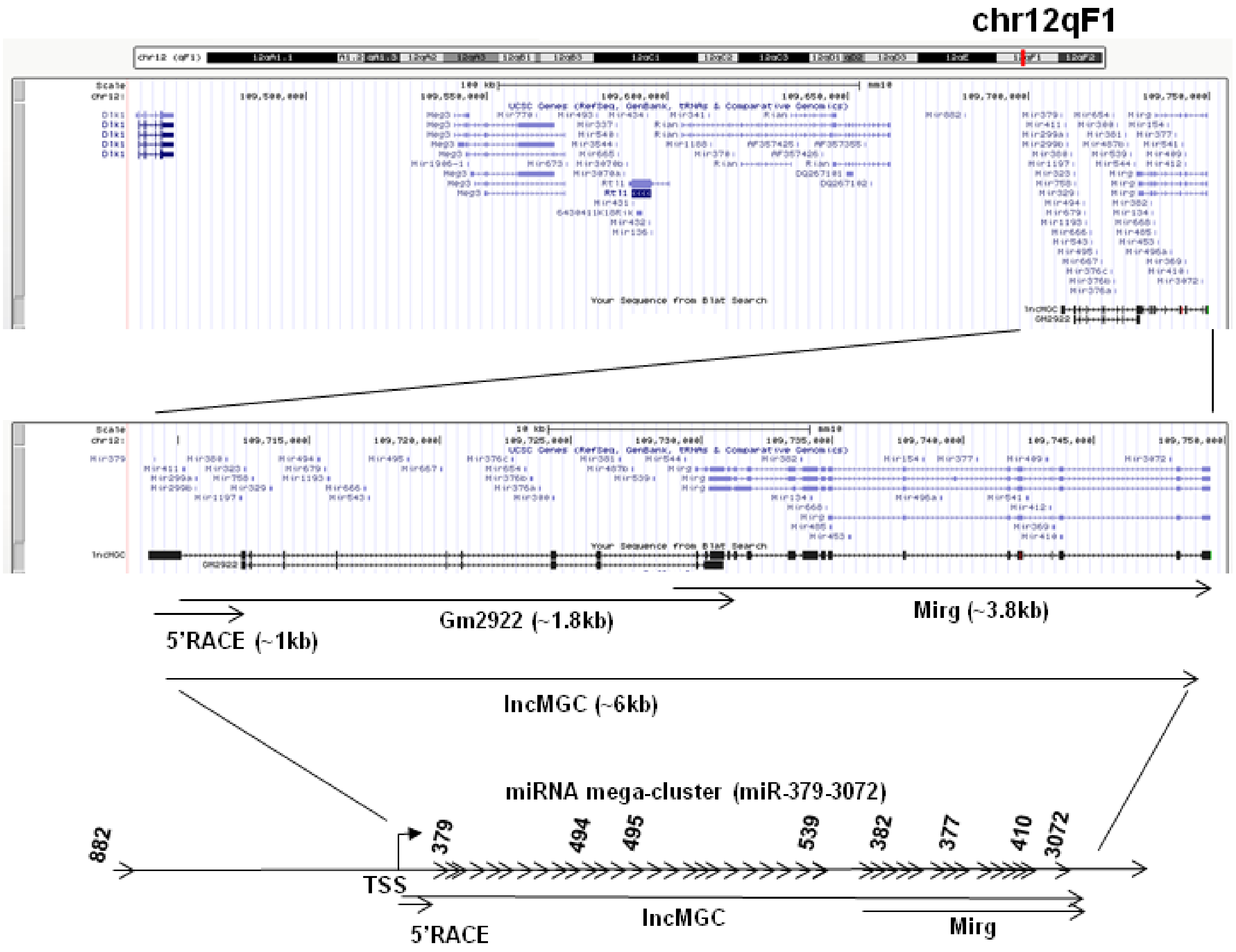

Supplementary Figure 1. Genome structure of lnc-MGC. Genome organization shows that the mouse miR-379 megacluster is located within the largest miRNA cluster currently identified in the genome. It maps within the *DLK-DIO3* genomic region (mouse chr 12, human chr14), which is home to several miRNAs and lncRNAs. In the mouse genome, this locus is located at chr12qF1 (top). Several transcripts have been identified in this region, although all of these transcripts appear to be parts of a single long transcript. Because of rapid processing of this primary transcript, the complete large transcript could not be annotated and cloned by conventional approaches. However, partial fragments were cloned, and the figure depicts an assembled transcript from cloned and deposited sequences (in the database) which is shown along with the UCSC Genome browser. We named the longest noncoding RNA (lncRNA) as "lnc-MGC", the 3' region of which overlaps with Mirg and middle region with Gm2922, which are also other ncRNAs in this region. The 5' region, which starts from transcription start site (TSS) of lnc-MGC, was cloned by 5' RACE. miR-882 is located far-upstream of the miR-379 cluster and not covered by lnc-MGC.

Supplementary Figure 2

a

copy/cell

SD	TGFb	NG	HG
3.510812	3.94908	3.986743	5.339276
3.650703	4.357102	3.923746	5.06511
3.774682	4.666205	3.963863	4.776591
3.64926	5.486623	3.872292	4.472395
3.651726	5.451668	3.66281	4.451617
3.732771	4.955751	3.617369	4.744039

b

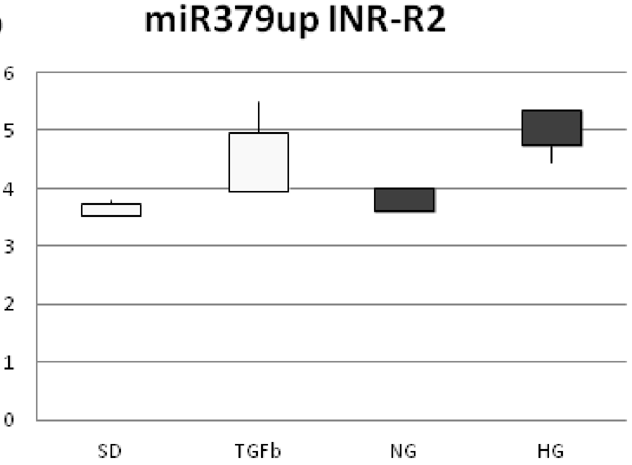

Supplementary Figure 2. Copy numbers of lnc-MGC in mouse mesangial cells. Copy numbers per cell of the upstream part (from initiator to just upstream of miR-379, INR-R2) of lncMGC were calculated by using the cloned fragment of INR-R2 (5' region of lncMGC) as standard (serial dilution of the fragment). Absolute numbers of INR-R2 molecules were calculated from absolute amount of INR-R2 fragments by real time qPCR. RNA was extracted from MMC (the numbers of cells were counted). Copy numbers of lncMGC per cell were calculated as absolute numbers of lncMGC molecules in sample RNAs divided by total cell number. Copy numbers of lncMGC were 3-4 copies per cell in control cells, and 4~6 copies per cell in HG or TGF-β treated mesangial cells Data shows exact numbers in six cultures (a) and bar graph depiction (b).

Supplementary Figure 3

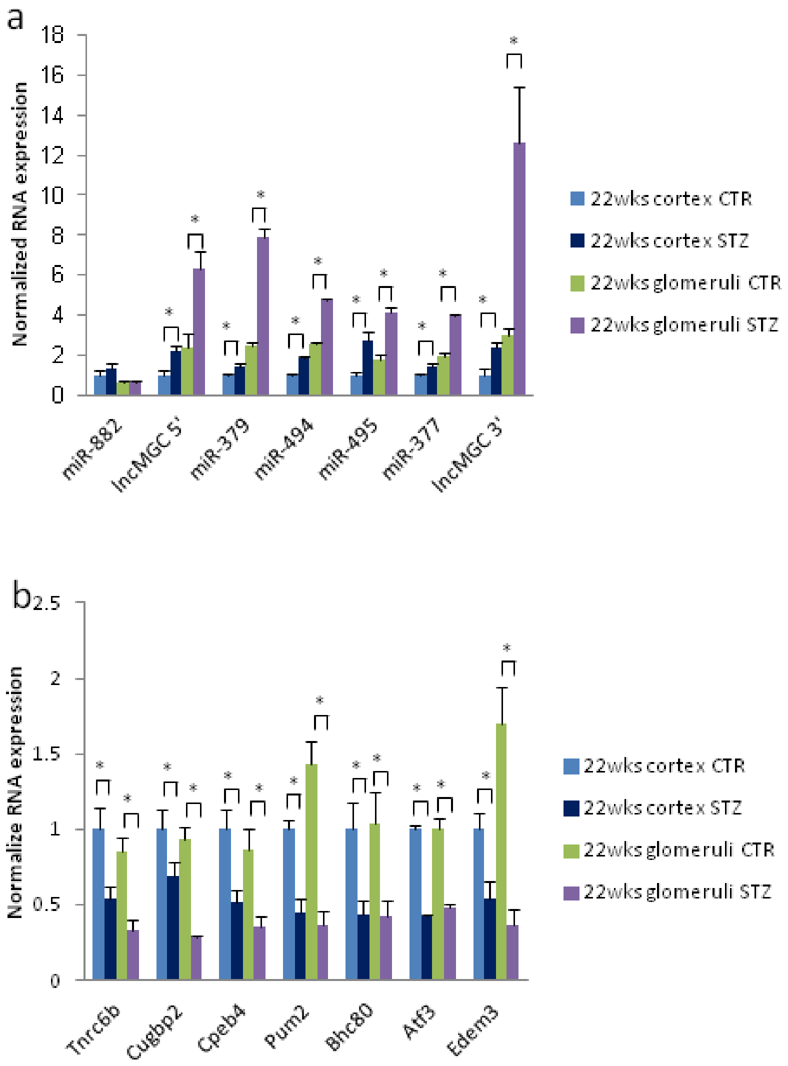

Supplementary Figure 3. The host lnc-RNA (lnc-MGC) transcript of the megacluster of miRNAs (miR-379 cluster) and key component miRNAs are up-regulated in the glomeruli of C57BL/6 mice with longer duration of diabetes and significant renal dysfunction (22 weeks after onset of diabetes). (a) The expression of lnc-MGC and four cluster miRNAs in glomeruli from STZ diabetic mice (cortex and glomeruli) relative to controls (vehicle injected CTR). Five mice in each group. (b) Significant lower expression of potential targets of miR-379 cluster in kidney glomeruli from diabetic mice (STZ) than the non-diabetic control mice. Results are mean + SE. Five mice in each group. *, $P<0.05$.

a **Potential targets** Supplementary Figure 4

RNA binding proteins

Translational regulators (CUGBP2, PUM2, TNRC6B, CPEB2/4, Rc3h1, HuR)

RNA splicing factors (CUGBP2, Dxd3x, SFRS1, COX-2: VEGF alternative splicing)

Transcription factors

FOXP2, Arid2, NF1a/b,

Zinc-finger proteins (Zfx, Zfp148, Trps1, KLF12, KLF3)

Cofactors PHF21A (BHC80 unmethylated histone biding protein in repressor complex)

ER stress-related EDEM3, ATF3

Others PTEN (Akt activation & protein synthesis)

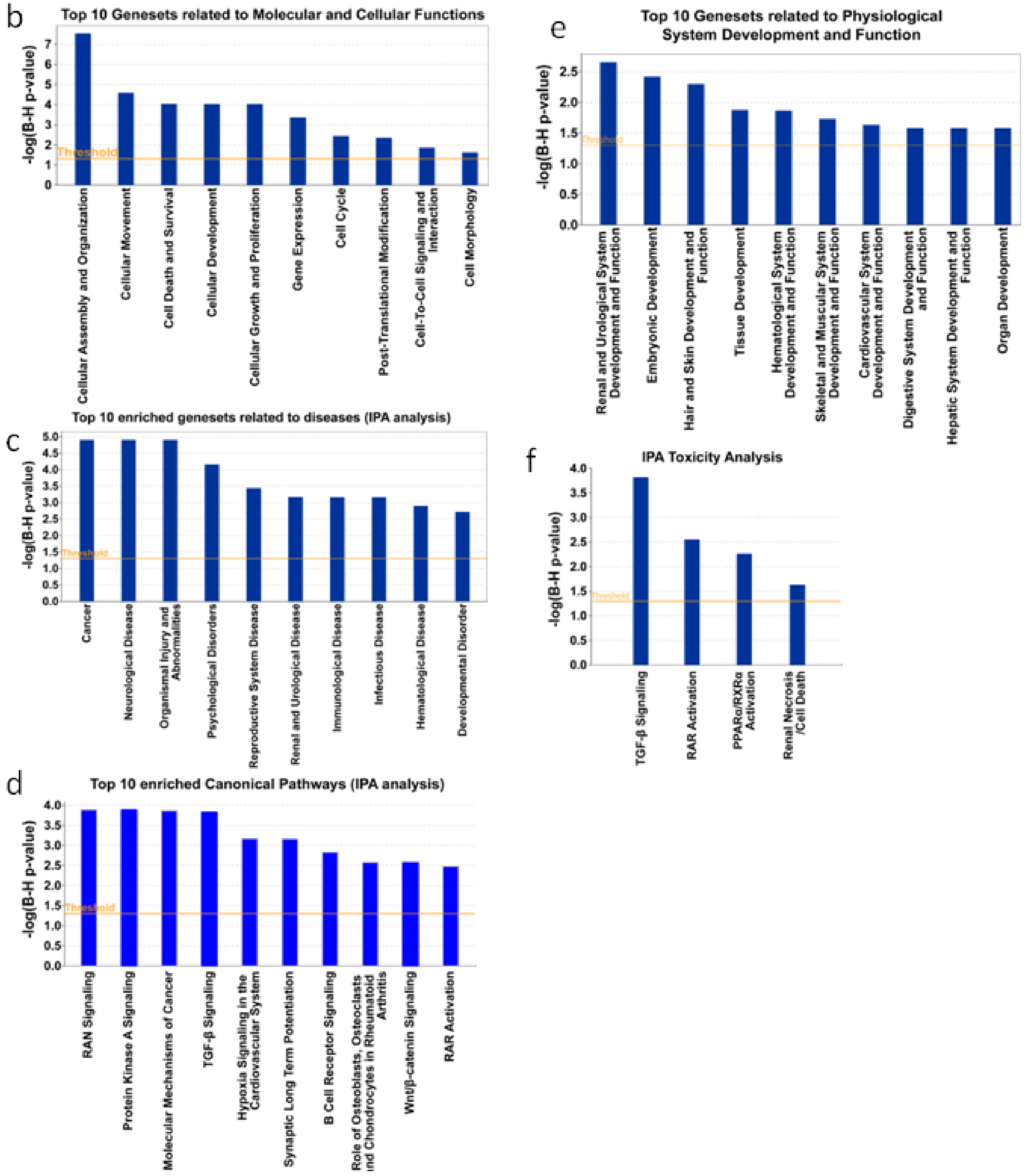

Supplementary Figure 4. (a) IPA analysis on potential human gene targets of miR-379 cluster. The potential human gene targets containing at least 3 conserved binding sites for each miRNA in the cluster were predicted by seed-based algorithm[1] of TargetScan (http://www.targetscan.org/). All the target genes were then pooled to generate a target gene set. Ingenuity pathway analyses (IPA) were applied to this gene set to identify statistically-enriched biological functions and pathways at Benjamini-Hochberg (B-H) adjusted p values < 0.05 by right-tailed Fisher's exact tests. The significance level was presented in the y-axis as -log10 (B-H P value) in the barplots. (b-f) Human gene targets of the cluster miRNAs related to their molecular cellular functions (b), gene sets related to diseases (c), enriched canonical pathways (d), physiological development and function (e) and toxicity (f). For analyses with more than 10 categories identified, the top 10 significant ones were displayed.

Supplementary Figure 5

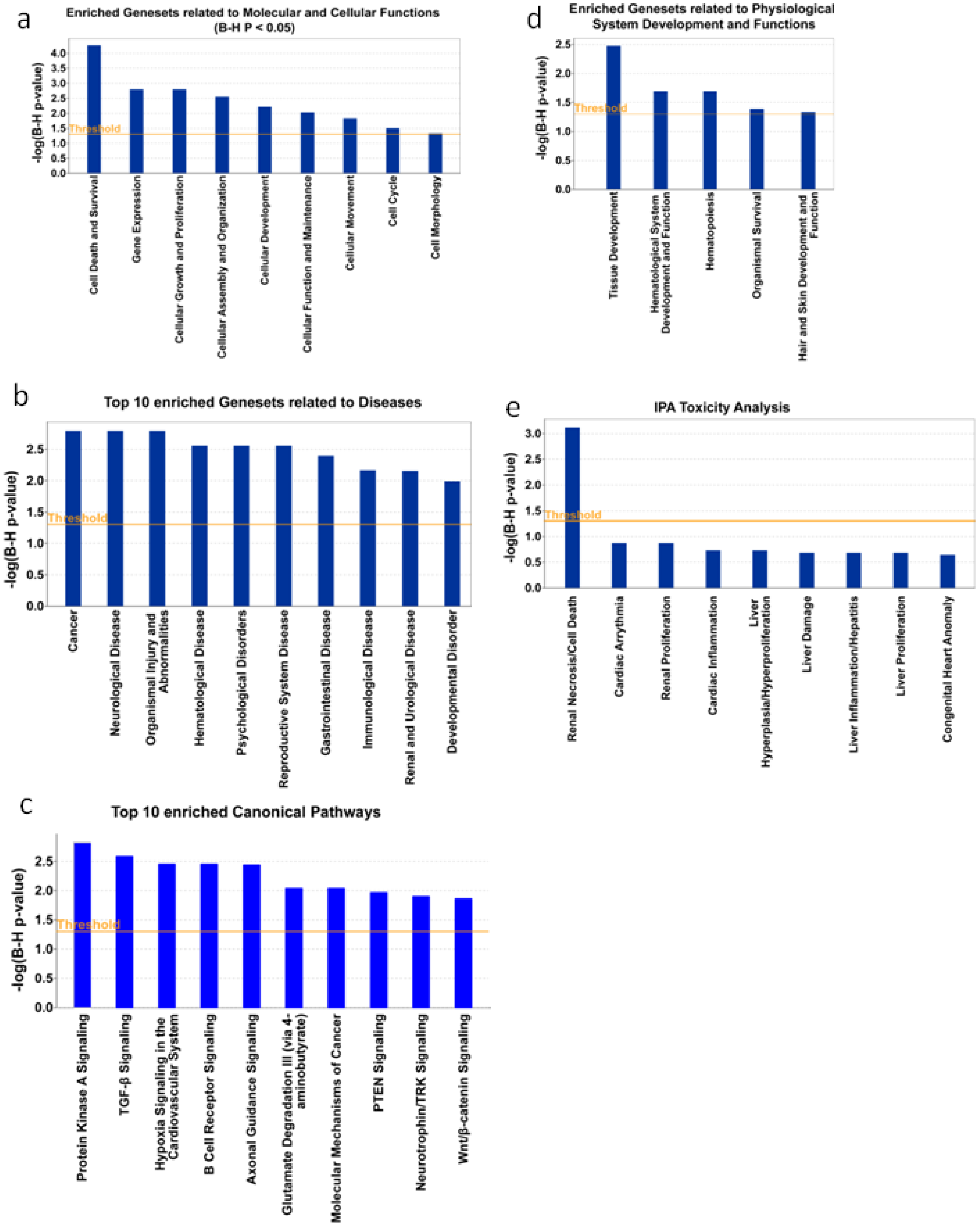

Supplementary Figure 5. IPA analysis on potential mouse target genes of the miR-379 cluster as predicted by TargetScan mouse. Similar to the human target genes evaluated by seed-based algorithm[1] of TargetScan (http://www.targetscan.org/) in Supplementary Figure S4, the IPA analyses here included statistically-enriched molecular cellular functions (a), genesets related to diseases (b), enriched canonical pathways (c), physiological development and function (d) and toxicity (e). Please refer to the legend of Supplementary Figure S4 for further details.

Supplementary Figure 6

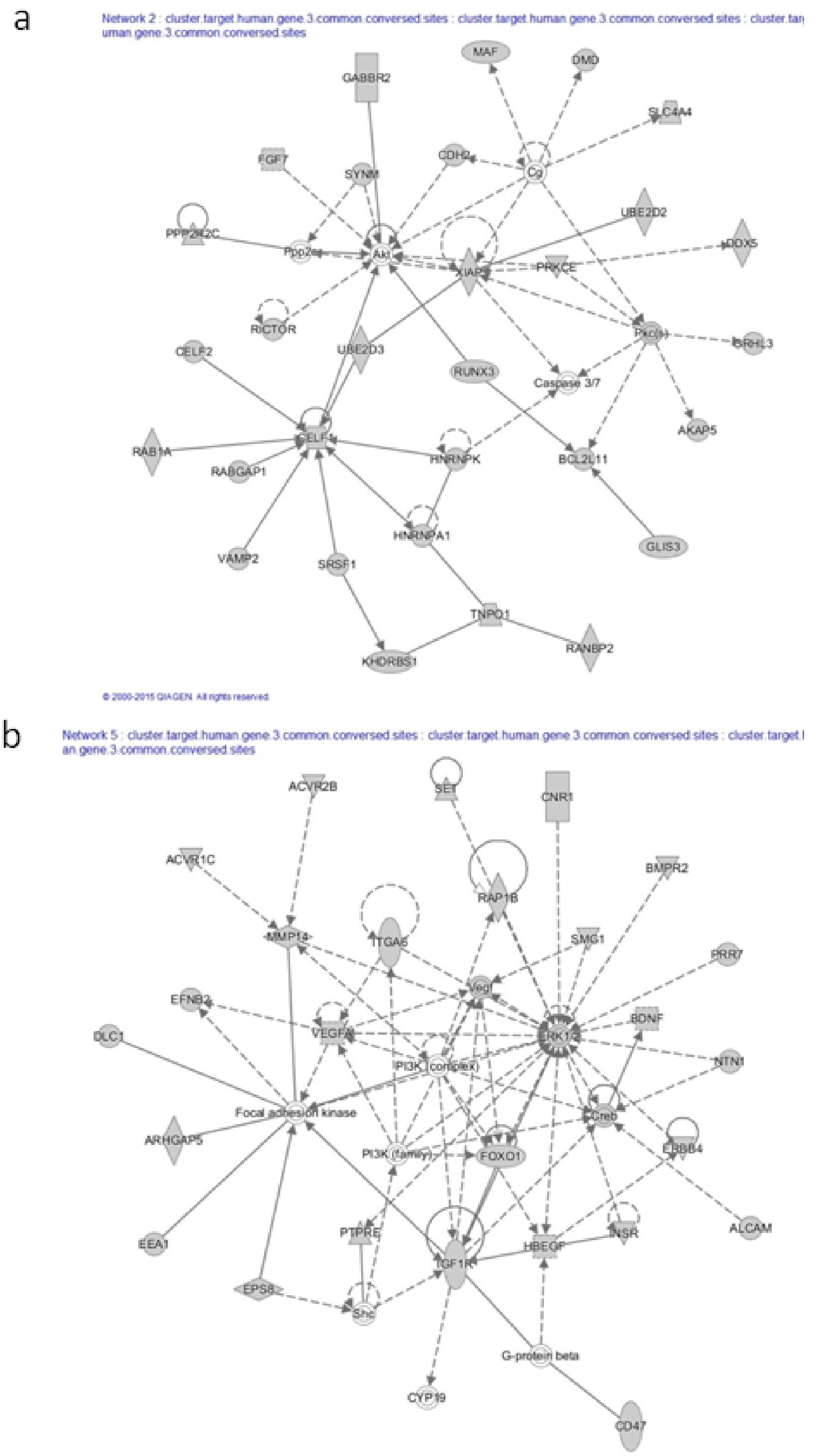

Supplementary Figure 6. IPA network analysis on potential targets of miR-379 cluster. The network analysis depicted relationships of the potential target genes of the miRNA cluster to Akt (a) and Erk (b) kinases.

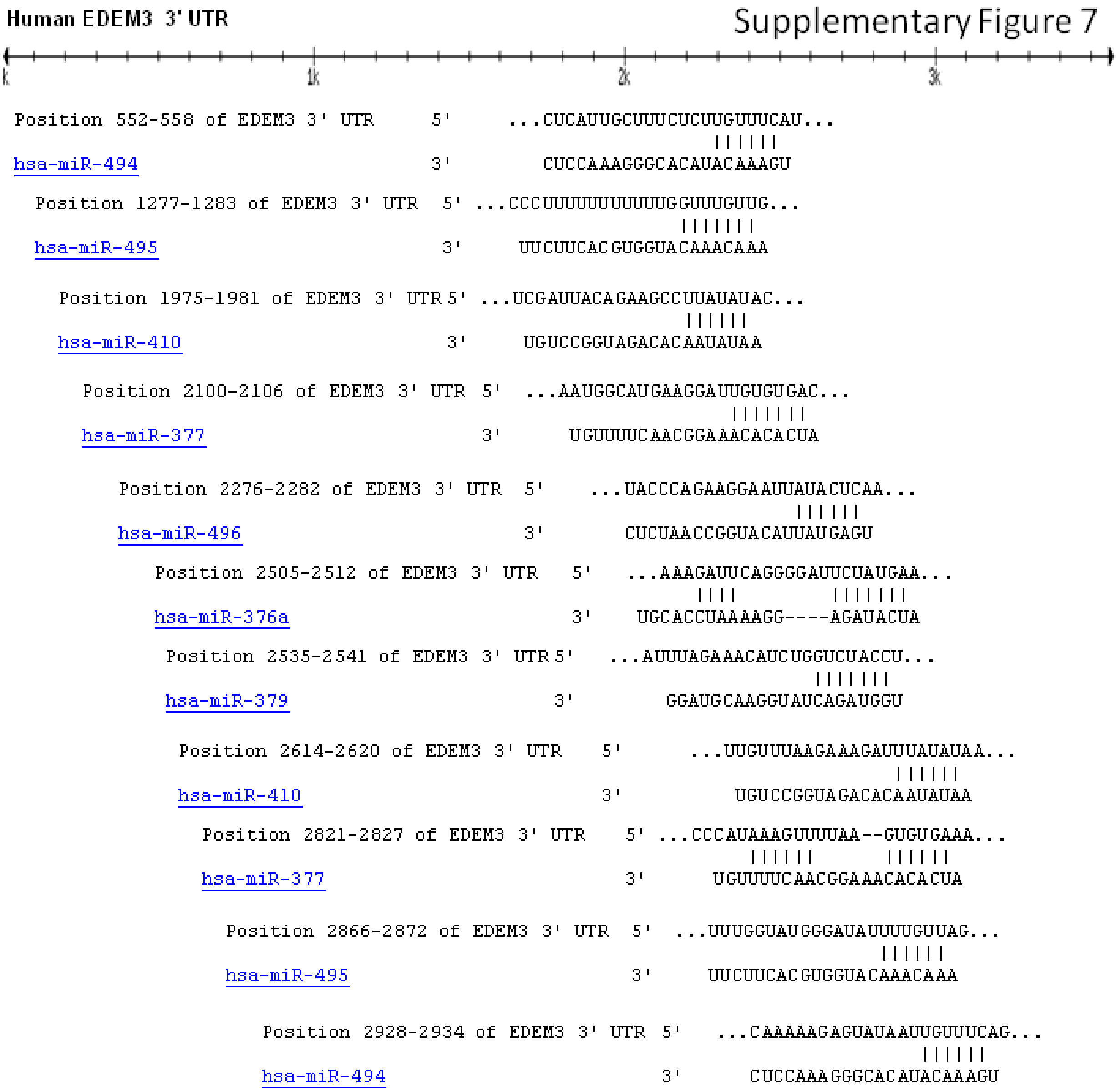

Supplementary Figure 7. The location of the binding sites of multiple cluster miRNAs in the 3'UTR of human *EDEM3* obtained from seed-based algorithm[1] of TargetScan (http://www.targetscan.org/) are shown.

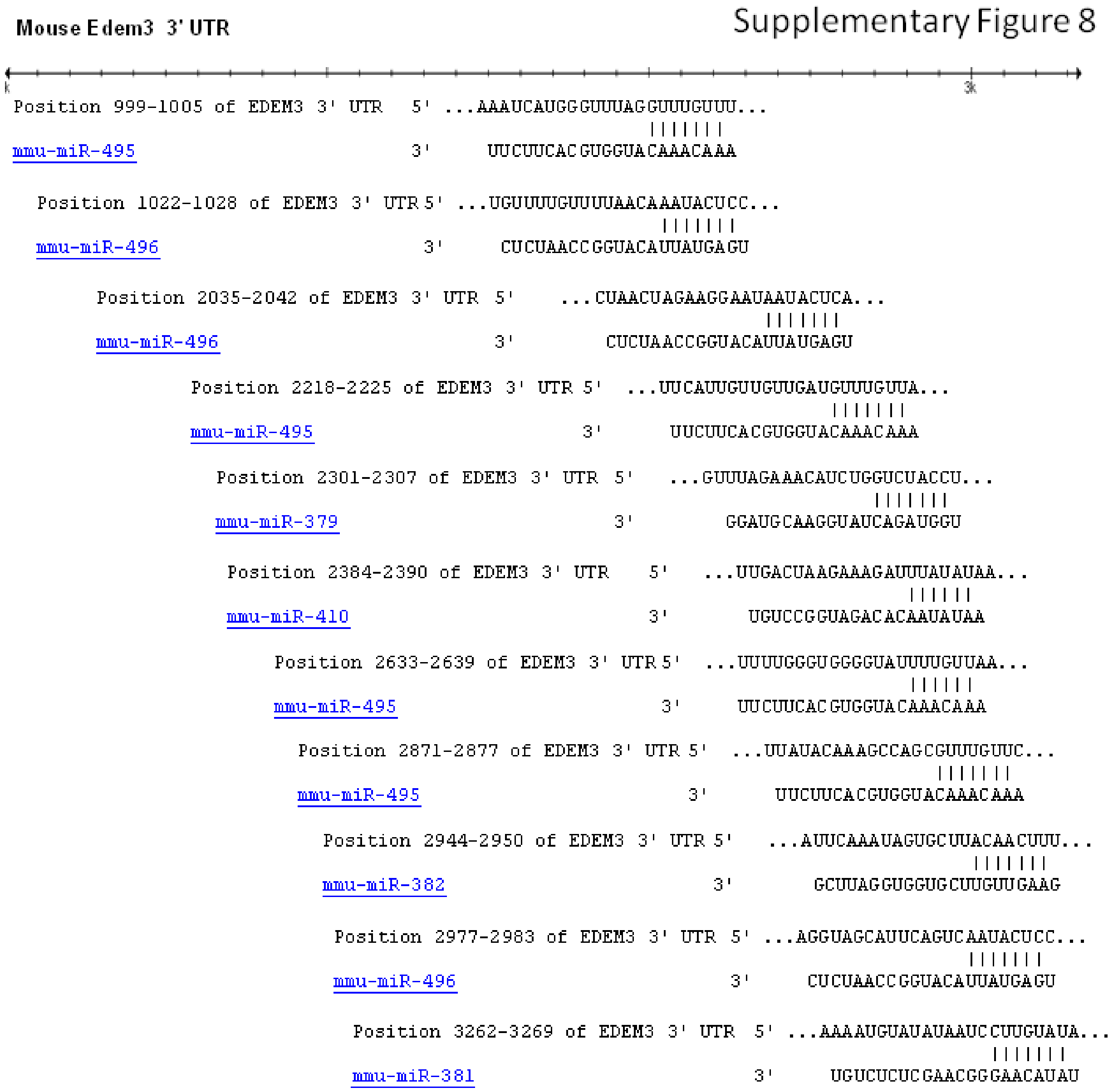

Supplementary Figure 8. The location of the binding sites of multiple cluster miRNAs in the 3'UTR of mouse *Edem3* obtained from seed-based algorithm[1] of TargetScan (http://www.targetscan.org/) are shown.

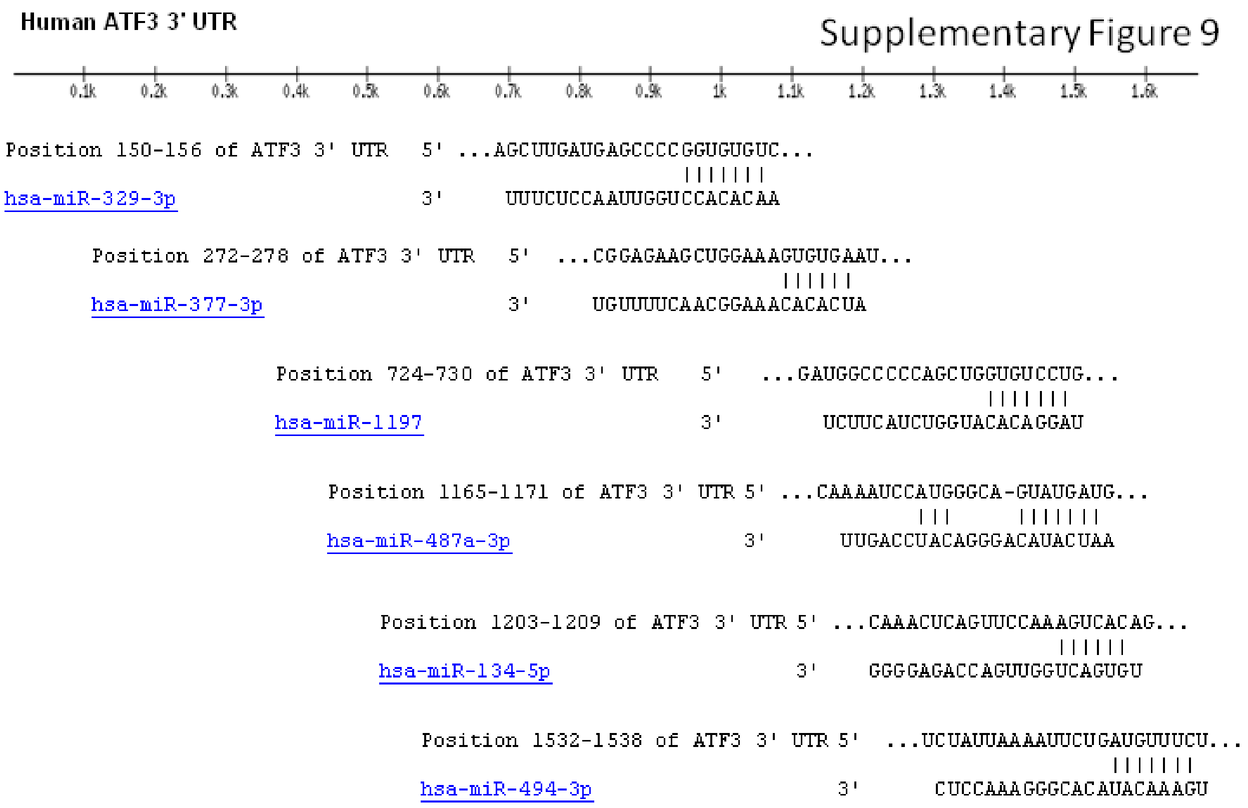

Supplementary Figure 9. The location of the binding sites of multiple cluster miRNAs in the 3'UTR of human *ATF3* obtained from seed-based algorithm[1] of TargetScan (http://www.targetscan.org/) are shown.

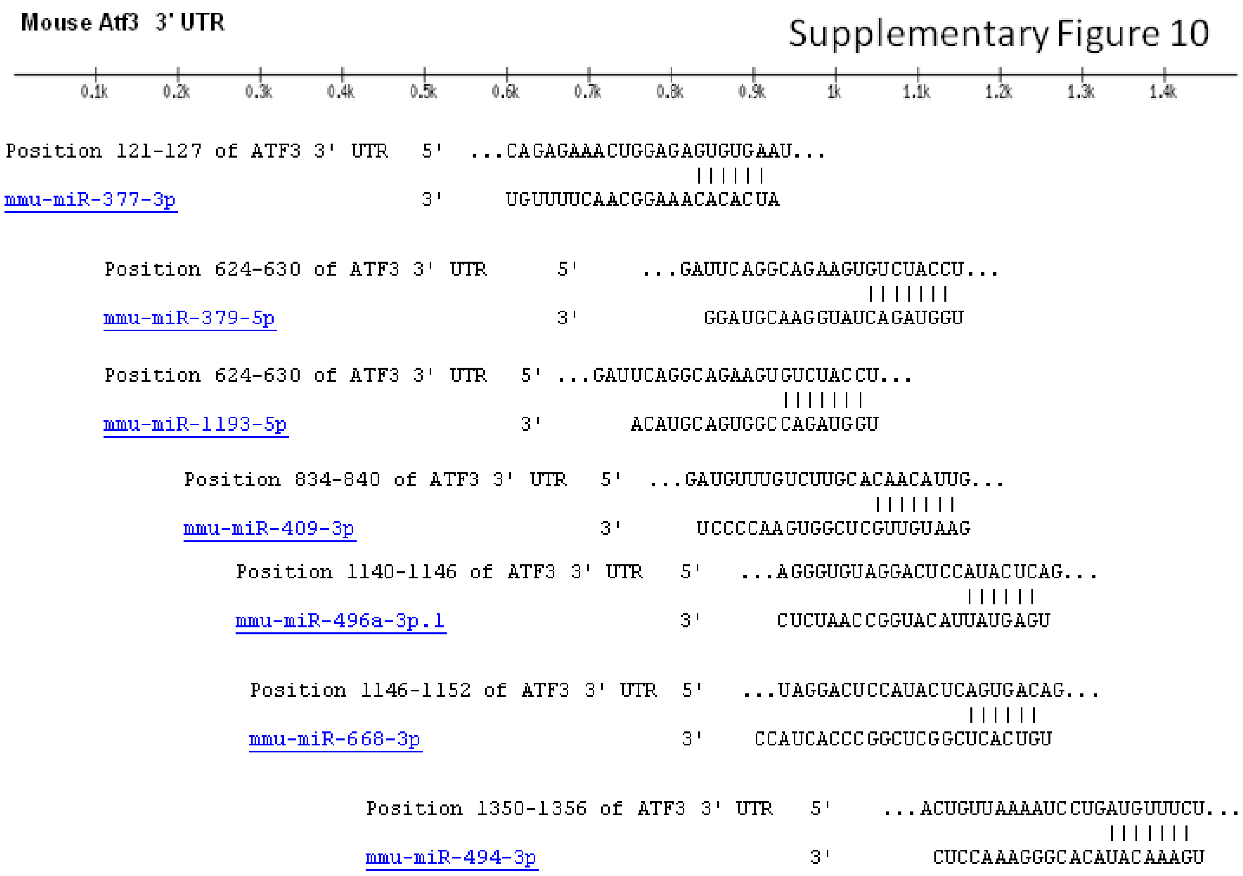

Supplementary Figure 10. The location of the binding sites of multiple cluster miRNAs in the 3'UTR of mouse *Atf3* obtained from seed-based algorithm[1] of TargetScan (http://www.targetscan.org/) are shown.

Human TNRC6B 3' UTR

Supplementary Figure 11

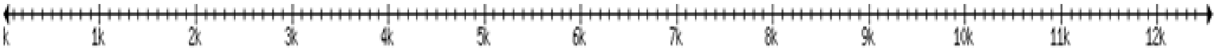


```
Position 353-359 of TNRC6B 3' UTR      5'    ...UUUCACUUUUUUCAUGUUAUAUG...
                                                                 |||||||
hsa-miR-410                            3'          UGUCCGGUAGACACAAUAUAA

Position 442-449 of TNRC6B 3' UTR   5' ...UGUUUCCGUCCUUAGGUCUACCA...
                                                      |||||||
hsa-miR-379                         3'     GGAUGCAAGGUAUCAGAUGGU

Position 423-429 of TNRC6B 3' UTR     5'  ...ACUUUGUUUGCACUGGGUGUGUU...
                                                 |||       |||||||
hsa-miR-329                           3'    UUUCUCCAAUUGGU--CCACACAA

Position 512-518 of TNRC6B 3' UTR     5'   ...GGGAAGACUUGACGGAGCCUCAC...
                                                          ||||||
hsa-miR-485-5p                        3'      CUUAAGUAGUGCCGGUCGGAGA

Position 1431-1437 of TNRC6B 3' UTR   5'  ...AAACGUUUACUUAACGAAUGUUC...
                                                          |||||||
hsa-miR-543                           3'      UUCUUCACGUGGCGCUUACAAA

Position 1447-1453 of TNRC6B 3' UTR   5' ...AAUGUUCUUAAACCAGUGUGUAC...
                                                 ||||     ||||||
hsa-miR-329                           3'    UUUCUCCAAUUGGUCCACACAA

Position 3481-3488 of TNRC6B 3' UTR      5'   ...UUGGAUUGCUGAGAAUCUAUGUA...
                                                               |||||||
hsa-miR-376c                             3'        UGCACCUUAAAGGAGAUACAA

Position 4447-4454 of TNRC6B 3' UTR   5'  ...UUUGGCAAGAGCCAUGUCUACUA...
                                                            |||||||
hsa-miR-411                           3'       GCAUGCGAUAUGCCAGAUGAU

Position 4511-4517 of TNRC6B 3' UTR     5' ...AGUGCCUUCUUCUCUUCUACCAC...
                                                   |||       ||||||
hsa-miR-379                             3'    GGAUGCAAGGUAUC-AGAUGGU

Position 9935-9941 of TNRC6B 3' UTR   5' ...UGGUGAAACCCCGUCUCUACUAA...
                                                              ||||||
hsa-miR-411                           3'     GCAUGCGAUAUGCCAGAUGAU

Position 12141-12147 of TNRC6B 3' UTR   5'  ...AAAAACAAACAAUAAAAUGUUAA...
                                                                ||||||
hsa-miR-543                             3'     UUCUUCACGUGGCGCUUACAAA

Position 12151-12157 of TNRC6B 3' UTR     5'    ...AAUAAAAUGUUAAACUCUACUAA...
                                                                   ||||||
hsa-miR-411                               3'        GCAUGCGAUAUGCCAGAUGAU
```

Supplementary Figure 11. The location of the binding sites of multiple cluster miRNAs in the 3'UTR of human *TNRC6B* obtained from seed-based algorithm[1] of TargetScan (http://www.targetscan.org/) are shown.

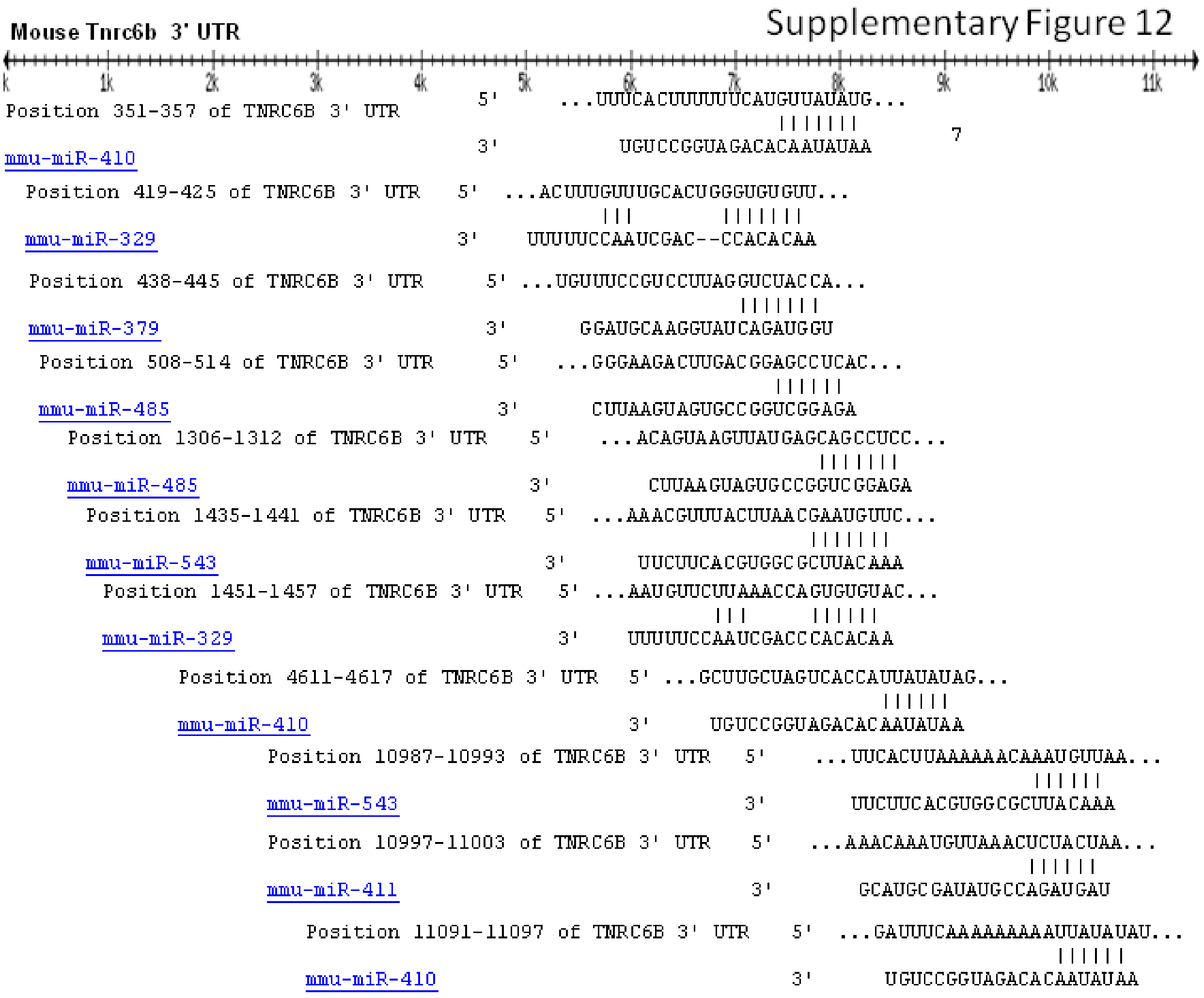

Supplementary Figure 12. The location of the binding sites of multiple cluster miRNAs in the 3'UTR of mouse *Tnrc6b* obtained from seed-based algorithm[1] of TargetScan (http://www.targetscan.org/) are shown.

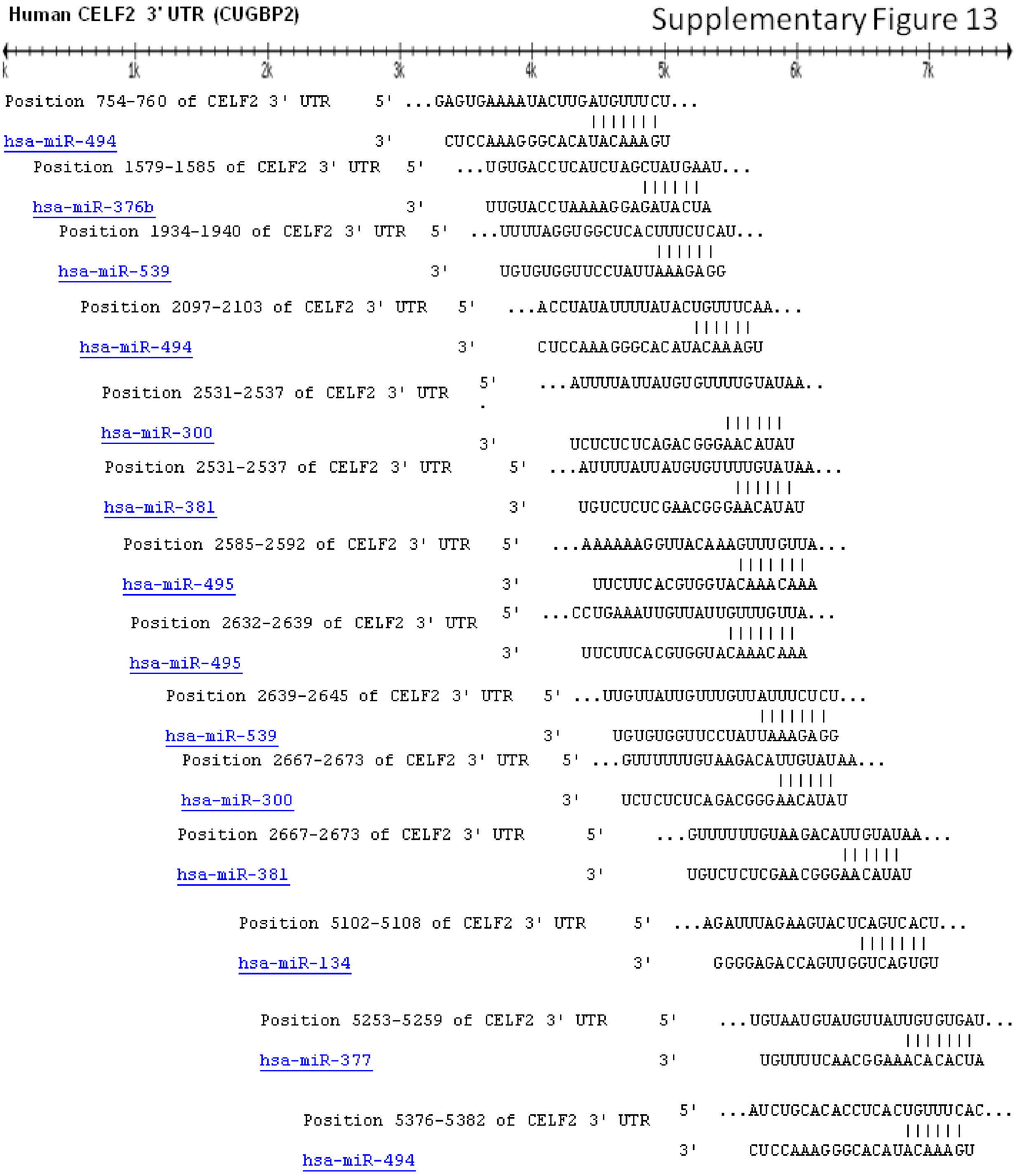

Supplementary Figure 13. The location of the binding sites of multiple cluster miRNAs in the 3'UTR of human *CUGBP2* obtained from seed-based algorithm[1] of TargetScan (http://www.targetscan.org/) are shown.

Mouse Celf2 3' UTR (Cugbp2) Supplementary Figure 14

```
k    1k    2k    3k    4k    5k    6k    7k

Position 756-762 of CELF2 3' UTR     5' ...GAGUGAAAAUACUUGAUGUUUCU...
                                                      |||||||
mmu-miR-494                          3'    CUCCAAAGGGCACAUACAAAGU

Position 1574-1580 of CELF2 3' UTR   5'  ...UGUGACCUCAUCUAGCUAUGAAU...
                                                      ||||||
mmu-miR-376b                         3'      UUCACCUACAAGGAGAUACUA

Position 1670-1676 of CELF2 3' UTR   5'  ...AAACCUGUGGCAAAAAUGUUUCU...
                                                      |||||||
mmu-miR-494                          3'      CUCCAAAGGGCACAUACAAAGU

Position 1931-1937 of CELF2 3' UTR   5'  ...UUUUAGGUGGCUCACUUUCUCAU...
                                                      ||||||
mmu-miR-539-5p                       3'     UGUGUGGUUCCUAUUAAAGAGG

Position 2558-2564 of CELF2 3' UTR   5'  ...AUUUUAUUAUGUGUUUUGUAUAA...
                                                      ||||||
mmu-miR-381                          3'     UGUCUCUCGAACGGGAACAUAU

Position 2558-2564 of CELF2 3' UTR   5'  ...AUUUUAUUAUGUGUUUUGUAUAA...
                                              |||||     ||||||
mmu-miR-539-3p                       3'     UUUUUCUUUAAUAGG---AACAUAC

Position 2611-2618 of CELF2 3' UTR   5'  ...AAAAAAGGUUACAAAGUUUGUUA...
                                                      |||||||
mmu-miR-495                          3'      UUCUUCACGUGGUACAAACAAA

Position 2658-2665 of CELF2 3' UTR   5'  ...CCUGAAAUUGUUAUUGUUUGUUA...
                                                      |||||||
mmu-miR-495                          3'      UUCUUCACGUGGUACAAACAAA

Position 2665-2671 of CELF2 3' UTR   5' ...UUGUUAUUGUUUGUUAUUUCUCU...
                                                      |||||||
mmu-miR-539-5p                       3'     UGUGUGGUUCCUAUUAAAGAGG

Position 2693-2699 of CELF2 3' UTR   5'    ...GUUUUUUGUAAGACAUUGUAUAA...
                                                         ||||||
mmu-miR-381                          3'       UGUCUCUCGAACGGGAACAUAU

Position 2693-2699 of CELF2 3' UTR   5'  ...GUUUUUUGUAAGACAUUGUAUAA...
                                                       ||||||
mmu-miR-539-3p                       3'     UUUUUCUUUAAUAGGAACAUAC

                                     5' ...AUUUGAUUUAAUGUGUGUGUGAA...
Position 3576-3583 of CELF2 3' UTR                        |||||||
                                     3'    UGUUUUCAACGGAAACACACUA
mmu-miR-377

Position 4081-4088 of CELF2 3' UTR   5'    ...AGGGCUUGGUUUUGCAUGUUUCA...
                                                           |||||||
mmu-miR-494                          3'       CUCCAAAGGGCACAUACAAAGU

Position 4977-4983 of CELF2 3' UTR   5'  ...AGAUUUAGAAGUACUCAGUCACU...
                                                         |||||||
mmu-miR-134                          3'     GGGGAGACCAGUUGGUCAGUGU

Position 5128-5134 of CELF2 3' UTR   5'   ...UGUAAUGUAUGUUAUUGUGUGAU...
                                                          |||||||
mmu-miR-377                          3'      UGUUUUCAACGGAAACACACUA
```

Supplementary Figure 14. The location of the binding sites of multiple cluster miRNAs in the 3'UTR of mouse *Cugbp2* obtained from seed-based algorithm[1] of TargetScan (http://www.targetscan.org/) are shown.

Human CPEB4 3' UTR

Supplementary Figure 15

k 1k 2k 3k 4k

```
Position 954-961 of CPEB4 3' UTR       5'  ...AUAUCCUAAAUUUACUAAUACUCA...
                                                      |||||||
hsa-miR-496                            3'      CUCUAACCGGUACAUUAUGAGU

Position 2127-2133 of CPEB4 3' UTR     5'       ...UGCACAUAAUGUAUAUUUGUUAU...
                                                               ||||||
hsa-miR-495                            3'          UUCUUCACGUGGUACAAACAAA

Position 2144-2150 of CPEB4 3' UTR     5' ...UGUUAUGCACUACUUUUGUAUAU...
                                                         ||||||
hsa-miR-300                            3'    UCUCUCUCAGACGGGAACAUAU

Position 2144-2150 of CPEB4 3' UTR     5'  ...UGUUAUGCACUACUUUUGUAUAU...
                                                         ||||||
hsa-miR-381                            3'     UGUCUCUCGAACGGGAACAUAU

Position 2488-2495 of CPEB4 3' UTR     5' ...UAAUUUAAGAAAUAG-UCUAUGUA...
                                                 |||     |||||||
hsa-miR-376c                           3'        UGCACCUUAAAGGAGAUACAA

Position 2772-2779 of CPEB4 3' UTR     5' ...GCCUGCCUUGGGACAGGUGUGUA...
                                                         |||||||
hsa-miR-329                            3'      UUUCUCCAAUUGGUCCACACAA

Position 2816-2822 of CPEB4 3' UTR     5'  ...GUAAGAAAUUGUUAUUCUACUAA...
                                                          ||||||
hsa-miR-411                            3'      GCAUGCGAUAUGCCAGAUGAU

Position 2838-2844 of CPEB4 3' UTR     5' ...AUAUUUACUUGUGAUUGUGUGAC...
                                                 |||     |||||||
hsa-miR-377                            3'        UGUUUUCAACGGAAACACACUA

Position 3146-3153 of CPEB4 3' UTR     5' ...UGUUUCUGAUUAUUU--UCUAUGUA...
                                                       ||||  |||||||
hsa-miR-376c                           3'              UGCACCUUAAAGGAGAUACAA

Position 3228-3234 of CPEB4 3' UTR     5'  ...CCAUACACAGACCUCUGCAGAAU...
                                                          |||||||
hsa-miR-544                            3'      CUUGAACGAUUUUUACGUCUUA

Position 3544-3550 of CPEB4 3' UTR     5'  ...UCAGAAAUCAAUCUUUCUACUAA...
                                                          ||||||
hsa-miR-411                            3'      GCAUGCGAUAUGCCAGAUGAU

Position 3738-3744 of CPEB4 3' UTR     5'  ...AAUUUUCAUCAGAUUUUUGUUAU...
                                                          ||||||
hsa-miR-495                            3'     UUCUUCACGUGGUACAAACAAA

Position 3741-3747 of CPEB4 3' UTR     5' ...UUUCAUCAGAUUUUUGUUAUAUU...
                                                        |||||||
hsa-miR-410                            3'      UGUCCGGUAGACACAAUAUAA

Position 4011-4018 of CPEB4 3' UTR     5' ...AUGUUCUACUCUUAAGUUAUAUA...
                                                        |||||||
hsa-miR-410                            3'      UGUCCGGUAGACACAAUAUAA
```

Supplementary Figure 15. The location of the binding sites of multiple cluster miRNAs in the 3'UTR of human *CPEB4* obtained from seed-based algorithm[1] of TargetScan (http://www.targetscan.org/) are shown.

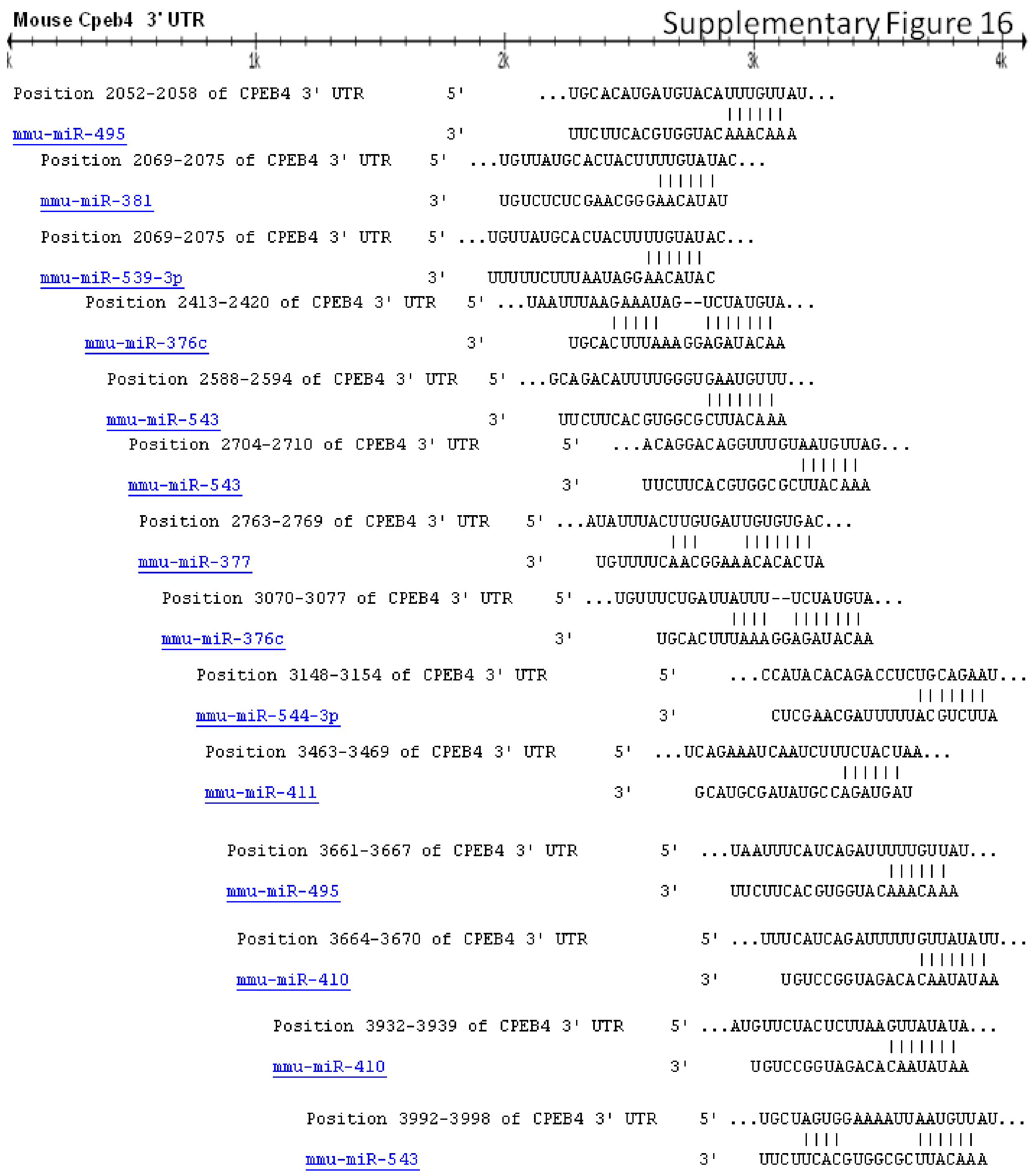

Supplementary Figure 16. The location of the binding sites of multiple cluster miRNAs in the 3'UTR of mouse *Cpeb4* obtained from seed-based algorithm[1] of TargetScan (http://www.targetscan.org/) are shown.

Human PHF21A 3' UTR Supplementary Figure 17

```
Position 93-99 of PHF21A 3' UTR      5' ...CGGAUUUCUGGAAAGUGCAGAAU...
                                                      |||||||
hsa-miR-544                          3'    CUUGAACGAUUUUUACGUCUUA
Position 184-190 of PHF21A 3' UTR    5' ...GAUCCUUCUUAUUCU-GUGUGUAC...
                                                 |||     ||||||
hsa-miR-329                          3'    UUUCUCCAAUUGGUCCACACAA

Position 309-315 of PHF21A 3' UTR    5'    ...AGUGUGUGCCUGCAGCAGCCUCC...
                                                         |||||||
hsa-miR-485-5p                       3'       CUUAAGUAGUGCCGGUCGGAGA

Position 330-336 of PHF21A 3' UTR    5'  ...CCACAGCCACGAUGGGUUUGUUU...
                                                         |||||||
hsa-miR-495                          3'     UUCUUCACGUGGUACAAACAAA

Position 410-416 of PHF21A 3' UTR    5' ...CUUACUUGCCGAGCCGUUUGUUU...
                                                |||      |||||||
hsa-miR-495                          3'    UUCUUCACGUGGUA-CAAACAAA

Position 940-946 of PHF21A 3' UTR    5' ...GCAGCACGCGACCCAGGUGUGUG...
                                                     ||||||||||
hsa-miR-329                          3'     UUUCUCCAAUUGGUCCACACAA

Position 944-951 of PHF21A 3' UTR    5' ...CACGCGACCCAGGUGUGUGUGAA...
                                                        |||||||
hsa-miR-377                          3'     UGUUUUCAACGGAAACACACUA

Position 4502-4508 of PHF21A 3' UTR  5'    ...UUCAGUCUUGGUAGACUUGUAUU...
                                                 |||||       |||||||
hsa-miR-300                          3'    UCUCUCUCAGACGG----GAACAUAU
Position 4502-4508 of PHF21A 3' UTR  5'   ...UUCAGUCUUGGUAGACUUGUAUU...
                                                       |||||||
hsa-miR-381                          3'       UGUCUCUCGAACGGGAACAUAU
Position 4541-4547 of PHF21A 3' UTR  5'  ...AAAGUUUUUGUUUUUGUUUGUUU...
                                                         |||||||
hsa-miR-495                          3'      UUCUUCACGUGGUACAAACAAA
```

Supplementary Figure 17. The location of the binding sites of multiple cluster miRNAs in the 3'UTR of human *PHF21A(BHC80)* obtained from seed-based algorithm[1] of TargetScan (http://www.targetscan.org/) are shown.

Supplementary Figure 18

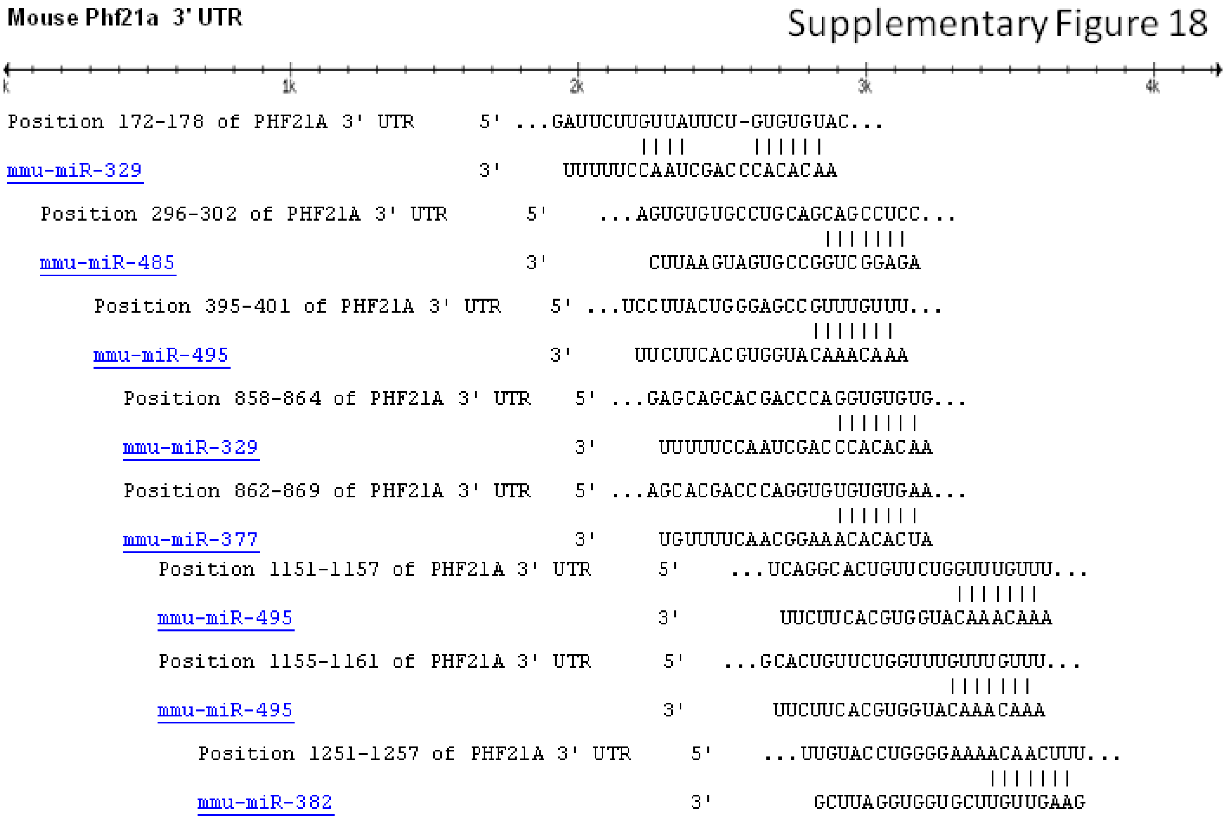

Supplementary Figure 18. The location of the binding sites of multiple cluster miRNAs in the 3'UTR of mouse *Phf21a* (*Bhc80*) obtained from seed-based algorithm[1] of TargetScan (http://www.targetscan.org/) are shown.

Human PUM2 3' UTR   Supplementary Figure 19

```
Position 55-62 of PUM2 3' UTR      5'  ...AAAAGAAUUUUUUUGUGUGUGAA...
                                                   |||||||
hsa-miR-377                        3'      UGUUUUCAACGGAAACACACUA

Position 82-88 of PUM2 3' UTR      5'  ...CAAAACACAACUCAACUAUGAAU...
                                                   ||||||
hsa-miR-376b                       3'      UUGUACCUAAAAGGAGAUACUA

Position 322-329 of PUM2 3' UTR    5'  ...UUUUUACCUUGUAAAGUCACAAA...
                                                   |||||||
hsa-miR-758                        3'      CCAAUCACCUGGUCCAGUGUUU

Position 545-551 of PUM2 3' UTR    5'  ...UUGUAUUUUGAUAAUUCACAAAC...
                                                   ||||||
hsa-miR-758                        3'      CCAAUCACCUGGUCCAGUGUUU

Position 684-690 of PUM2 3' UTR    5'  ...CAAUGUACAGAAAUAUUGUAUAU...
                                                   ||||||
hsa-miR-300                        3'      UCUCUCUCAGACGGGAACAUAU

Position 684-690 of PUM2 3' UTR    5'  ...CAAUGUACAGAAAUAUUGUAUAU...
                                                   ||||||
hsa-miR-381                        3'      UGUCUCUCGAACGGGAACAUAU

Position 1020-1026 of PUM2 3' UTR  5'  ...AAAUAUAUGCAUUGUGUGUGUAG...
                                                   ||||||
hsa-miR-329                        3'      UUUCUCCAAUUGGUCCACACAA

Position 1063-1069 of PUM2 3' UTR  5'  ...UUUGAAAGGAAAUCUUGUUUCAA...
                                                   ||||||
hsa-miR-494                        3'      CUCCAAAGGGCACAUACAAAGU

Position 1240-1246 of PUM2 3' UTR  5'  ...CCUGGCAAUAUAGUGUUGUAUAA...
                                                   ||||||
hsa-miR-300                        3'      UCUCUCUCAGACGGGAACAUAU

Position 1240-1246 of PUM2 3' UTR  5'  ...CCUGGCAAUAUAGUGUUGUAUAA...
                                                   ||||||
hsa-miR-381                        3'      UGUCUCUCGAACGGGAACAUAU

Position 1257-1263 of PUM2 3' UTR  5'  ...GUAUAAUGUAAAUUUAUUUCUCC...
                                                   |||||||
hsa-miR-539                        3'      UGUGUGGUUCCUAUUAAAGAGG

Position 1725-1731 of PUM2 3' UTR  5'  ...UUUUUAAUCCUUUGCCUAUGAAA...
                                                   ||||||
hsa-miR-376b                       3'      UUGUACCUAAAAGGAGAUACUA

Position 1815-1821 of PUM2 3' UTR  5'  ...GUUGAAGUCAGUAUUUUGUAUAA...
                                            ||||      ||||||
hsa-miR-300                        3'      UCUCUCUCAGACGGG-AACAUAU

Position 1815-1821 of PUM2 3' UTR  5'  ...GUUGAAGUCAGUAUUUUGUAUAA...
                                                   ||||||
hsa-miR-381                        3'      UGUCUCUCGAACGGGAACAUAU

Position 2180-2186 of PUM2 3' UTR  5'  ...GCAGCAACGCCUUGUGUUUGUUU...
                                                   |||||||
hsa-miR-495                        3'      UUCUUCACGUGGUACAAACAAA

Position 2183-2189 of PUM2 3' UTR  5'  ...GCAACGCCUUGUGUUUGUUUCAU...
                                                   ||||||
hsa-miR-494                        3'      CUCCAAAGGGCACAUACAAAGU

Position 2789-2795 of PUM2 3' UTR  5'  ...GUUAACUUCACUUCUUUGUAUAU...
                                                   ||||||
hsa-miR-381                        3'      UGUCUCUCGAACGGGAACAUAU

Position 2789-2795 of PUM2 3' UTR  5'  ...GUUAACUUCACUUCUUUGUAUAU...
                                                   ||||||
hsa-miR-300                        3'      UCUCUCUCAGACGGGAACAUAU

Position 2822-2829 of PUM2 3' UTR  5'  ...UUAGAAAAUGAAAAAUGUGUGAA...
                                                   |||||||
hsa-miR-377                        3'      UGUUUUCAACGGAAACACACUA
```

Supplementary Figure 19. The location of the binding sites of multiple cluster miRNAs in the 3'UTR of human *PUM2* obtained from seed-based algorithm[1] of TargetScan (http://www.targetscan.org/) are shown.

Mouse Pum2 3' UTR

Supplementary Figure 20

```
Position 74-80 of PUM2 3' UTR        5'  ...CAAAACACAAUUCAACUAUGAAU...
                                                         ||||||
mmu-miR-376b                         3'     UUCACCUACAAGGAGAUACUA

Position 343-350 of PUM2 3' UTR      5'  ...UUUAUACCUUGUAAAGUCACAAA...
                                                        |||||||
mmu-miR-758                          3'         AUCACCUGGUCCAGUGUUU

Position 478-485 of PUM2 3' UTR      5'  ...CUUAUUUUUUUCCUUUGUGUGAA...
                                                   |||||||||||
mmu-miR-377                          3'      UGUUUUCAACGGAAACACACUA

Position 566-572 of PUM2 3' UTR      5'  ...UUGUAUUUUGGUAAU--UCACAAAC...
                                                 |||      ||||||
mmu-miR-758                          3'          AUCACCUGGUCCAGUGUUU

Position 696-702 of PUM2 3' UTR      5'  ...UGACAAUGUACAGAAUUGUAUAU...
                                                       ||||||
mmu-miR-381                          3'     UGUCUCUCGAACGGGAACAUAU

Position 696-702 of PUM2 3' UTR      5'  ...UGACAAUGUACAGAAUUGUAUAU...
                                                        ||||||
mmu-miR-539-3p                       3'      UUUUUCUUUAAUAGGAACAUAC

Position 881-887 of PUM2 3' UTR      5'  ...GUUGAGGAAGUGUUG---GUUUGUUC...
                                                 ||||||      |||||||
mmu-miR-495                          3'          UUCUUCACGUGGUACAAACAAA

Position 1072-1078 of PUM2 3' UTR    5'  ...UUUGAAAGCAAAUCUUGUUUCAG...
                                                           ||||||
mmu-miR-494                          3'        CUCCAAAGGGCACAUACAAAGU

Position 1081-1088 of PUM2 3' UTR    5'  ...AAAUCUUGUUUCAGUGUUUGUUA...
                                                      |||||||
mmu-miR-495                          3'     UUCUUCACGUGGUACAAACAAA

Position 1262-1268 of PUM2 3' UTR    5'  ...GUAUAAUGUAAAUUUAUUUCUCC...
                                                      |||||||
mmu-miR-539-5p                       3'     UGUGUGGUUCCUAUUAAAGAGG

Position 1245-1251 of PUM2 3' UTR    5'  ...CCUGGCAAUAUAGUGUUGUAUAA...
                                                        ||||||
mmu-miR-381                          3'       UGUCUCUCGAACGGGAACAUAU

Position 1245-1251 of PUM2 3' UTR    5'  ...CCUGGCAAUAUAGUGUUGUAUAA...
                                                |||      ||||||
mmu-miR-539-3p                       3'     UUUUUCUUUAAUAGG-AACAUAC

Position 1765-1771 of PUM2 3' UTR    5'  ...UUGGAAAAAAUUGCUUUGUAUAU...
                                                        ||||||
mmu-miR-381                          3'       UGUCUCUCGAACGGGAACAUAU

Position 1765-1771 of PUM2 3' UTR    5'  ...UUGGAAAAAAUUGCU-UUGUAUAU...
                                                 |||||     ||||||
mmu-miR-539-3p                       3'     UUUUUCUUUAAUAGGAACAUAC

Position 1737-1743 of PUM2 3' UTR    5'  ...UUUUUAAUCCUUUGCCUAUGAAA...
                                                        ||||||
mmu-miR-376b                         3'     UUCACCUACAAGGAGAUACUA

Position 2109-2116 of PUM2 3' UTR    5'  ...CCACUAAGUCUGGCCAUGUUUCA...
                                                       |||||||
mmu-miR-494                          3'      CUCCAAAGGCACAUACAAAGU

Position 2754-2760 of PUM2 3' UTR    5'  ...AAUGUUAUCUUCACUUUGUAUAU...
                                                          ||||||
mmu-miR-381                          3'     UGUCUCUCGAACGGGAACAUAU

Position 2754-2760 of PUM2 3' UTR    5'  ...AAUGUUAUCUUCACUUUGUAUAU...
                                                           ||||||
mmu-miR-539-3p                       3'      UUUUUCUUUAAUAGGAACAUAC

Position 2786-2792 of PUM2 3' UTR    5'  ...AUUAGAAAAUGAAACGUGUGAAU...
                                                            ||||||
mmu-miR-377                          3'     UGUUUUCAACGGAAACACACUA
```

Supplementary Figure 20. The location of the binding sites of multiple cluster miRNAs in the 3'UTR of mouse *Pum2* obtained from seed-based algorithm[1] of TargetScan (http://www.targetscan.org/) are shown.

Supplementary Figure 21

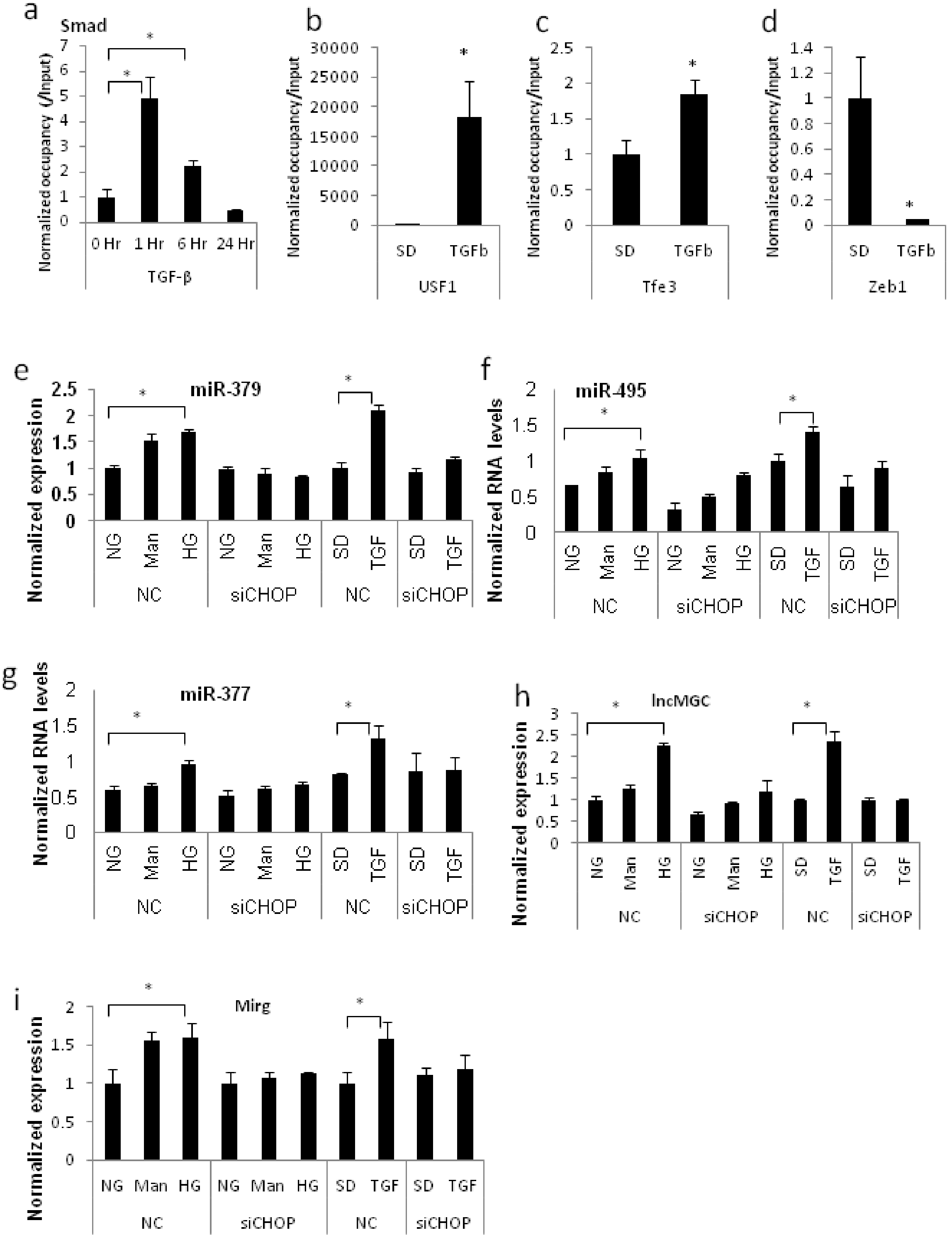

Supplementary Figure 21. Regulatory role of Smad, CHOP and E-box regulators (USF1, Tfe3 and Zeb1). (a-d) TGF-β1 increased the enrichment of Smad2/3 (a) at potential Smad binding elements (CAGA repeats), E-box activators USF1(b) and Tfe3 (c) but decreased E-box repressor Zeb1 (d) at the CHOP binding element and E-box region in MMC. (e-i) Effects of *Chop* siRNA on the expression of miR-379 (e), miR-495 (f), miR-377 (g), lnc-MGC (h) and Mirg (i). *Chop* siRNA inhibited the induction of these RNAs in MMC treated with TGF-β1 or HG but did not affect basal levels. Respective control treatments are NG and mannitol for HG, and SD for TGF-β1. Results are mean + SE in triplicate PCRs of three independent culture experiments. *, $P<0.05$.

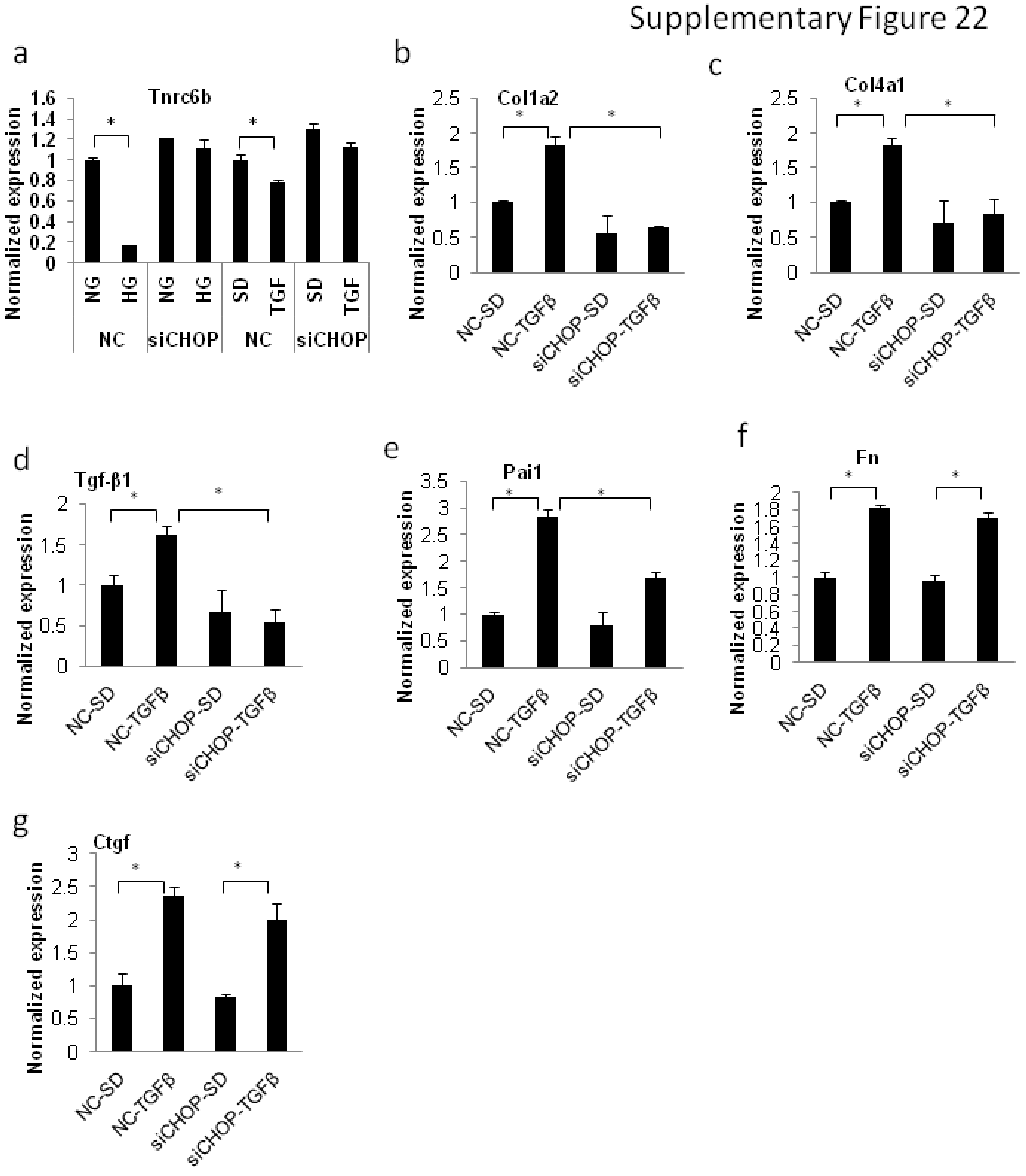

Supplementary Figure 22. Effects of *Chop* siRNA on the expression of potential targets of the cluster miRNAs in MMC treated with TGF-β1 or HG. (a) *Chop* siRNA reverses the inhibition of *Tnrc6* expression in MMC treated with TGF-β1 or HG. NC refers to negative control siRNA (b-g) Effects of *Chop* siRNA on the expression of candidate pro-fibrotic genes. *Chop* siRNA significantly inhibited the induction of *Col1a2* (b), *Col4a1* (c), *Tgf-β1* (d), *PAI1* (e) but not *FN1* (f) or *Ctgf* (g) in MMC treated with TGF-β1. SD is serum-depleted control. No significant effect of *Chop* siRNA on basal expression of these profibrotic genes was detected. Results are mean + SE in triplicate PCRs of three independent culture experiments. *, P<0.05.

Supplementary Figure 23

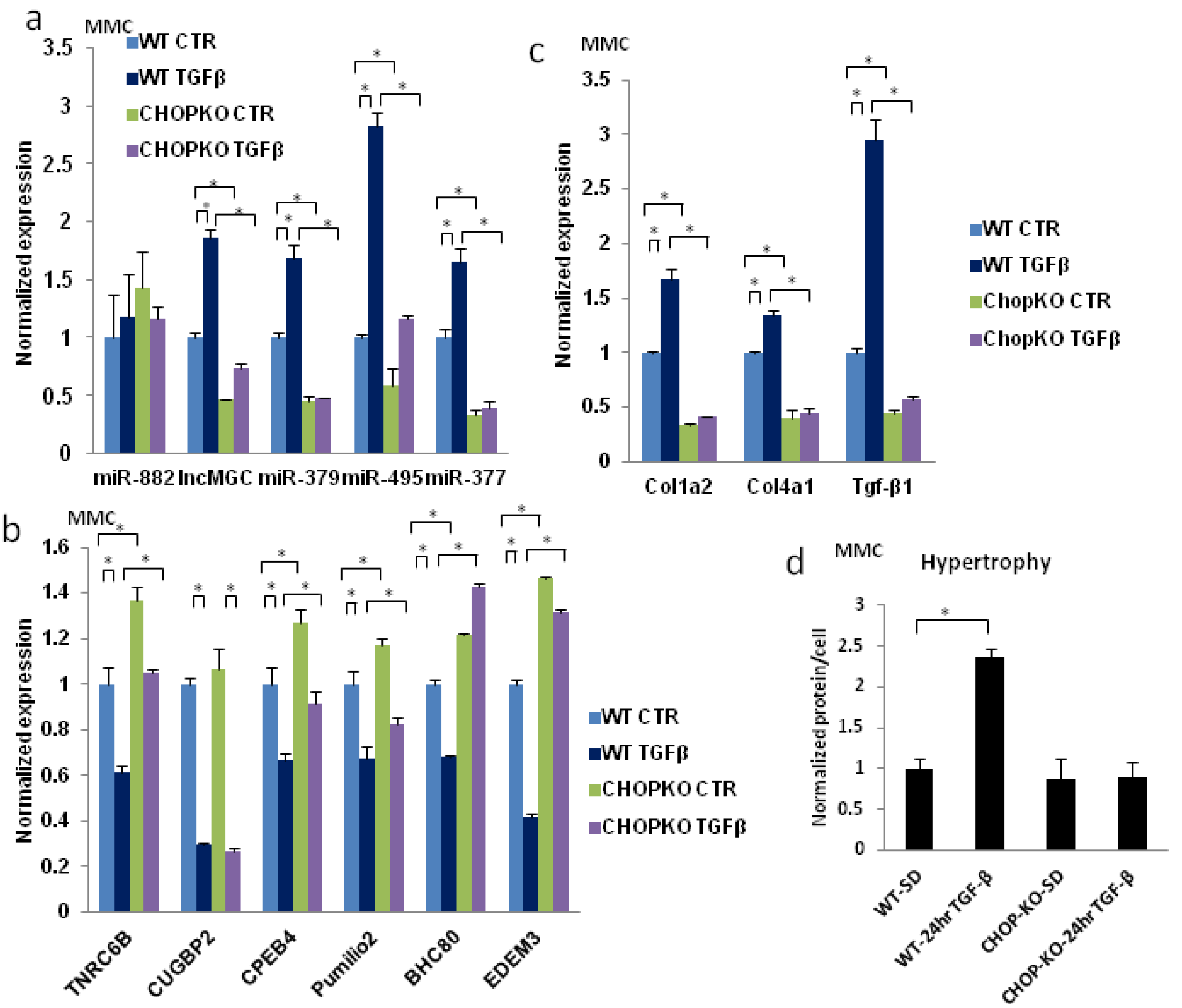

Supplementary Figure 23. lnc-MGC, miR-379 cluster miRNAs and potential targets in MMC treated with TGF-β1 and in MMC from *Chop* -KO mice. (a) Induction of lnc-MGC and candidate miR-379 cluster miRNAs in MMC treated with TGF-β1 was attenuated in MMC from *Chop* -KO mice. (b) Decrease of potential targets of miR-379 cluster in MMC treated with TGF-β1 was attenuated in MMC from *Chop* -KO mice (except *Cugbp2*). (c) Induction of pro-fibrotic genes in MMC from WT mice treated with TGF-β1 was attenuated in MMC from *Chop* -KO mice. Results are mean + SE in triplicate PCRs of three-four independent culture experiments. *, P<0.05. (d) TGF-β1 induced cellular hypertrophy is ameliorated in MMC from *Chop* -KO mice. Although significant increase of hypertrophy was observed in MMC from WT mice treated with TGF-β1 relative to SD control, no change was observed in MMC from *Chop* -KO mice. For TGF-β1 treatment, MMC were serum-depleted (SD) and treated with or without 10 ng/ml TGF-β1 for 24 hours. Results are mean + SE in three independent experiments. *, P<0.05.

Supplementary Figure 24

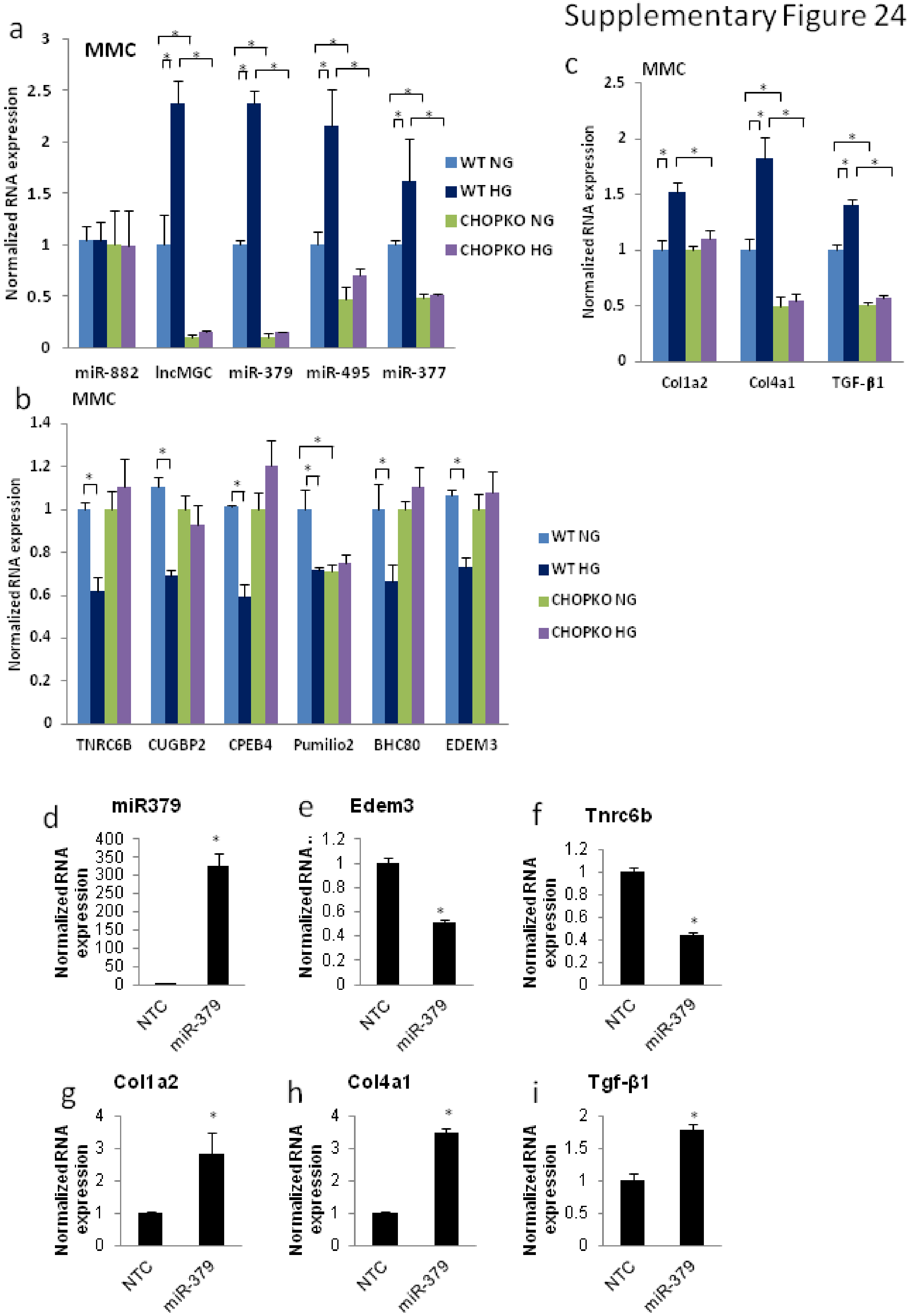

Supplementary Figure 24. **lnc-MGC, miR-379 cluster miRNAs and potential targets in MMC treated with HG and in MMC from *Chop* -KO mice.** (a) Induction of lnc-MGC and miR-379 cluster miRNAs in MMC treated with HG was attenuated in MMC from *Chop* -KO mice. (b) Decrease of potential targets of miR-379 cluster in MMC treated with HG was attenuated in MMC from *Chop* -KO mice. (c) Induction of pro-fibrotic genes in MMC from WT mice treated with HG was attenuated in MMC from *Chop* -KO mice. For HG treatment, MMC were cultured in medium containing HG (25mM) and serum for 72 hours while control cells were cultured for the same time period in NG (5.5 mM). (d-i) The effects of miR-379 overexpression (with mimics) on the expression of targets and profibrotic genes in *Chop*-KO MMC. Significant increase of miR-379 was confirmed after transfection on MMC with miR-379 mimics relative to NTC (d). Significant decrease of targets, *Edem3* and *Tnrc6b* (e & f) and significant increase of profibrotic genes, *Col1a2* (g), *Col4a1* (h) and *Tgf-β1* (i) were detected in *Chop* -KO MMC transfected with miR-379 mimic. Results are mean + SE in triplicate PCRs of three independent culture experiments. *, P<0.05.

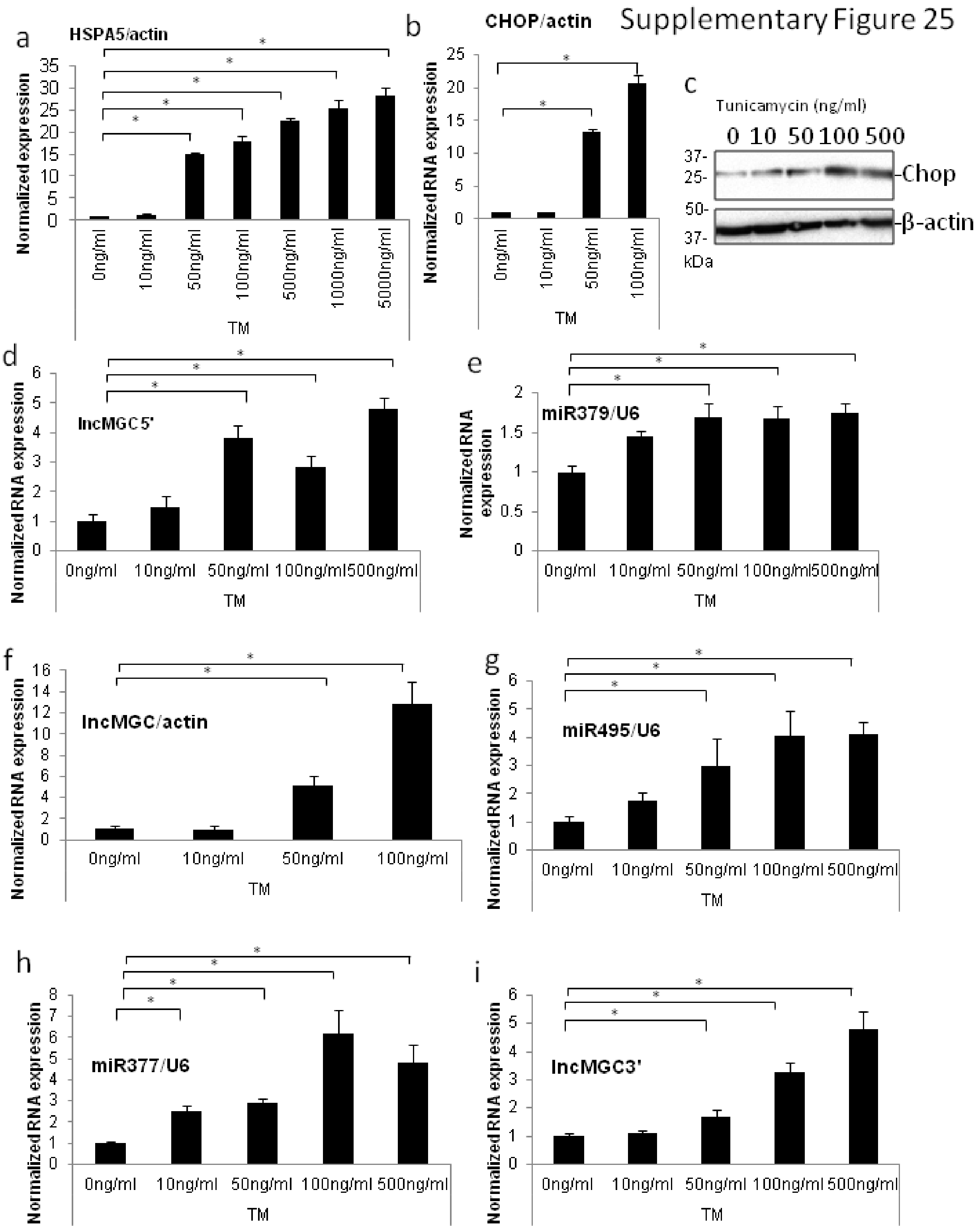
Supplementary Figure 25
a
HSPA5/actin
Normalized expression
0ng/ml
10ng/ml
50ng/ml
100ng/ml
500ng/ml
1000ng/ml
5000ng/ml
TM
b
CHOP/actin
Normalized RNA expression
0ng/ml
10ng/ml
50ng/ml
100ng/ml
TM
c
Tunicamycin (ng/ml)
0 10 50 100 500
-Chop
-β-actin
kDa
d
lncMGC 5'
Normalized RNA expression
0ng/ml 10ng/ml 50ng/ml 100ng/ml 500ng/ml
TM
e
miR379/U6
Normalized RNA expression
0ng/ml 10ng/ml 50ng/ml 100ng/ml 500ng/ml
TM
f
lncMGC/actin
Normalized RNA expression
0ng/ml 10ng/ml 50ng/ml 100ng/ml
TM
g
miR495/U6
Normalized RNA expression
0ng/ml 10ng/ml 50ng/ml 100ng/ml 500ng/ml
TM
h
miR377/U6
Normalized RNA expression
0ng/ml 10ng/ml 50ng/ml 100ng/ml 500ng/ml
TM
i
lncMGC3'
Normalized RNA expression
0ng/ml 10ng/ml 50ng/ml 100ng/ml 500ng/ml
TM

Supplementary Figure 25. Tunicamycin (TM) induces the expression of miRNAs in the miR-379 cluster in MMC. (a) To optimize TM treatment conditions in MMC to induce ER stress, HSPA5 was used as indicator of ER stress in response to several doses of TM. 50 ng/ml was the minimum dose required to induce significant expression of HSPA5 in MMC. (b&c) The same dose 50 mg/ml was optimal for induction of *Chop* mRNA (b) and protein (uncropped scans are shown in Supplementary Figure 38) (c) in MMC. Wider (uncropped) scans of blots are shown in Supplementary Figure 38. (d-i) The expression of lnc-MGC (d, 5'; f, middle; i, 3') and miRNAs, miR-379 (e), miR-495 (g) and miR-377 (h) in the cluster were increased by TM (~50ng/ml). Results are mean + SE in triplicate PCRs of three independent culture experiments. *, $P<0.05$.

Supplementary Figure 26

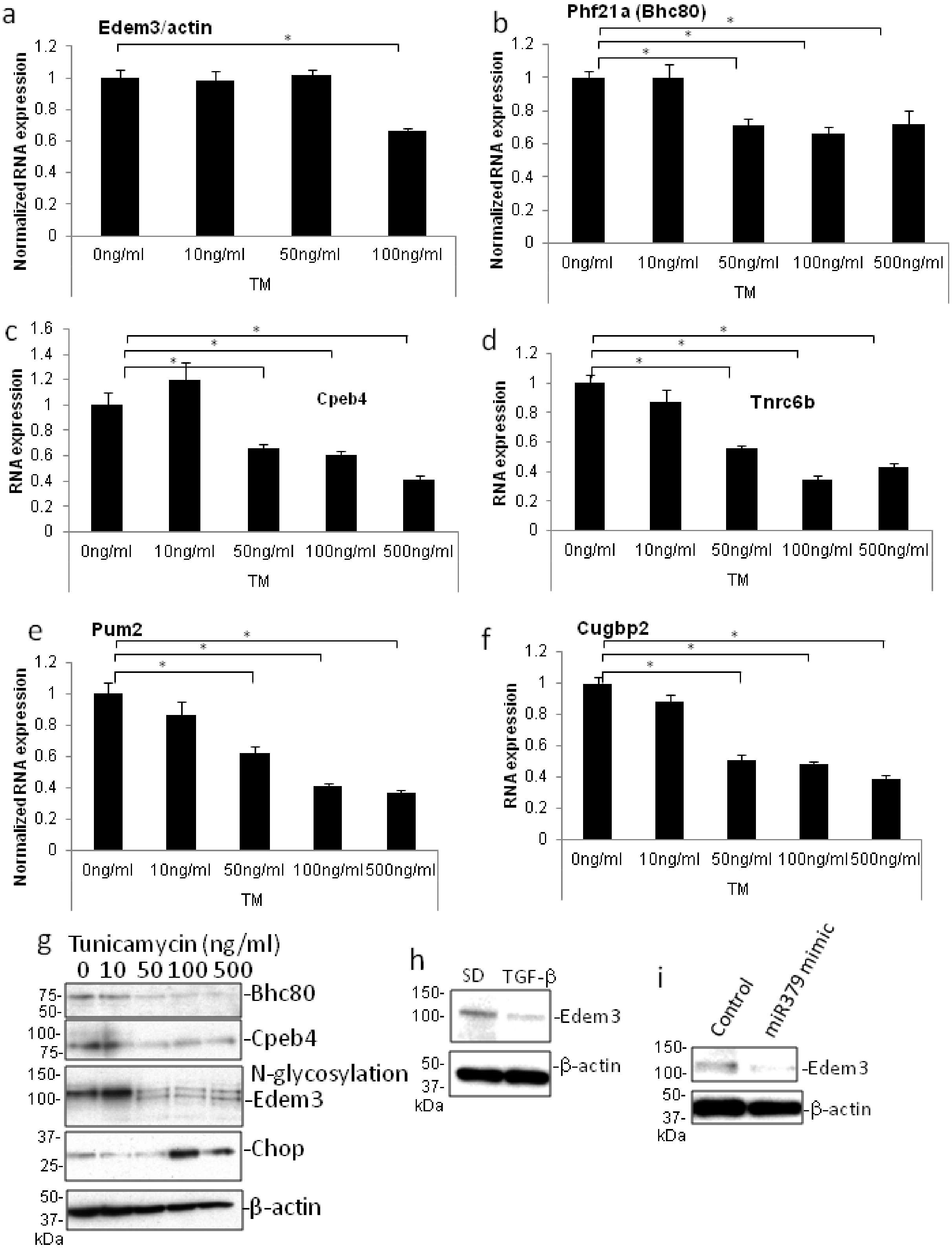

Supplementary Figure 26. Tunicamycin (TM) reduces targets of miRNA cluster in MMC. (a) A target of miR-379, *Edem3* mRNA was decreased by TM. (b-g) Five other indicated targets of the miR-379 cluster were also down regulated by TM in MMC (mRNA and protein). Results are mean + SE in triplicate PCRs of three independent culture experiments. *, P<0.05. (g) Western blots showing the decrease of Edem3, Bhc60, Cpeb4 proteins by TM in MMC. Faster migrating isoform of Edem3 protein was detected in TM-treated cells by western blot while no such isoform was detected in MMC treated with TGF-β1 (h) or transfected with miR-379 mimic relative to control oligo (i). Wider (uncropped) scans of blots are shown in Supplementary Figure 38 and 39.

Supplementary Figure 27

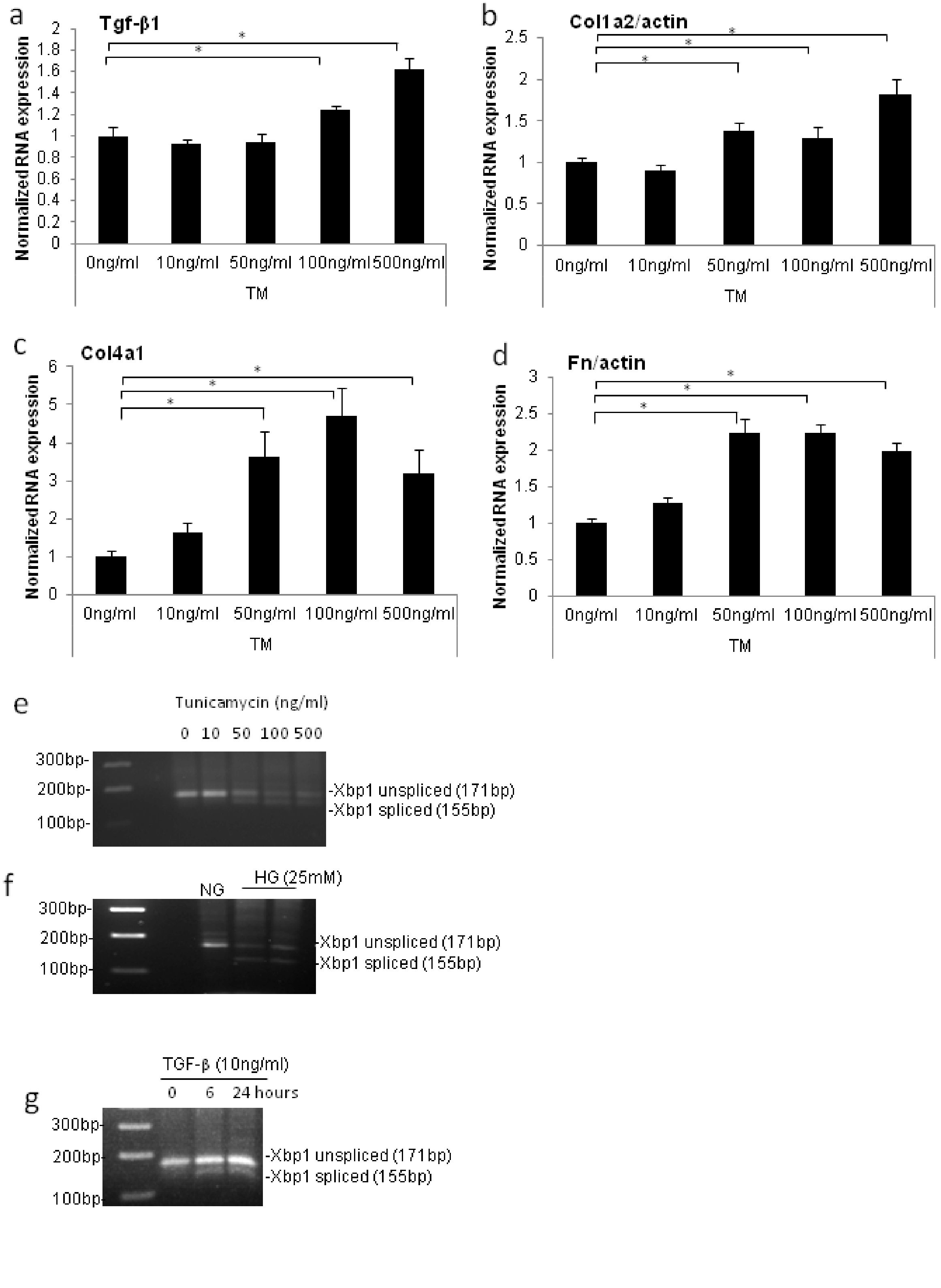

Supplementary Figure 27. The expression of pro-fibrotic genes in MMC treated with TM and *Xbp1* splicing in MMC treated with TM, HG or TGF-β1. (a-d) The expression of pro-fibrotic genes, *Tgf-β1* (a), *Col1a2* (b), *Col4a1* (c) and *Fn* (d) was also increased by TM in MMC. Results are mean + SE in triplicate PCRs of three independent culture experiments. *, P<0.05. (e-h) *Xbp1* splicing was monitored by PCR. ER stress-induced spliced form (shorter form) was detected in MMC treated with TM in a dose-dependent manner (e), with HG (25mM) for 72 hours (f) and with 10ng/ml TGF-β1 for 6 and 24 hours (g).

Supplementary Figure 28

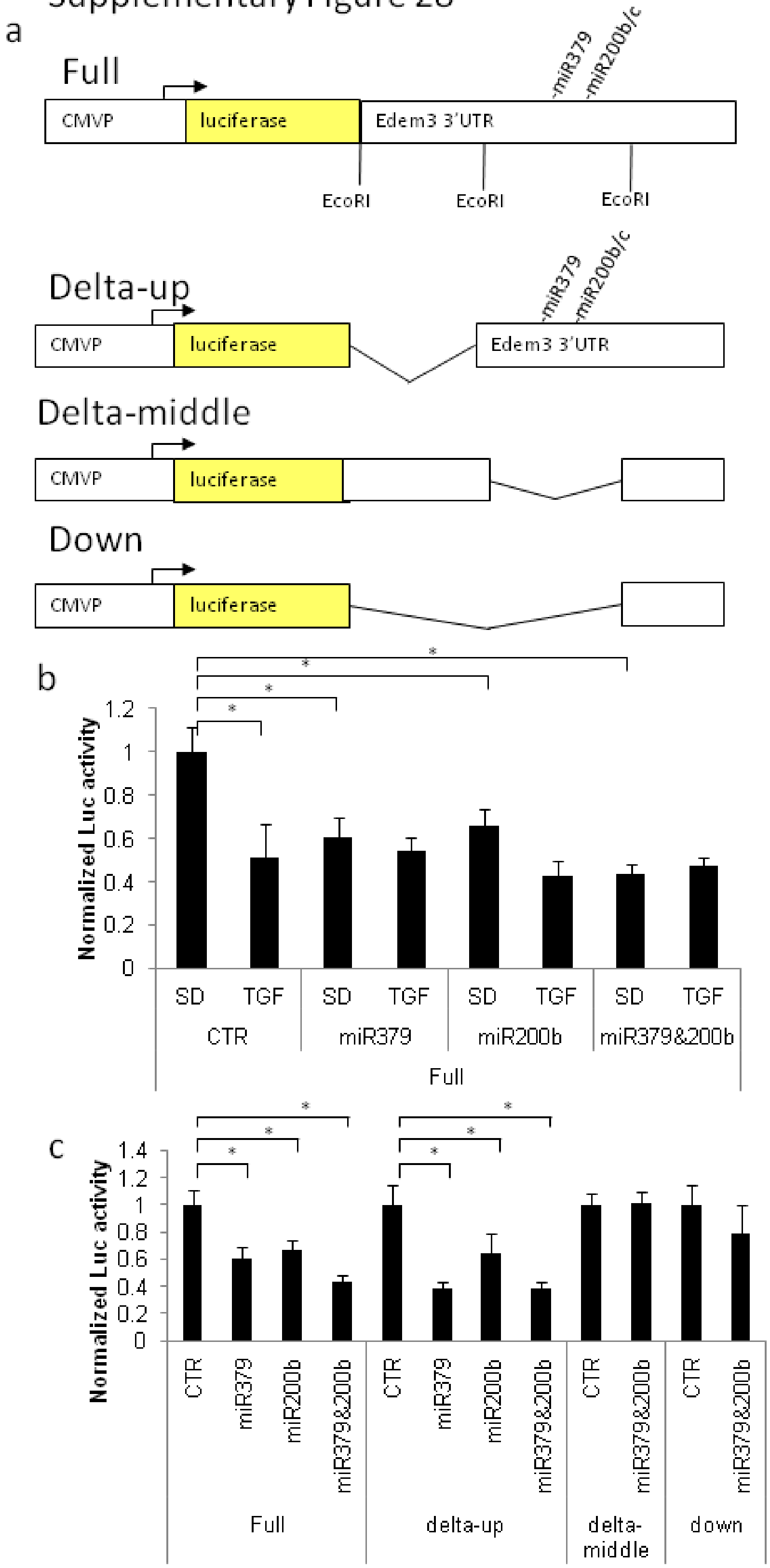

Supplementary Figure 28. ***Edem3* 3'UTR is a *bonafide* target of miR-379 (and miR-200b).** (a) Schematic structures of the luciferase reporter of *Edem3* 3'UTR. Potential miR-200b/c target site was also found in the 3'UTR of *Edem3*. Deletion mutants (delta-up, delta-middle and down) were constructed. Full and delta-up include miR-379 and miR-200b/c sites but these sites are deleted in delta-middle and down. (b) MMC was transfected with reporter plasmids with 3'UTR of *Edem3* and miR-379 or miR-200b mimic. Full length reporter responded (significant decrease of luciferase activity) to TGF-β1 and also to miR-379 and miR-200b. (c). Full and delta-up (including miR-379 and miR-200b/c sites) significantly responded to miR-379 and miR-200b but others (Delta-middle and down without miR-379 and miR-200b/c sites) did not. Results are mean + SE from triplicate reads of four independent cultures. *, $P<0.05$.

Supplementary Figure 29

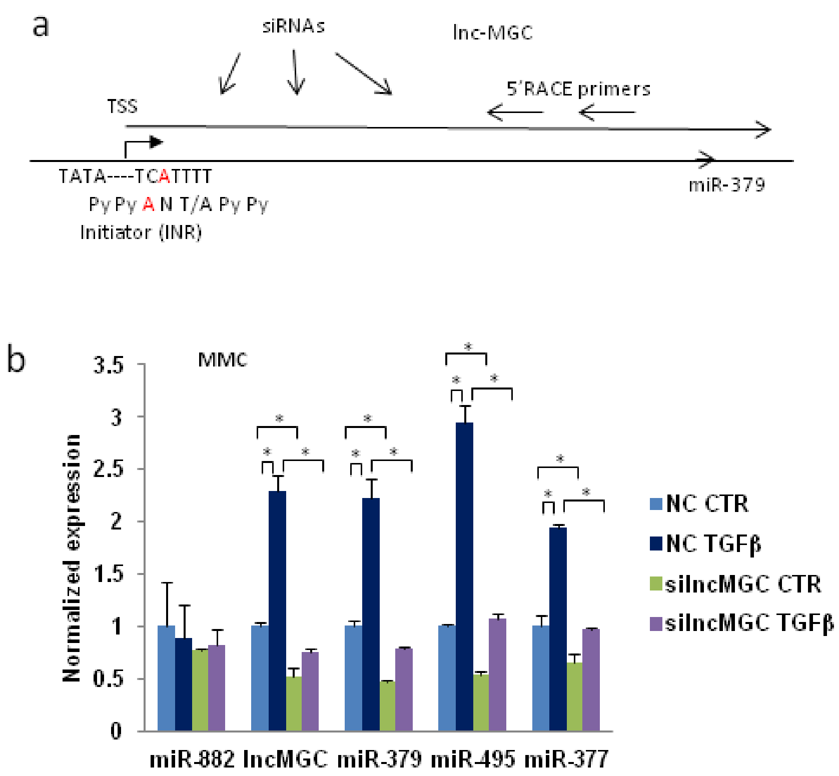

Supplementary Figure 29. siRNAs against lnc-MGC inhibited expression of miR-379 cluster miRNAs. (a) Three siRNAs were designed to target the upstream region of lnc-MGC. (b) The mixture of these siRNAs inhibited TGF-β1-induced increase in the expression of not only linc-MGC but also miR-379, miR-495 and miR-377 in MMC compared to negative control siRNA (NC). Results are mean + SE in triplicate PCRs of three independent culture experiments. *, $P<0.05$.

Supplementary Figure 30

a Gapmers design

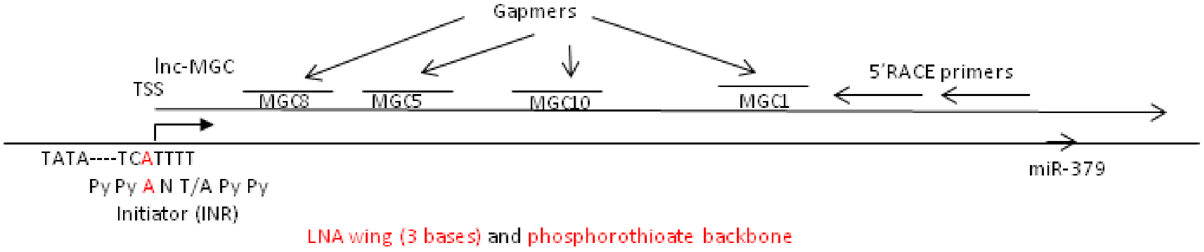

b

Mouse miR-379 upstream

Py Py A N T/A Py Py (INR)

TATAGTCAGCACAGTGGTTCATTTTTCTGAGTTAGTGTGGCCTTCATCTGGTAATGTACTACCTGAGGGGGGAGGTGCCG
CCTCTCTTTCAGCACCGTGCAACCATTCAAGGAGGGTGTGTTGTTCACCACATCTGCTTCCCACTGCCAAATCAGGCCTCA
GAAAAGCTTTCTGGAAGTGACGCCAGCTTCAGGGACAAGGCCCAAGTTTCTAGGGGTCAACACC-miR-379---

Mouse target CTTCCCACTGCCAAAT

MGC10 (ATTTGGCAGTGGGAAG) LNA & full PS

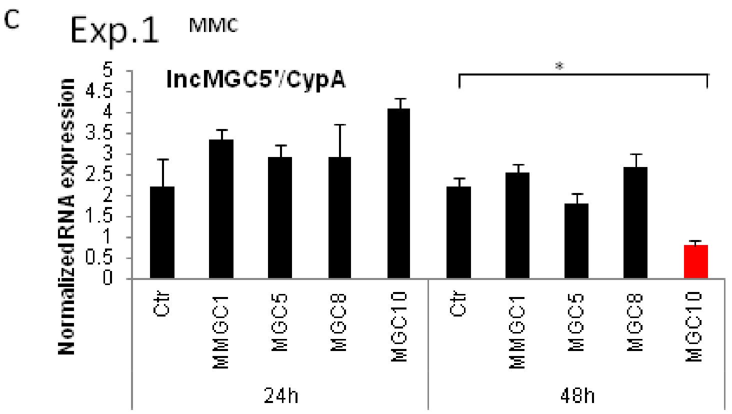

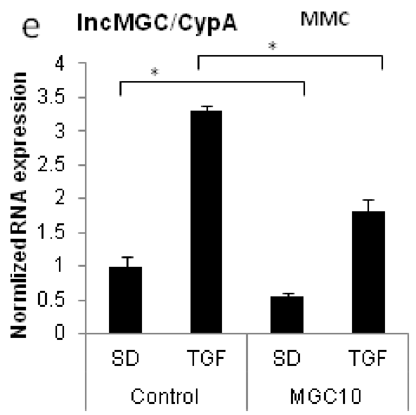

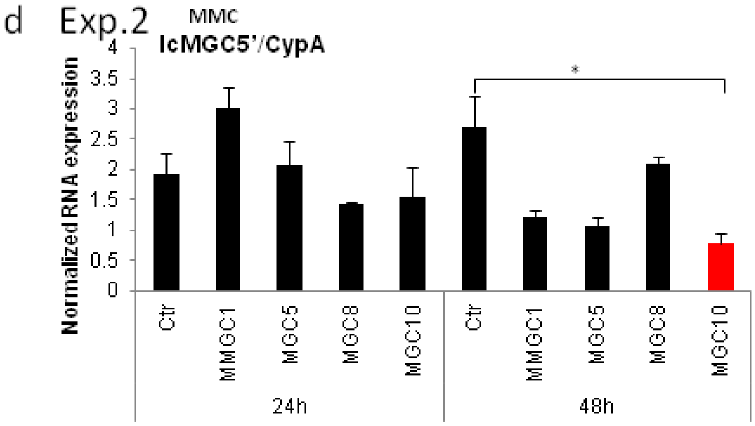

Supplementary Figure 30. Design and Efficacy of GapmeRs targeting lnc-MGC. LNA-modified GapmeR inhibits lnc-MGC in MMC in vitro. (a) Four GapmeRs (MGC 1,5, 8 and 10) were designed to target the upstream sequence of lnc-MGC. (b) Location of mouse lnc-MGC target sequence (red font) and chemistry of MGC10 (the best design based on the effects on expression of lnc-MGC in MMC in vitro). First and last three bases were replaced with LNA (green font) and others were DNA. The backbone was fully phosphorothioated. The Control GapmeR oligo had similar chemistry (modification) as MGC10, (our specific lncRNA targeting GapmeR), and also with no homology to any known mRNA, miRNA, or lncRNA in mouse, rat and human. (c&d) Out of the four oligos tested, MGC10 was the most effective and consistent inhibitor of lnc-MGC in MMC in vitro at 48 hours in two independent experiments. (e) MGC10 inhibited the expression of lnc-MGC even after treatment of TGF-β1 in MMC. Results are mean + SE in triplicate PCRs of three independent culture experiments. *, $P<0.05$.

Supplementary Figure 31

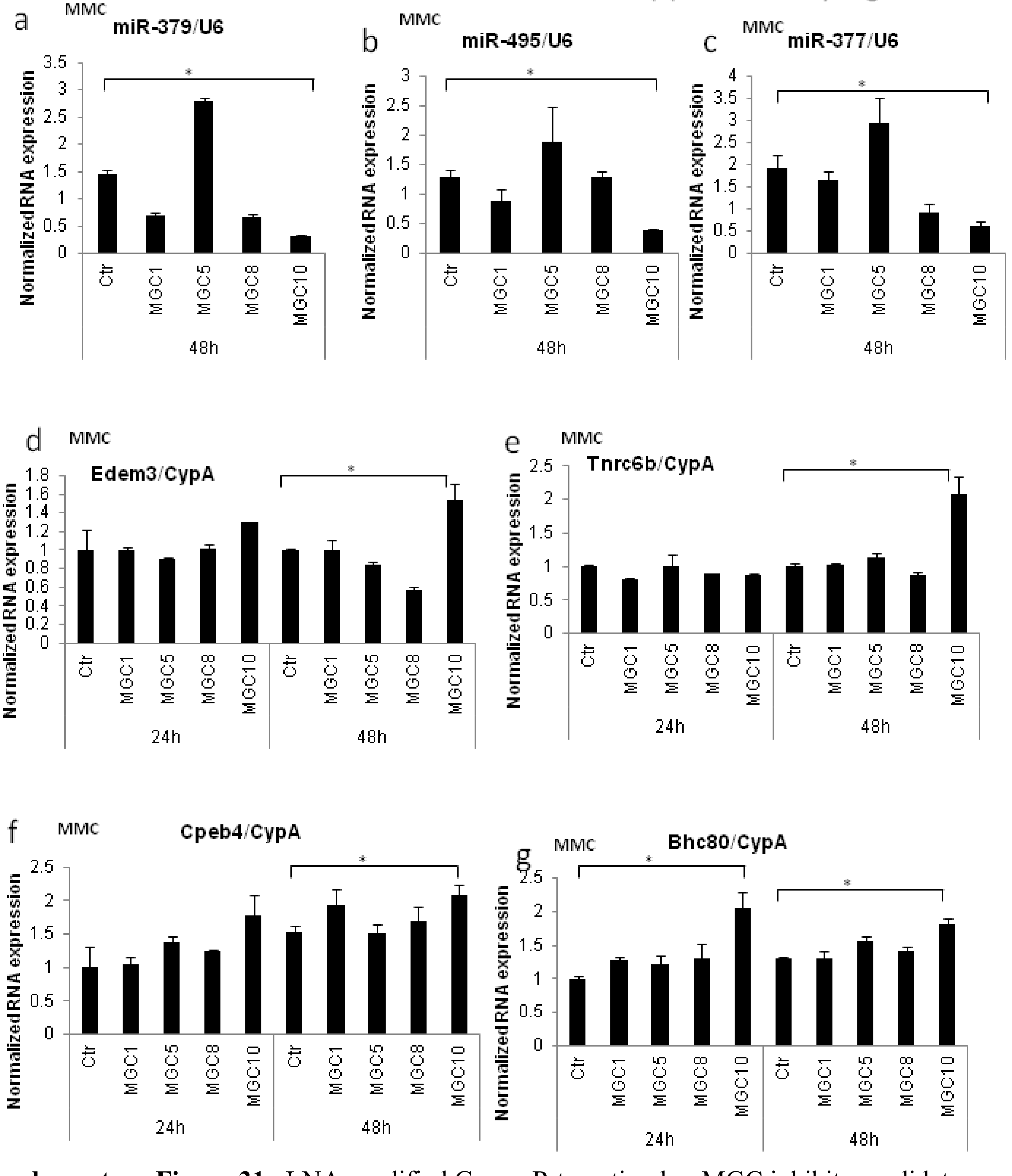

Supplementary Figure 31. LNA-modified GapmeR targeting lnc-MGC inhibits candidate miRNAs in the miR-379 cluster in MMC in vitro compared to control oligo (Ctr). (a-c) miR-379 cluster miRNAs, miR-379 (a), miR-495 (b) and miR-377 (c) were significantly downregulated by MGC10. (d-g) Several target genes of key cluster miRNAs, *Edem3* (d), *Trnc6b* (e), *Cpeb4* (f) and *Bhc80* (g) were upregulated by MGC10 as related to reduction of the miR-379 cluster miRNAs. Results are mean + SE in triplicate PCRs of three independent culture experiments. *, P<0.05.

Supplementary Figure 32

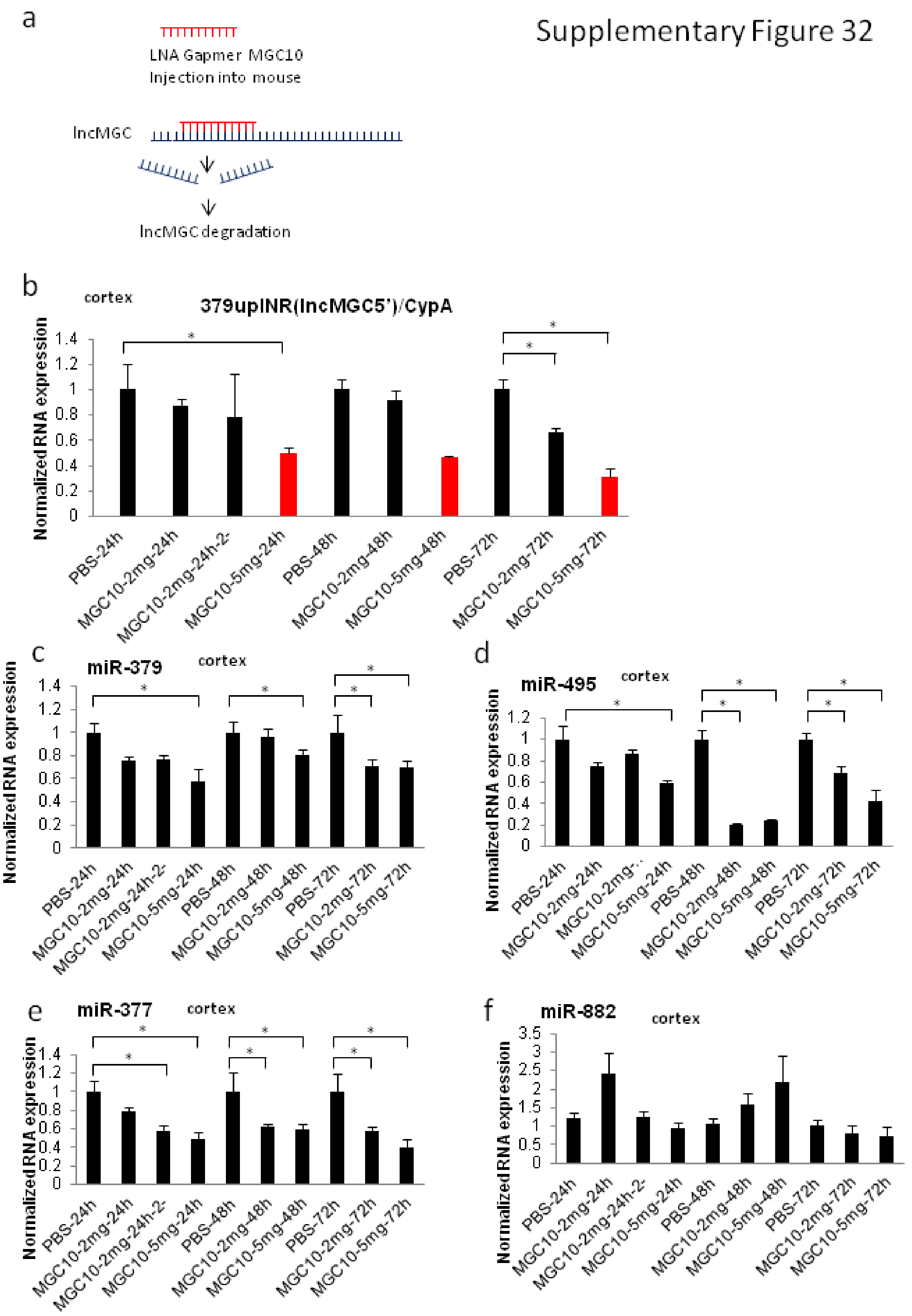

Supplementary Figure 32. MGC10 inhibits miR-379 cluster miRNAs in the mouse kidney in vivo. (a-f) Subcutaneous injection of 5mg/kg MGC10 consistently inhibited the expression of lnc-MGC in kidney cortex of normal mice at 24-72 hours after injection. Three mice in each group. The expression of lnc-MGC (b) and miRNAs in the miR-379 cluster, miR-379 (c), miR-495 (d), miR-377 (e) were inhibited by subcutaneous injection of 5mg/kg MGC10, while miR-882 outside of the cluster was not (f). Three mice were injected for each condition and each time point. Gene expression quantified in cortical samples. Results are mean + SE in triplicate PCRs from each mouse, *, $P<0.05$.

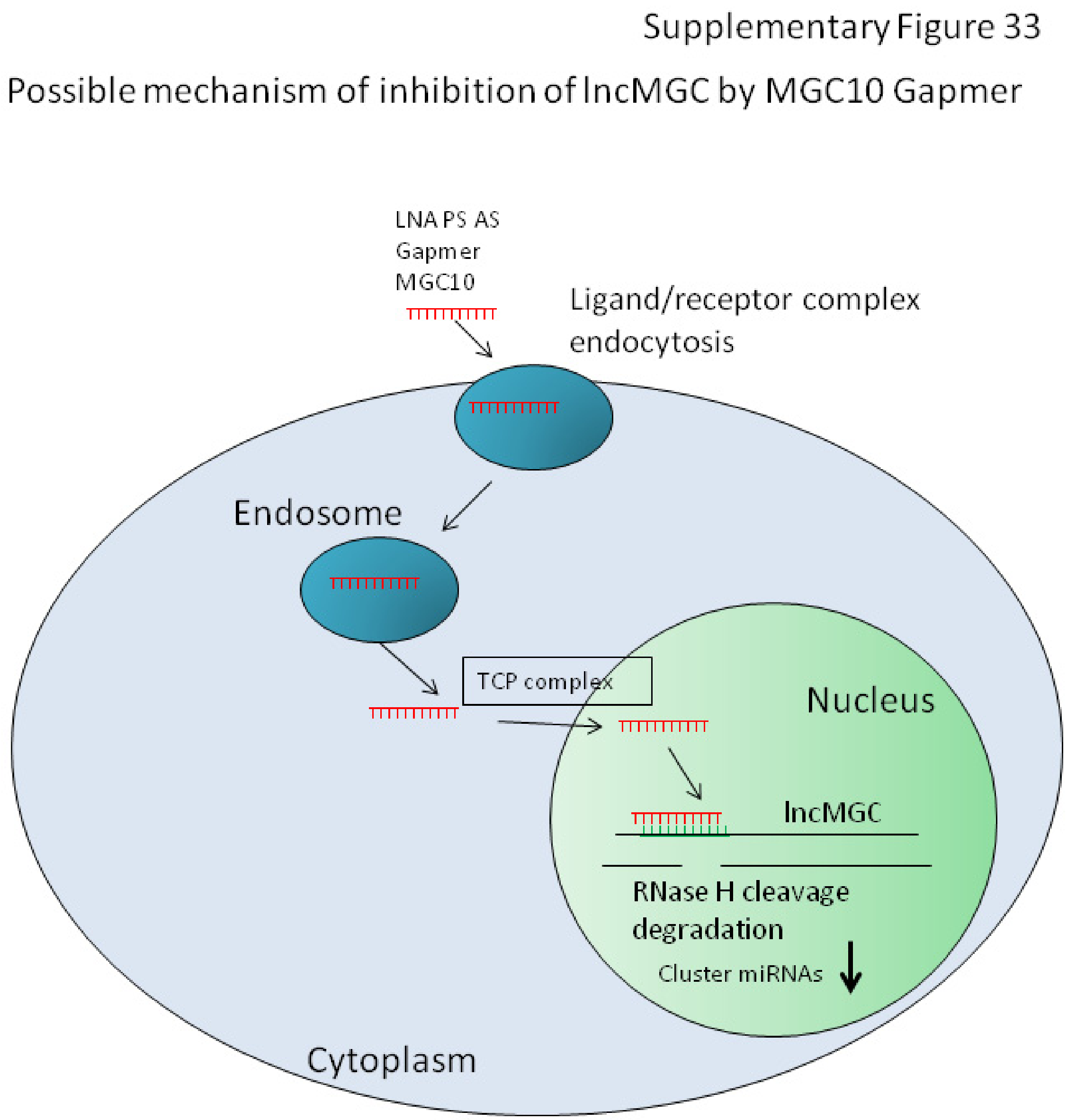

Supplementary Figure 33. Scheme depicting the potential mechanism of inhibition of nuclear lnc-MGC by MGC10. Phosphorothioated oligonucleotides can be transported into the nucleus by a protein complex (TCP1 complex) [2]. Because MGC10 is fully phosphorothioated, it may be efficiently transported into nucleus and thereby cleave lnc-MGC RNA and also suppress the expression of miR-379 cluster (hosted within lnc-MGC).

Supplementary Figure 34

a

5 mice (non-diabetic control)

17 mice (diabetes)
Five injections daily (50 mg/kg)
Confirm diabetes (blood glucose)

5 mice (no oligo) | 6 mice (control oligo injection) | 6 mice (MGC10 injection, 5mg/kg/week)

5 weeks

Blood glucose
Serum profile

Kidney RNA (non-coding RNAs, microRNAs, target RNAs, profibrotic genes or ER stress-related genes)
Kidney protein

Kidney sections, PAS staining (glomerular hypertrophy, mesangial expansion, ER stress-related proteins)

b Serum profile

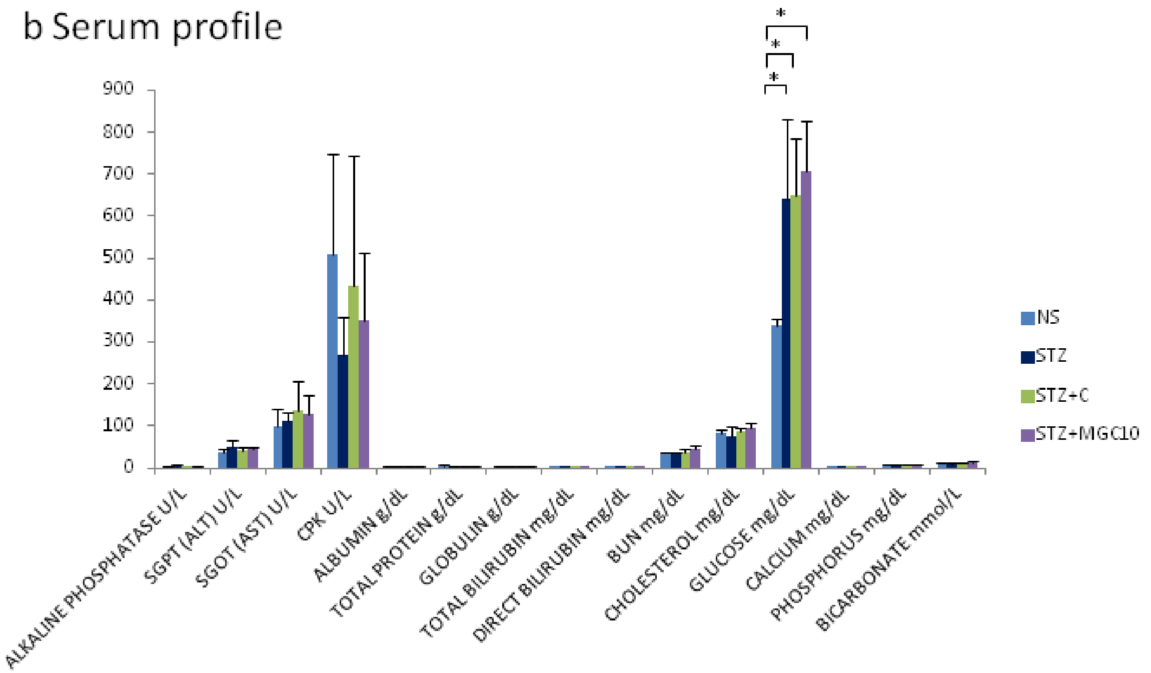

Supplementary Figure 34. (a) **The experimental scheme for the in vivo GapmeR injection experiments in normal and diabetic mice**. Five non-diabetic mice, five diabetic mice without injection, 6 diabetic mice injected with control oligos and 6 diabetic mice injected with MGC10 were used. (b) Serum profile of mice used in this study. MGC10 injection did not have significant effects on parameters of liver or kidney toxicity. Significant increase of blood glucose was detected in all diabetic (STZ, STZ+C, STZ+MGC10) groups compared to non-diabetic group (NS). MGC10 injection did not change blood glucose levels, either. These results suggest a good safety profile for injected LNA modified GapmeRs in mice models of early DN.

Supplementary Figure 35

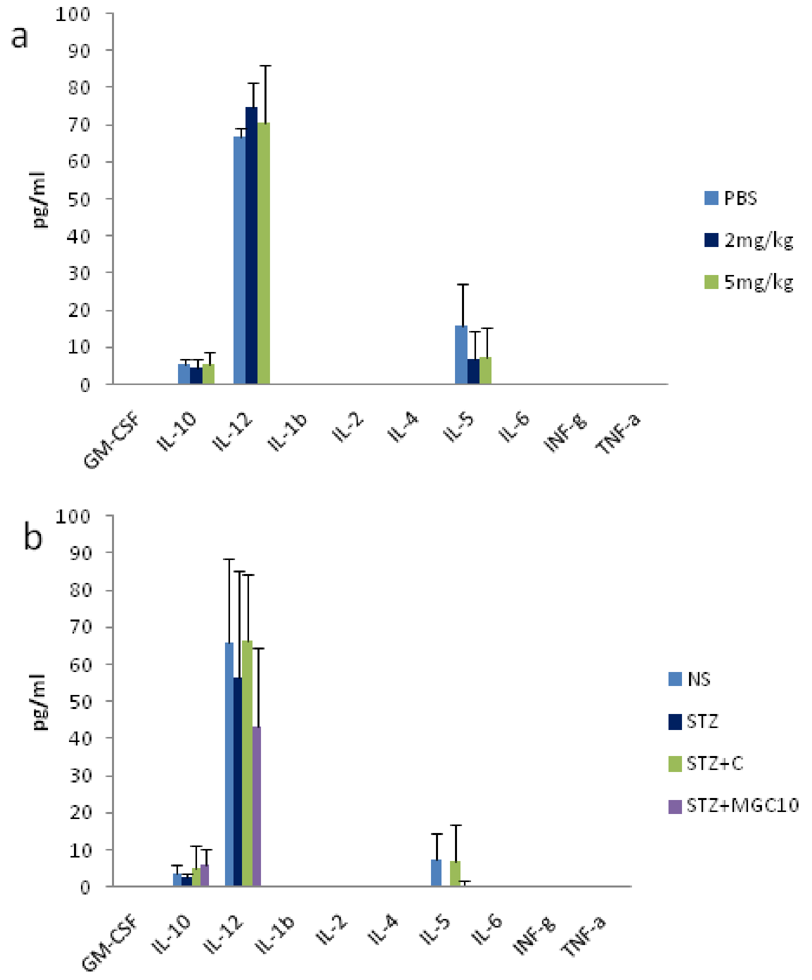

Supplementary Figure 35. Cytokine profiles in sera from GapmeR injected mice. (a) Cytokine profiles in sera from GapmeR injected nondiabetic mice. No significant changes in the levels of 10 cytokines (GM-CSF, INF-g, IL-1b, IL-2, IL-4, IL-5, IL-6, IL-10, IL-12(P40/P70), TNF-a) were observed between mice injected PBS , 2mg/kg MGC10 or 5mg/kg MGC10. (b) Cytokine profiles in sera from GapmeR injected STZ-diabetic mice. No significant difference was detected in the levels of 10 cytokines (GM-CSF, INF-g, IL-1b, IL-2, IL-4, IL-5, IL-6, IL-10, IL-12,(P40/P70), TNF-a) between NS, STZ, STZ+C, STZ+MGC10 groups.

Supplementary Figure 36

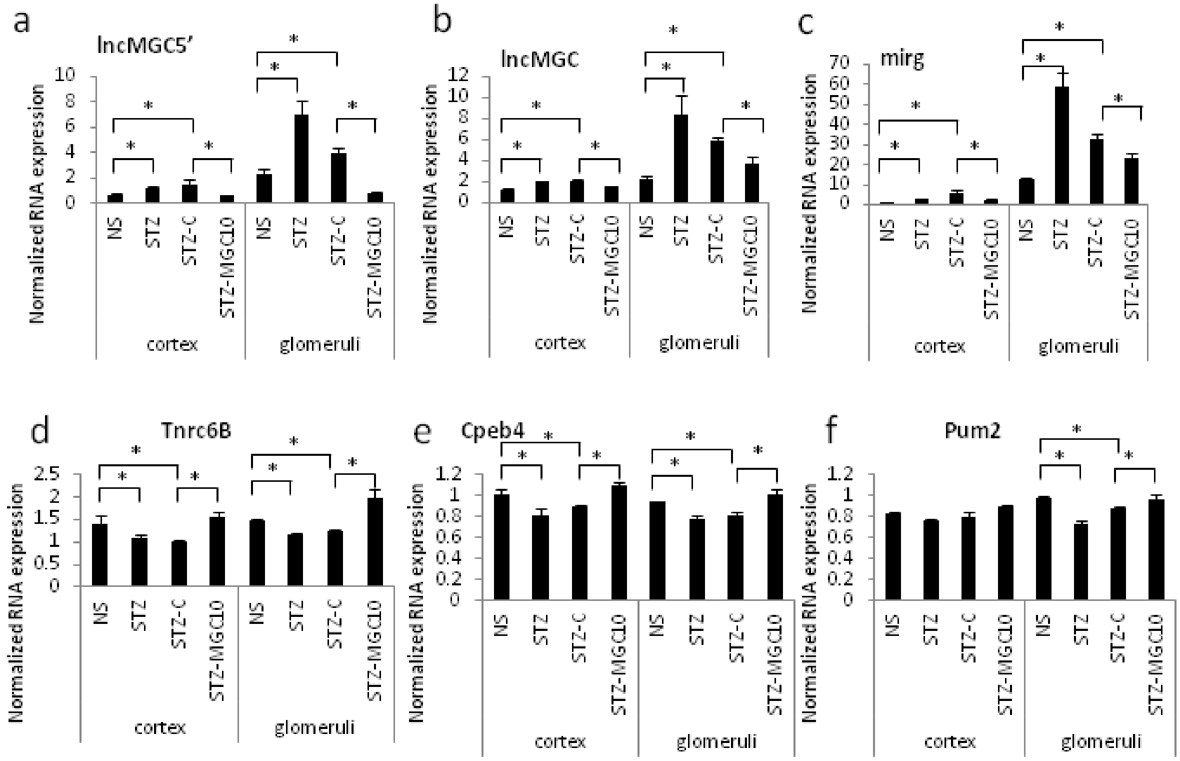

Supplementary Figure 36. Effects of MGC10 on lnc-MGC, cluster miRNAs, potential targets and profibrotic genes in kidney cortex and glomeruli from STZ diabetic mice. (a-c) Increase of lnc-MGC, lncMGC5', upstream of lnc-MGC (a), middle region lnc-MGC (b), lnc-MGC3' (mirg) (c), in kidney cortex and glomeruli from STZ diabetic mice and their inhibition by injection of MGC10. (d-f) Decrease of miR-379 cluster targets, *Tnrc6b* (d), *Cpeb4* (e), *Pumilio2* (only in glomeruli) (f) in kidney cortex and glomeruli from STZ diabetic mice and their restoration by injection of MGC10. Results are mean + SE in triplicate PCRs from each mouse. *, $P<0.05$.

Supplementary Figure 37

a

Human miR-379 upstream

Py Py A N T/A Py Py (INR)

GTTGACCACATAATGGCCAAAGCATGGTCAGTATTGTACATTTTGCCTTGTCGCAGTCTTTCCAAGTTGACATGGCCTTCCTGGAGGAATTACCACTTAGGGTAG
AGGCACCCCTTCCCCCATCAATGCCACTGCCCCACATTGGAGGAGGGGTTGTTTATGTTCACCATGTGCCTGCTTCCAATGCCAAATCCAGCCTCAGAAAGCTTTC
TGGAAGTGACGCCAACTTCAGGGGCAAGGCCCTGGTTCTGGGGTCAGCACCATTCCGTGGTTCCTGAAGAGATGGTAGACTATGGAA-miR-379----

Human CTTCCAATGCCAAATC
Mouse target CTTCCCACTGCCAAAT

HMGC10 (GATTTGGCATTGGAAG) LNA & full PS

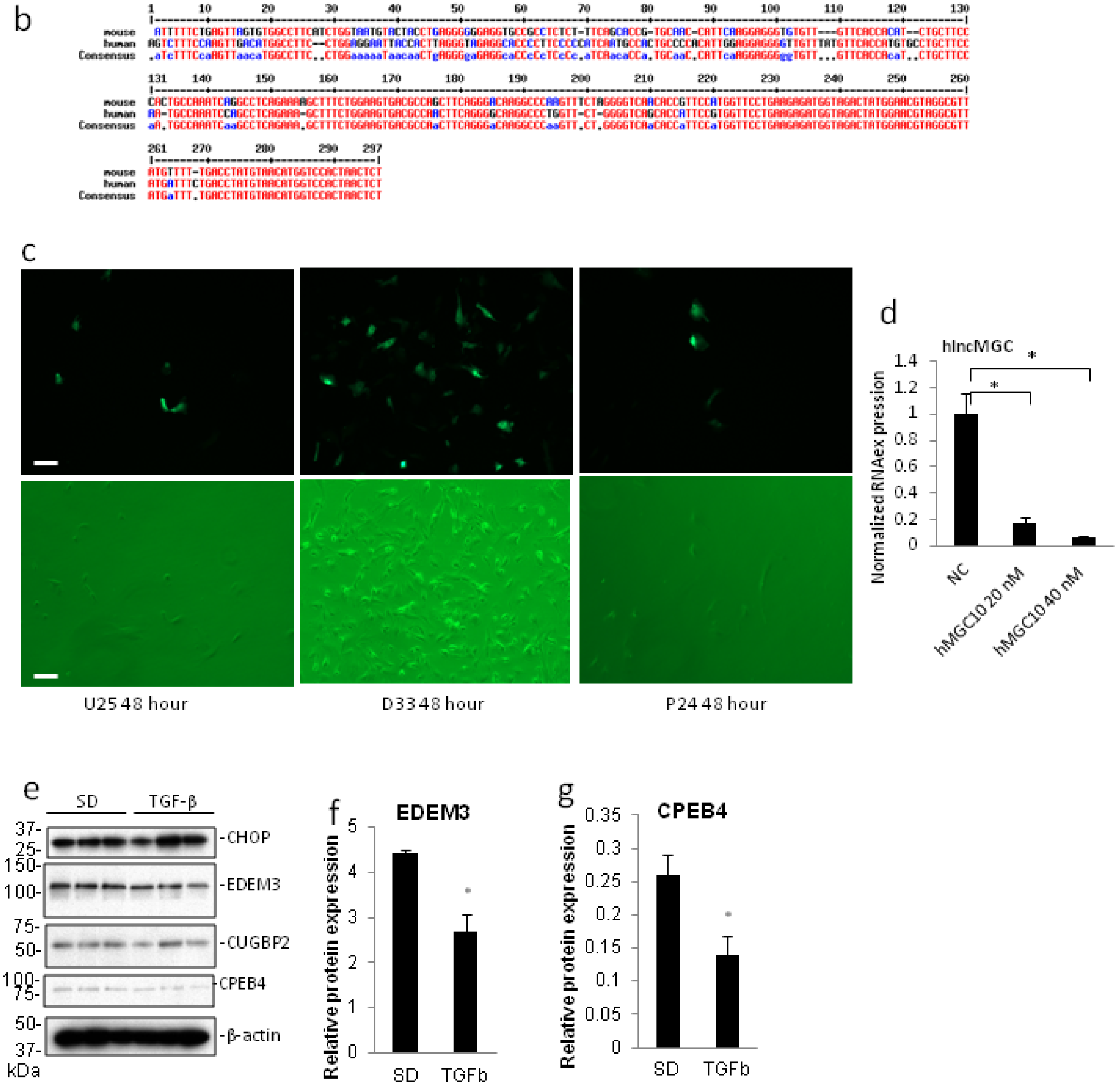

Supplementary Figure 37. Human homologue of lnc-MGC (hlnc-MGC) and its inhibition by HMGC10 in human MC. (a) Genomic sequences of human lnc-MGC. The structure of the promoter region (TATA-like elements and initiator sequence) was conserved. Human target sequence analogous to mouse MGC10 has two base mismatches and the human version of GapmeR (HMGC10) was designed based on the human sequence. Basic chemistry is the same as mouse GapmeR. HMGC10, (GATttggcattggAAG) (uppercase: LNA; lowercase: DNA, full phosphorothioate). (b) Alignment of nucleotide sequences upstream of miR-379 in mouse and human. (c) HMC were transfected with plasmids and/or small RNAs using Nucleofector (Amaxa Biosystems). Basic Nucleofector Kit for Primary Smooth Muscle Cells (Amaxa) was used. Three programs (D33, P24 and U25) were tested to examine the transfection efficiency with pmaxGFP (Amaxa). Program D33 resulted in highest transfection efficiency (~60%) and viability. Scale bar, 20μm. (d) Significant inhibition of hlnc-MGC by HMGC10 in HMC compared to negative control oligo (NC). The upstream region of human miR-379 was examined by RT-PCR in HMC. Two concentrations, 20 and 40 nM of HMGC10 were tested and both significantly inhibited expression of hlnc-MGC. Results are mean + SE in triplicate PCRs of three independent culture experiments. *, $P<0.05$. (e-g) Significant decrease of protein levels of EDEM3 and CPEB4 was observed in HMC treated with TGF-β1 compared to serum depleted control (SD). Wider (uncropped) scans are shown in Supplementary Figure 39.

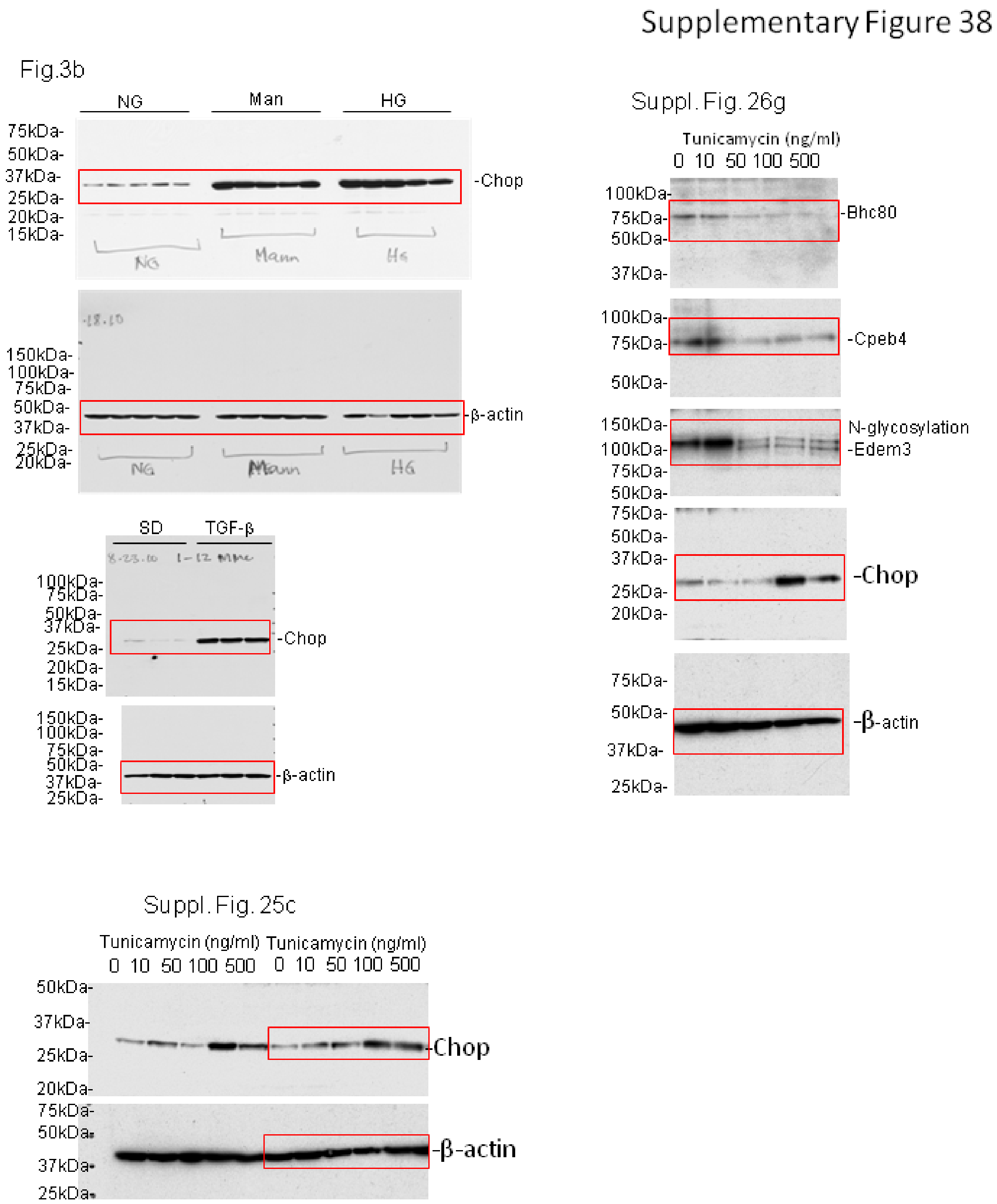

Supplementary Figure 38. Wider (uncropped) scans of blots.

Supplementary Figure 39

Suppl. Fig. 26h

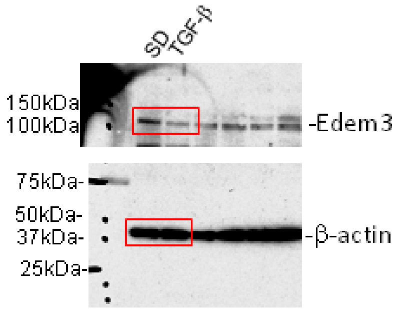

Suppl. Fig. 26i

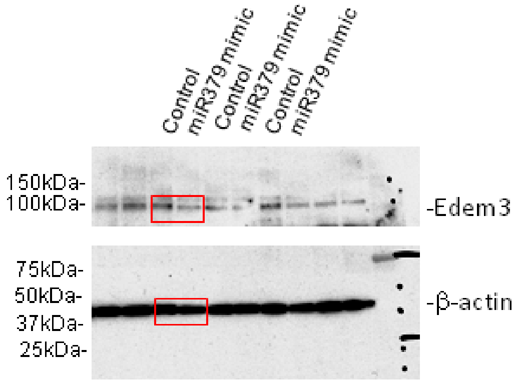

Suppl. Fig.37e

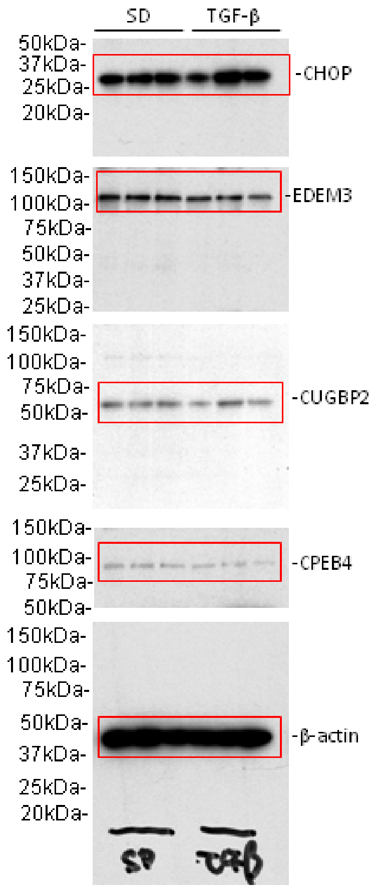

Supplementary Figure 39. Wider (uncropped) scans of blots.

Supplementary Table 1

Abundance of miRNA read frequency (% of all mapped reads per sample) in glomeruli and tubule-interstitial (TI) fractions of kidney biopsies of American Pima Indians.

miRNA	Glomeruli	TI	Gl/TI
Other kidney-enriched miRNAs			
hsa-miR-21	4.67560%	9.51369%	0.5
hsa-miR-192	0.53570%	1.44438%	0.4
hsa-miR-21*	0.00987%	0.02055%	0.5
hsa-miR-192*	0.00250%	0.00740%	0.3
Cluster miRNAs			
hsa-miR-134	0.01867%	0.00725%	2.6
hsa-miR-376c	0.01104%	0.01973%	0.6
hsa-miR-409-3p	0.01033%	0.00797%	1.3
hsa-miR-381	0.00896%	0.01087%	0.8
hsa-miR-411	0.00700%	0.00721%	1
hsa-miR-323b*	0.00697%	0.00143%	4.9
hsa-miR-377	0.00576%	0.01041%	0.6
hsa-miR-379	0.00566%	0.00560%	1
hsa-miR-654	0.00476%	0.00516%	0.9
hsa-miR-382-5p	0.00348%	0.00207%	1.7
hsa-miR-487b	0.00324%	0.00414%	0.8
hsa-miR-376a-1-3p	0.00293%	0.00489%	0.6
hsa-miR-376a-2-3p	0.00293%	0.00489%	0.6
hsa-miR-485-5p	0.00227%	0.00087%	2.6
hsa-miR-409-5p	0.00200%	0.00234%	0.9
hsa-miR-495	0.00180%	0.00152%	1.2
hsa-miR-369	0.00180%	0.00331%	0.5
hsa-miR-323a	0.00164%	0.00138%	1.2
hsa-miR-494	0.00136%	0.00176%	0.8
hsa-miR-299-5p	0.00128%	0.00102%	1.3
hsa-miR-485-3p	0.00127%	0.00066%	1.9
hsa-miR-1185-1-3p	0.00110%	0.00091%	1.2
hsa-miR-410	0.00104%	0.00141%	0.7
hsa-miR-543	0.00103%	0.00076%	1.4
hsa-miR-299-3p	0.00102%	0.00130%	0.8
hsa-miR-654*	0.00101%	0.00040%	2.5
hsa-miR-369*	0.00093%	0.00084%	1.1
hsa-miR-889	0.00084%	0.00118%	0.7
hsa-miR-487a-5p	0.00076%	0.00028%	2.7

hsa-miR-376b	0.00073%	0.00124%	0.6
hsa-miR-1185-2-3p	0.00072%	0.00016%	4.6
hsa-miR-382-3p	0.00067%	0.00089%	0.8
hsa-miR-329-1	0.00062%	0.00057%	1.1
hsa-miR-329-2	0.00062%	0.00057%	1.1
hsa-miR-154-5p	0.00057%	0.00044%	1.3
hsa-miR-758	0.00056%	0.00057%	1
hsa-miR-376a-1-5p	0.00053%	0.00079%	0.7
hsa-miR-487a-3p	0.00050%	0.00054%	0.9
hsa-miR-154-3p	0.00044%	0.00059%	0.8
hsa-miR-411*	0.00041%	0.00056%	0.7
hsa-miR-656	0.00041%	0.00049%	0.8
hsa-miR-655	0.00037%	0.00058%	0.6
hsa-miR-377*	0.00036%	0.00028%	1.3
hsa-miR-376a-2-5p	0.00025%	0.00021%	1.2
hsa-miR-412-5p	0.00024%	0.00017%	1.4
hsa-miR-539	0.00022%	0.00066%	0.3
hsa-miR-1185-1-5p	0.00022%	0.00037%	0.6
hsa-miR-1185-2-5p	0.00022%	0.00037%	0.6
hsa-miR-379*	0.00021%	0.00025%	0.8
hsa-miR-380-3p	0.00020%	0.00020%	1
hsa-miR-323b	0.00016%	0.00055%	0.3
hsa-miR-376c*	0.00015%	0.00020%	0.7
hsa-miR-380-5p	0.00013%	0.00004%	3.7
hsa-miR-376b*	0.00013%	0.00017%	0.8
hsa-miR-539*	0.00010%	0.00014%	0.7
hsa-miR-668	0.00009%	0.00005%	1.7
hsa-miR-134*	0.00008%	0.00006%	1.3
hsa-miR-543*	0.00007%	0.00005%	1.4
hsa-miR-381*	0.00006%	0.00010%	0.6
hsa-miR-544-3p	0.00003%	0.00006%	0.6
hsa-miR-495*	0.00003%	0.00004%	0.6
hsa-miR-487b*	0.00003%	0.00002%	1.6
hsa-miR-544-5p	0.00003%	0.00008%	0.3
hsa-miR-1197	0.00003%	0.00006%	0.4
hsa-miR-758*	0.00002%	0.00002%	1.2
hsa-miR-1193-5p	0.00002%	0.00001%	1.8
hsa-miR-541-5p	0.00002%	0.00001%	2.8
hsa-miR-412-3p	0.00002%	0.00001%	1.5
hsa-miR-323a*	0.00002%	0.00001%	1.6
hsa-miR-496	0.00002%	0.00007%	0.2
hsa-miR-410*	0.00001%	0.00001%	1.7
hsa-miR-668*	0.00001%	0.00000%	6.6

hsa-miR-329-1*	0.00001%	0.00000%	1.7
hsa-miR-329-2*	0.00001%	0.00000%	1.7
hsa-miR-889*	0.00001%	0.00001%	0.6
hsa-miR-1193-3p	0.00001%	0.00000%	1.3
hsa-miR-494*	0.00000%	0.00001%	0.4
hsa-miR-656*	0.00000%	0.00001%	0.3
hsa-miR-496*	0.00000%	0.00000%	0.5
hsa-miR-655*	0.00000%	0.00001%	0
hsa-miR-1197*	0.00000%	0.00001%	0
hsa-miR-541-3p	0.00000%	0.00001%	0

Highlighted miRNAs exhibit significant association of precursors with morphometric parameters (Supplementary Table 2). Of note, for all miRNAs that are associated with morphometric parameters -3p and -5p (or *) sequences are both detected, supporting that the sequence reads represent miRNA expression. Furthermore, several of the highlighted miRNAs have higher relative abundance in glomeruli versus TI compartment.

Supplementary Table 2

Association of miRNA-precursor abundance in human glomeruli with morphometric parameters

Morphometric Parameter	miRNA Precursor	r	p-value
Podocyte Density	Mir-485	-0.43	0.006
	Mir-299	-0.33	0.043
Podocyte Volume	Mir-299	0.34	0.037
GLEPP1 Volume	Mir-409	0.34	0.035
Glomerular Volume	Mir-409	0.36	0.025
	Mir-655	0.34	0.032
	Mir-654	0.32	0.045
Mesangial Index	Mir-380	0.39	0.014
	Mir-299	0.36	0.023
	Mir-889	0.34	0.032
Mesangial Volume	Mir-654	0.35	0.030
	Mir-655	0.32	0.050
Global Glomerulosclerosis	Mir-668	0.52	0.037

. DN in humans is associated with loss of podocytes leading to decreased podocytes density, hypertrophy of podocytes (podocytes and GLEPP1 volume), glomerular hypertrophy (glomerular volume), mesangial expansion (mesangial index and volume) and accumulation of globally sclerotic glomeruli (global glomerulosclerosis). [3-6]

Reference:

Christopher L. O'Connor, Madhusudan Venkatareddy, Su Q. Wang, Laura H. Mariani, Markus Bitzer, Roger C. Wiggins, Jeffrey B. Hodgin. Morphometric Analysis of Podocyte Density and Glomerular Volume Adapted for Routine Diagnostic Biopsy Evaluation Pathology & Internal Medicine, University of Michigan, Ann Arbor, MI. SA-PO481; ASN Kidney Week 2014

Supplementary Table 3

qPCR primers		
**mouse**		
lncMGC INR	Forward	ATTTTTCTGAGTTAGTGTGGCCTTCATCTG
	Reverse	TCAGGAACCATGGAACGGTGTTGACCCCTAG
lncMGC gm	Forward	TTTGAGGAAGAGCCAGTGTC
	Reverse	GCCAAGCCAGAGAAATTTGC
lncMGC mirg	Forward	CCTTCCTGGATCTCTCGCTT
	Reverse	GTGGGAGTTGAAACATGGGT
Tnrc6b	Forward	GGATTGCCTCGGCCTCTACCT
	Reverse	ACCAGCCAGTAACTAGGAcG
Cugbp2 (Celf2)	Forward	GGTCAGATAGAAGAATGCcG
	Reverse	GACAAACGCACAGCCTCGACT
Cpeb4	Forward	AAAAGGAGCTGGAAGAGTCG
	Reverse	TGGTCATCCAAGACATATGGC
Pum2(Pumilio2)	Forward	CGCAAATACACATATGGGAAGC
	Reverse	GCCCACTCTTTGAACATGGT
phf21a (BHC60)	Forward	CACTTACCTTAACAGCACAATGC
	Reverse	CCACTTTTTCTGCAAACACTGC
Edem3	Forward	CACCTTGATCCTCGAGTTTGC
	Reverse	TCGCTGTCTTTTCTCCCAGAG
Chop (Ddit, Gadd153)	Forward	GCACCTATATCTCATCCCCAG
	Reverse	TGCGTGTGACCTCTGTTG
Col1a2	Forward	CAGAACATCACCTACCACTGCAA
	Reverse	TTCAACATCGTTGGAACCCTG
Col4a1	Forward	GCCTTCCGGGCTCCTCAG
	Reverse	TTATCACCAGTGGGTCCG
TGFb1	Forward	GGACTCTCCACCTGCAAGAC
	Reverse	GACTGGCGAGCCTTAGTTTG
Ctgf	Forward	GCGAAGCTGACCTGGAGGA
	Reverse	CGCACGAGTGGTGGTTCTGTGCG
Hspa5	Forward	GGAAAGAAGGTTACCCATGC
	Reverse	AGAAGAGACACATCGAAGGT
PAI1	Forward	GACGCCTTCATTTGGACGAA
	Reverse	CGGACCTTTTCCCTTCAAGAGTCCG
Fn1	Forward	CGGTGGAGTCCTGACACAATCACCG
	Reverse	GCGCCCACCAATCTGAAGT
ATF3	Forward	AACTGGCTTCCTGTGCACTT
	Reverse	TGAGGCCAGCTAGGTCATCT
CypA (Ppia)	Forward	ATGGTCAACCCCACCGTGT

	Reverse	TTCTTGCTGTCTTTGGAACTTTGTC
ChIP primers		
Smad site	Forward	GAGAATCTACAGAGACTGAGAATCTGCACATG
	Reverse	GGTCTGAAACATCTCCATCCAGTCTGG
Chop site	Forward	GAGCTCTTGCTCTTTGCACCTGCG
	Reverse	AAGCAGGTGGAACCAGAAGTAAGCC
XBP1 splicing	Forward	ACACGCTTGGGAATGGACAC
	Reverse	CCATGGGAAGATGTTCTGGG
**Human**		
hlncMGC	Forward	GCCTGCTTCCAATGCCAAATC
	Reverse	CTTCAGGAACCACGGAATGGT
hEDEM3	Forward	GATGGAGAAGGATGAGCTATGAC
	Reverse	GAACGTGGTTGTTCATCACC
hCPEB4	Forward	GATTGATACCGACCCTGAGC
	Reverse	ATATGGCTTAACTTCCACCCG
hCUGBP2 (CELF2)	Forward	TGCTTCAACCCCCAACTCC
	Reverse	GTCCTTGCAGAGTCCCGAGA
hTGFb1	Forward	CCTGCCCCTACATTTGGAG
	Reverse	CCGGGTTATGCTGGTTGTAC
hCOL1A2	Forward	ACTGTTCTTGTAGATGGCTGC
	Reverse	CAAAGAATTCCTGGTCAGCAC
hCOL4A1	Forward	CGTGGGACCTGCAATTACTA
	Reverse	TTCTCATACAGACTTGGCAGC
hFN1	Forward	GCCAGTCCTACAACCAGTATTC
	Reverse	CTTCTCTGTCAGCCTGTACATC
hCTGF	Forward	ACCAATGACAACGCCTCC
	Reverse	TTGGAGATTTTGGGAGTACGG
hATF3	Forward	CCTCTGCGCTGGAATCAGTC
	Reverse	TTCTTTCTCGTCGCCTCTTTTT
hCypA (PPIA)	Forward	CCCACCGTGTTCTTCGACATT
	Reverse	GGACCCGTATGCTTTAGGATGA

Supplementary references

1 Lewis, B. P., Burge, C. B. & Bartel, D. P. Conserved seed pairing, often flanked by adenosines, indicates that thousands of human genes are microRNA targets. *Cell* **120**, 15-20, (2005).

2 Liang, X. H., Shen, W., Sun, H., Prakash, T. P. & Crooke, S. T. TCP1 complex proteins interact with phosphorothioate oligonucleotides and can co-localize in oligonucleotide-induced nuclear bodies in mammalian cells. *Nucleic Acids Res* **42**, 7819-7832, (2014).

3 Hodgin, J. B. *et al.* Glomerular Aging and Focal Global Glomerulosclerosis: A Podometric Perspective. *Journal of the American Society of Nephrology* **26**, 3162-3178, (2015).

4 Miyauchi, M. *et al.* Hypertrophy and Loss of Podocytes in Diabetic Nephropathy. *Internal medicine* **48**, 1615-1620, (2009).

5 Kato, M. & Natarajan, R. Diabetic nephropathy--emerging epigenetic mechanisms. *Nat Rev Nephrol* **10**, 517-530, (2014).

6 Kato, M. & Natarajan, R. MicroRNAs in diabetic nephropathy: functions, biomarkers, and therapeutic targets. *Annals of the New York Academy of Sciences* **1353**, 72-88, (2015).
